# A multimodal generative AI copilot for human pathology

**DOI:** 10.1038/s41586-024-07618-3

**Published:** 2024-06-12

**Authors:** Ming Y. Lu, Bowen Chen, Drew F. K. Williamson, Richard J. Chen, Melissa Zhao, Aaron K. Chow, Kenji Ikemura, Ahrong Kim, Dimitra Pouli, Ankush Patel, Amr Soliman, Chengkuan Chen, Tong Ding, Judy J. Wang, Georg Gerber, Ivy Liang, Long Phi Le, Anil V. Parwani, Luca L. Weishaupt, Faisal Mahmood

**Affiliations:** 1 Department of Pathology, Brigham and Women’s Hospital, Harvard Medical School, Boston, MA USA; 2 Department of Pathology, Massachusetts General Hospital, Harvard Medical School, Boston, MA USA; 3 Cancer Program, Broad Institute of Harvard and MIT, Cambridge, MA USA; 4 Electrical Engineering and Computer Science, Massachusetts Institute of Technology (MIT), Cambridge, MA USA; 5 Department of Pathology, Wexner Medical Center, Ohio State University, Columbus, OH USA; 6 Department of Pathology, Pusan National University, Busan, South Korea; 7 Department of Laboratory Medicine and Pathology, Mayo Clinic, Rochester, MN USA; 8 Harvard John A. Paulson School of Engineering and Applied Sciences, Harvard University, Cambridge, MA USA; 9 Health Sciences and Technology, Harvard-MIT, Cambridge, MA USA; 10 Harvard Data Science Initiative, Harvard University, Cambridge, MA USA

**Keywords:** Machine learning, Pathology, Image processing, Data integration

## Abstract

Computational pathology^1,2^ has witnessed considerable progress in the development of both task-specific predictive models and task-agnostic self-supervised vision encoders^3,4^. However, despite the explosive growth of generative artificial intelligence (AI), there have been few studies on building general-purpose multimodal AI assistants and copilots^5^ tailored to pathology. Here we present PathChat, a vision-language generalist AI assistant for human pathology. We built PathChat by adapting a foundational vision encoder for pathology, combining it with a pretrained large language model and fine-tuning the whole system on over 456,000 diverse visual-language instructions consisting of 999,202 question and answer turns. We compare PathChat with several multimodal vision-language AI assistants and GPT-4V, which powers the commercially available multimodal general-purpose AI assistant ChatGPT-4 (ref. ^6^). PathChat achieved state-of-the-art performance on multiple-choice diagnostic questions from cases with diverse tissue origins and disease models. Furthermore, using open-ended questions and human expert evaluation, we found that overall PathChat produced more accurate and pathologist-preferable responses to diverse queries related to pathology. As an interactive vision-language AI copilot that can flexibly handle both visual and natural language inputs, PathChat may potentially find impactful applications in pathology education, research and human-in-the-loop clinical decision-making.

## Abstract (web summary)

PathChat, a multimodal generative AI copilot for human pathology, has been trained on a large dataset of visual-language instructions to interactively assist users with diverse pathology tasks.

## Main

Computational pathology has witnessed a notable transformation in recent years. This has been propelled by the convergence of several key trends including increased availability and institutional adoption of digital slide scanning, rapid progress in artificial intelligence (AI) research, increased accessibility of large datasets and substantial high-performance computing resources^1,2,7^. With varying degrees of success, researchers have leveraged deep learning to address a diverse range of tasks, including cancer subtyping^8,9^ and grading^10,11^, metastasis detection^12^, survival^13–17^ and response-to-treatment prediction^18,19^, tumour site of origin prediction^20,21^, mutation prediction and biomarker screening^22–24^, and more^25^. Moreover, general-purpose vision-encoder models^26^, which are trained on vast datasets of unlabelled histopathology images and can serve as versatile task-agnostic model backbones^3,4^, are paving the way for further improvements across many tasks in computational pathology, both in performance and label efficiency.

However, the aforementioned developments in computational pathology do not yet reflect the important roles of natural language in pathology, which acts as a key to unlocking rich, diverse sources of accumulated human medical knowledge, a supervisory signal for model development and a unified medium for facilitating intuitive interaction between powerful AI models and end users. Notably, in general machine learning, representative works^27,28^ have demonstrated that large-scale vision-language representation learning can augment vision-only AI models with new capabilities, including zero-shot image recognition and text-to-image retrieval. Depending on the architectural design, training data and objectives, pretrained visual-language systems can often be fine-tuned for tailored tasks ranging from answering visual questions and image captioning to object detection and semantic segmentation. In medical imaging and computational pathology, researchers have recently begun to harness diverse sources^29–33^ of paired biomedical images and captions or reports for visual-language pretraining, including the development of CLIP-like^27^ models tailored for specific domains such as pathology^30,33–35^ and radiology^36–38^. In computational pathology, a few works have shown promising zero-shot performance in select diagnostic and retrieval tasks^30,33,34^. Other researchers have experimented with specialized models for answering biomedical visual questions or image captioning^39–43^. However, for pathologists, researchers using pathology image data and pathology trainees alike, these models are not yet ready to serve as interactive companions (or copilots) that can follow diverse instructions and coherently and accurately answer complex open-ended questions posed in natural language.

Following the rise of large language models (LLMs)^44–47^, rapid advances in multimodal LLMs (MLLMs)^5,48,49^ and the broader field of generative AI^50^ are poised to open a new frontier for computational pathology, one that emphasizes natural language and human interaction as key components of AI model design and user experience, in addition to powerful visual processing capabilities. Multimodal generative AI products such as ChatGPT have demonstrated impressive capabilities on a wide range of routine, creative and professional use cases^6,51^, including coding, writing, summarization, data analysis, answering questions, translation and even image generation, while being accessible through an intuitive and interactive user interface. Although there have been attempts to investigate their performance on answering medicine-related queries, their capability to assist professionals and researchers in the highly specialized but important subfield of anatomic pathology remains relatively unexplored^52–57^. Yet, the potential applications of an interactive multimodal AI copilot for pathology are immense. The ability to understand and respond to complex queries in natural language could, in theory, enable such a copilot for pathology to serve as a helpful companion across various stages of human-in-the-loop clinical decision-making, education and research. For instance, an AI copilot would be able to ingest a histopathology image, provide an initial assessment of the morphological appearance and identify potential features of malignancy. Subsequently, a pathologist or trainee could provide more context about the underlying case, such as clinical parameters of the patient and the tissue site, and ask the model to suggest a differential diagnosis. If deemed reasonable, the user could then request helpful suggestions for ancillary testing and immunohistochemical (IHC) stains to narrow down the differential. Finally, the results of such tests could also be provided to the model, which would then make a final deduction and arrive at a diagnosis. In research, a multimodal AI copilot that can summarize the morphological features of large cohorts of histopathology images would potentially enable automated quantification and interpretation of morphological markers in large data cohorts. In medical education, an accurate on-demand interactive AI companion could help democratize access to expert-level guidance and training in pathology, thereby narrowing the gap between regional disparities in healthcare provision.

## A multimodal generative AI copilot for human pathology

**Fig. 1:**
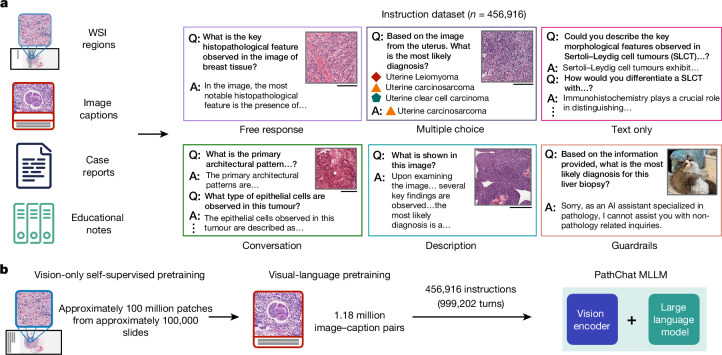
Curation of instruction-following dataset and PathChat overview. **a**, We curated what is presently the largest instruction fine-tuning dataset specialized for pathology. It consists of 456,916 instructions and corresponding responses covering various formats (for example, multi-turn conversations, multiple-choice questions and short answers; see Extended Data Fig. 1 for complete examples) from diverse sources. **b**, To build an MLLM-based vision-language AI assistant that can reason over visual and natural language inputs, we began with a SOTA, vision-only, self-supervised, pretrained, foundation, encoder model, UNI and performed further vision-language pretraining analogous to CONCH. The resulting vision encoder was subsequently connected to a 13-billion-parameter, pretrained, Llama 2 LLM through a multimodal projector module (not shown) to form the complete MLLM architecture. The MLLM was fine-tuned on the curated instruction-following dataset to build PathChat, a vision-language AI assistant specialized for human pathology. More details about data curation and model training can be found in ‘Curation of the PathChat dataset’ and ‘Design and training of the PathChat model’ in Methods, respectively. Scale bars, 200 µm.

**Extended Data Fig. 1:**
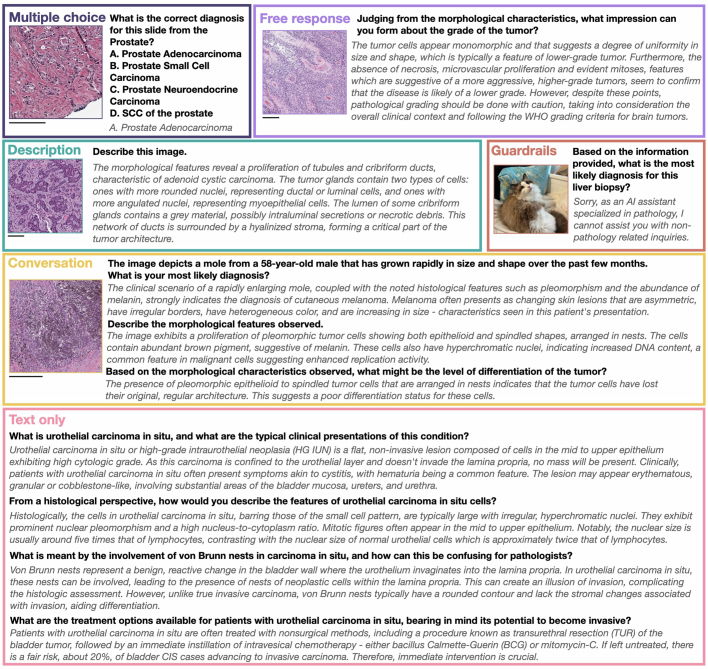
Examples of instructions for finetuning MLLM. An example of each of six different types of instructions to develop PathChat via instruction finetuning is illustrated. Bolded texts represent instructions provided to the model while italicized texts represent the reference outputs the model is expected to output during training. More details on dataset curation are provided in the **PathChat dataset curation** section of **Methods**. Scale bars are 200 µm.

In this article, we develop PathChat, a multimodal generative AI copilot for human pathology powered by a custom fine-tuned MLLM. To build an MLLM that can reason over both visual and natural language inputs, we began with UNI^3^, a state-of-the-art (SOTA) vision-only encoder pretrained on over 100 million histology image patches from over 100,000 slides using self-supervised learning. We performed further vision-language pretraining on the UNI encoder with 1.18 million pathology image and caption pairs to align its image representation space with that of pathology text^34^. The resulting vision encoder was subsequently connected to a 13-billion-parameter pretrained, Llama 2 LLM^46^ through the multimodal projector module to form the complete MLLM architecture (see ‘Design and training of the PathChat model’ in Methods for more details). The MLLM was finally fine-tuned using a curated dataset of over 450,000 instructions to build PathChat (Fig. 1 and Extended Data Fig. 1), which can understand pathology images and text and respond to complex pathology-related queries. More information about data curation and model training can be found in ‘Curation of the PathChat dataset’ and ‘Design and training of the PathChat model’ in Methods, respectively, with further details summarized in Supplementary Tables 1–4.

**Fig. 2:**
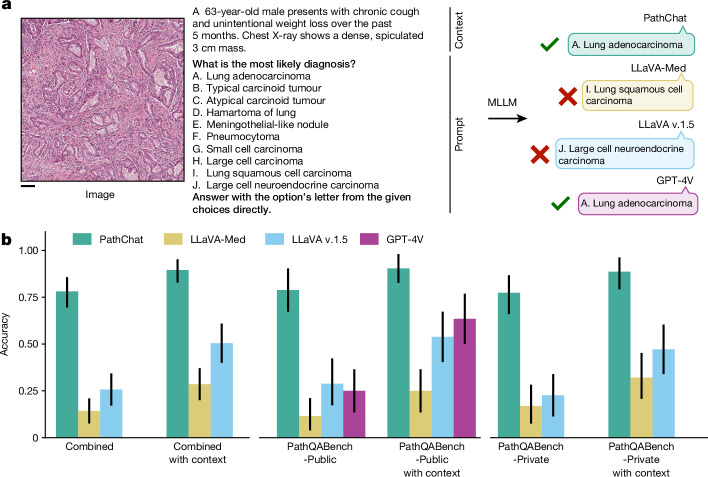
Multiple-choice evaluation of PathChat. **a**, Illustrative example of a multiple-choice diagnostic question. The input always includes a salient ROI of an histology image selected by a board-certified anatomic pathologist and an instruction to select the most probable diagnosis from a set of possible choices. In the image + clinical context evaluation setting, which was designed to more closely mimic a real-world diagnostic workflow, relevant clinical context (designed by the pathologist, shown in blue) is provided together with the histology image and prepended to the original question. Scale bar, 200 µm. **b**, Accuracy of MLLMs on multiple-choice diagnostic questions. Combined (*n* = 105 questions), PathQABench-Public (*n* = 52) and PathQABench-Private (*n* = 53). Note that we compare against GPT-4V only for questions based on publicly available cases (PathQABench-Public). Error bars represent 95% confidence intervals, and the centres represent the computed accuracy.

**Fig. 3:**
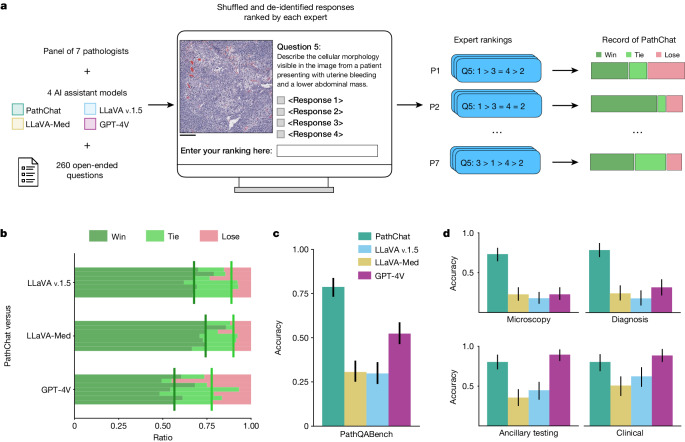
Open-response evaluation of PathChat and reader study from a panel of seven pathologists. **a**, Evaluation workflow for ranking model outputs for open-ended questions. A panel of seven pathologists were recruited to assess the model responses for the 260 open-ended questions. The ordering of responses by the four AI assistant models were randomly shuffled for each question and each pathologist independently ranked them for all questions while being blinded to which model produced which response (see ‘MLLM evaluation’ in Methods for more details). Scale bar, 200 µm. **b**, Head-to-head records on open-ended questions for PathChat versus other MLLMs evaluated by seven pathologists independently. Win, PathChat was ranked higher than the model. Tie, PathChat tied with the model in terms of ranking. Lose: Said model was ranked higher than PathChat. Vertical bars represent median win rate (dark green) across all seven pathologists and median win + tie rate (light green). **c**, Accuracy of MLLMs on a subset (*n* = 235 questions) of open-ended questions for which two pathologists reached a consensus after discussing independent evaluations of model responses. **d**, Accuracy for different categories of questions on the consensus subset. Microscopy (*n* = 101), diagnosis (*n* = 79), clinical (*n* = 61) and ancillary testing (*n* = 76). Each question could belong to more than one category. In **c**,**d**, error bars represent 95% confidence intervals, and the centres represent the computed accuracy.

We demonstrate the capabilities of PathChat in various applications including an analysis of pathology cases from diverse organ sites and practices (Figs. 2 and 3). Additionally, we curated a high-quality benchmark for open-ended visual pathology questions suitable for evaluating the performance of MLLMs in pathology, which we curated with expert supervision (see ‘Benchmark for expert-curated pathology questions’ in Methods for more details). We compare PathChat to both LLaVA^5^, a SOTA general-domain open-source MLLM, and LLaVA-Med^53^, which has been tailored to the biomedical domain. We also compare it with a SOTA commercial solution, ChatGPT-4 (powered by GPT-4V), despite our model being significantly smaller and cheaper to serve.

## Performance on multiple-choice diagnostic questions

We began by assessing the capability of our PathChat MLLM to directly make a diagnosis based on histology images. For this purpose, a board-certified pathologist manually selected salient regions of interest (ROIs) from routine diagnostic whole-slide images (WSIs) stained with haematoxylin and eosin (H&E) from both The Cancer Genome Atlas (TCGA) and our in-house pathology archive (both of which were completely withheld from model pretraining or fine-tuning). The questions covered 54 diagnoses from 11 different major pathology practices and organ sites (Supplementary Tables 5 and 6). For each organ system, the pathologist selected a set of ten possible answers that encompassed the correct answers for all questions within that organ system as well as other relatively common diagnoses within that organ system (Supplementary Table 7). For each question, we considered two evaluation strategies. In the first (image-only setting), the model was presented with only the image and the multiple-choice question as input. In the second (image with clinical context), the model was also presented with the clinical context to closely mimic a real-world diagnostic workflow, in which information such as patient age, sex, clinical history and radiology findings are included with the histology image for the clinical case. In both settings, the model was assessed on its ability to accurately select the ground truth diagnosis from the set of possible options. We provide an illustrative example of the complete model input in Fig. 2a. For all cases (denoted as ‘Combined’ in Fig. 2b), we compared PathChat against LLaVA 1.5, a SOTA general-purpose visual-language chatbot assistant, and LLaVA-Med, a specialized version of LLaVA fine-tuned for answering biomedical-related queries. For the subset of 52 cases derived from publicly available WSIs (denoted as PathQABench-Public), in addition to LLaVA 1.5 and LLaVA-Med, we also compared PathChat with GPT-4V, which powers ChatGPT-4, one of the current best-in-class vision-capability-enabled commercial AI assistants, which was developed by OpenAI. All models were evaluated as is without any task-specific fine-tuning, consistent with the paradigm of zero-shot transfer.

**Extended Data Fig. 2:**
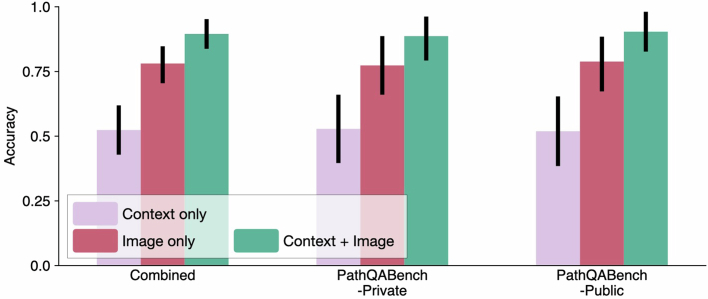
Utilization of visual input and clinical context in multiple choice diagnostic questions. On the multiple choice diagnostic benchmarks (Combined, *n* = 105 questions; PathQABench-Private, *n* = 53; PathQABench-Public, *n* = 52), we investigated whether PathChat can effectively leverage both unstructured clinical context in the form of natural language as well as visual features in the image ROI instead of deriving its answer solely based on either input alone. In the context only setting, the clinical context is provided to the model but the image is not provided (see Fig. 2a for an example multiple choice question that contains the clinical context, the choices, and the image). On the flip side, in the image only setting, the clinical context is not provided, and the model is asked to infer the correct diagnosis from the possible choices based solely on the image. We observed that PathChat achieves maximum performance when both clinical context and the image are provided. Error bars represent 95% confidence intervals, and the centers represent the computed accuracy.

In both evaluation settings (image-only and image with clinical context), PathChat convincingly outperformed the open-source baselines LLaVA 1.5 and LLaVA-Med in terms of diagnostic accuracy (Fig. 2a and Supplementary Tables 8–10). In the image-only evaluation setting, PathChat scored an accuracy of 78.1% (+52.4% versus LLaVA 1.5 and +63.8% versus LLaVA-Med, *P* *<* 0.001 for both) on the full combined benchmark. In line with our expectation, the accuracy of PathChat improved to 89.5% (+39.0% versus LLaVA 1.5 and +60.9% versus LLaVA-Med, *P* *<* 0.001 for both) when useful clinical context was provided. Specifically, note that the addition of clinical context consistently improved the accuracy of PathChat for both the private in-house cases (PathQABench-Private, +11.3%) and the public TCGA cases (PathQABench-Public, +11.6%). On the other hand, when only the clinical context was provided (the corresponding image was not shown to the model), its performance was substantially lower (Extended Data Fig. 2), which suggests that PathChat derives substantial predictive power from visual features and does not rely on the clinical context alone. Together, these findings suggest that PathChat can effectively and flexibly leverage multimodal information to provide a more accurate diagnosis of histology images than when simply given such non-visual information in plain natural language without specialized data processing.

Additionally, using PathQABench-Public, which contains cases only from the publicly available TCGA WSIs, we also compared our model against the GPT-4Vision (GPT-4V) model. Given that we do not know the extent to which GPT-4V has been trained on histopathology-specific data from the internet, our use of manually curated ROIs from WSIs for evaluation helps to minimize the likelihood of data contamination and ensure a proper assessment of its performance on histopathology images. Note that guardrails appear to have been implemented into GPT-4V to prevent it from sometimes addressing queries that require an examination of medical images. In that case, it informs the user that it cannot provide a pathology interpretation and recommends consulting a medical professional. In such cases, we made a maximum of two further submissions with the same query for a total of up to three attempts (see ‘Evaluating GPT-4V’ in Methods for more details). Following this evaluation protocol, we successfully queried GPT-4V for 47 out of 52 PathQABench-Public images when clinical context was included (28 out of 52 questions for the image-only setting). An ultimately unsuccessful query was treated as incorrect as the response did not address the question. Although GPT-4V was more accurate than the open-source MLLMs when clinical context was provided, our domain-specific PathChat MLLM was consistently better in both evaluation settings (90.5% versus 63.5% by GPT-4V with clinical context, +26.9%; 78.8% versus 25% by GPT-4V for image-only, +53.8%; *P* *<* 0.001 for both). Although a part of this difference may be explained by GPT-4V’s guardrails, for a more comprehensive and transparent assessment, we also reported performance on only the subset of questions that GPT-4V successfully answered (Supplementary Table 11) and found that PathChat still consistently outperformed GPT-4V by a relatively large margin (+21.3%, *P* = 0.003 on 47 questions with clinical context; +32.2%, *P* = 0.014 on 28 questions for the image-only setting).

## Performance on answering open-ended questions

Beyond multiple-choice diagnostic questions, it is valuable to assess the ability of PathChat and other MLLMs to generate coherent, reasonable and clinically relevant responses to open-ended pathology-related inquiries (‘Benchmark for expert-curated pathology questions’ in Methods). Based on cases from PathQABench-Public, a board-certified anatomic pathologist carefully curated open-ended questions targeting a broad spectrum of topics including microscopy image description, histologic grade and differentiation status, risk factors, prognosis, treatment, diagnosis, IHC tests, molecular alterations and other tests. As with the multiple-choice evaluation, to mimic the real-world use case of a pathology AI assistant, each question was provided to models as is, without any further model or task-specific fine-tuning.

**Extended Data Fig. 3:**
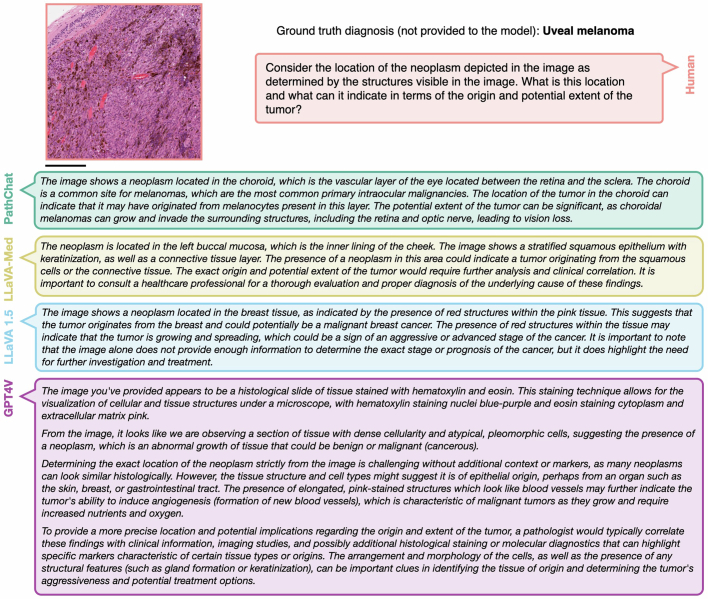
Comparing model outputs on open-ended question answering, example 1. An example question in PathQABench-Public regarding uveal melanoma, for which the response by PathChat is ranked higher (considered more preferable by expert pathologists) than other models as it clearly, correctly, and fully addresses the query. The other models give incorrect locations that the image is from, give an incorrect description of the image, or are so general as to be unhelpful. Scale bar is 200 µm.

**Extended Data Fig. 4:**
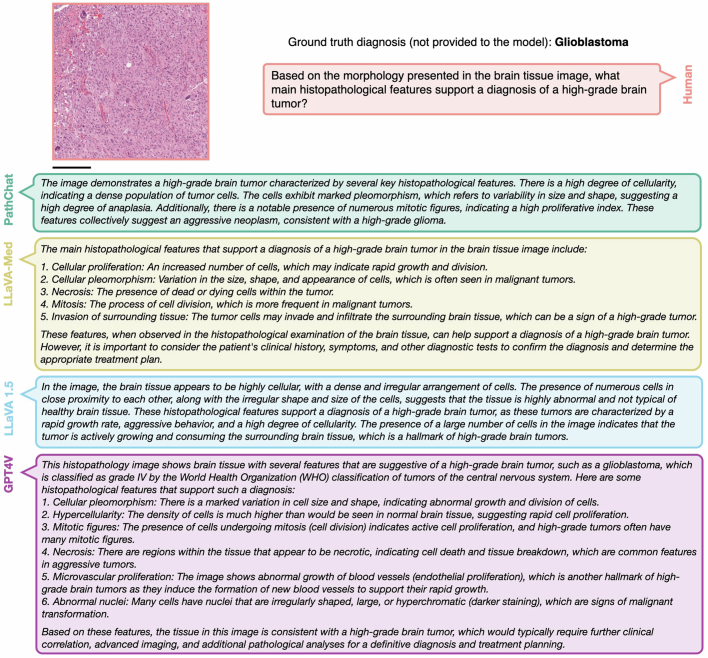
Comparing model outputs on open-ended question answering, example 2. An example question in PathQABench-Public regarding glioblastoma for which the responses by all models were considered to be of roughly comparable quality by expert pathologists for all producing a reasonable and reasonably accurate response to the query, though with some variation between them. Scale bar is 200 µm.

**Extended Data Fig. 5:**
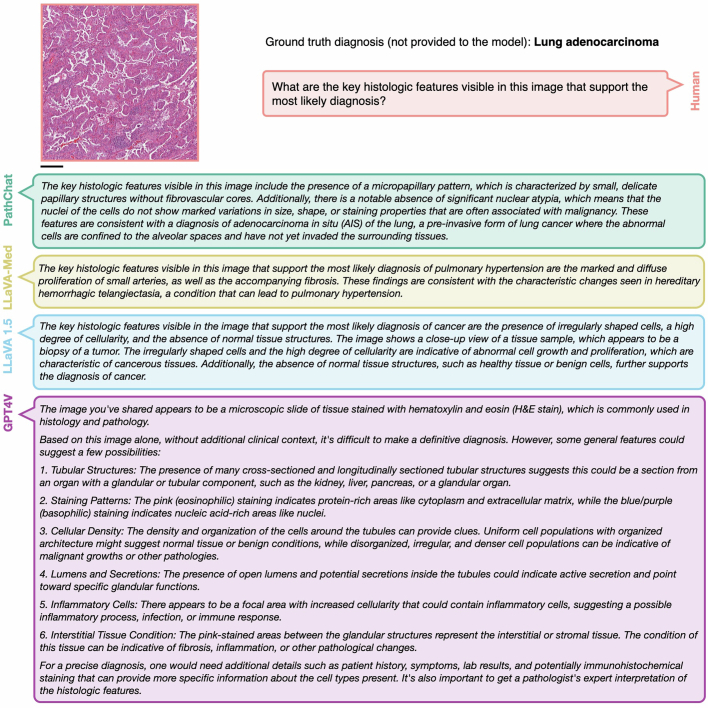
Comparing model outputs on open-ended question answering, example 3. An example question in PathQABench-Public regarding lung adenocarcinoma where all four models performed poorly. None of the four models accurately describe the image or produce the correct diagnosis. Scale bar is 200 µm.

Given the more subjective nature of evaluating responses to open-ended questions, our evaluation consisted of two components. First, seven expert pathologists each ranked (from best to worst, ties allowed) the responses from different models for all questions (Fig. 3a) based on their relevance to the question, correctness and whether it was supplemented with a correct explanation or reasoning in a succinct manner (see ‘MLLM evaluation’ in Methods for more details and Extended Data Figs. 3–5 for illustrative examples of ranked model responses). Throughout the ranking process, the pathologists, who had no previous interaction with any of the models, were also blinded to which model produced which response. Moreover, the responses for each question were randomly shuffled to minimize potential bias towards specific models. This part of the evaluation was aimed at capturing a wide range of expert judgement (including subjective human preference) on the responses.

Overall, we found that PathChat produced on average more preferable, higher-ranked responses than all the other MLLMs tested. When considering head-to-head records (for example, PathChat versus GPT-4V) for model ranking judged by a human expert, a ‘win’ for PathChat on a question equated to PathChat’s response being ranked strictly higher than those of its counterparts. Similarly, a ‘tie’ for PathChat meant that the two models received the same rank, whereas a ‘lose’ meant that PathChat was ranked strictly lower. Against the runner-up GPT-4V, PathChat had a favourable median win rate of 56.5% for the seven independent pathologist evaluators compared to a median lose rate of just 22.3% and a median tie rate of 21.2% (Fig. 3b and Supplementary Tables 12 and 13). Once again, we observed an even larger performance gap in favour of PathChat compared to LLaVA 1.5 (median win rate of 67.7%, median lose rate of 11.2% and median tie rate of 21.5%) and LLaVA-Med (median win rate of 74.2%, median lose rate of 10.0% and median tie rate of 15.4%).

**Extended Data Fig. 6:**
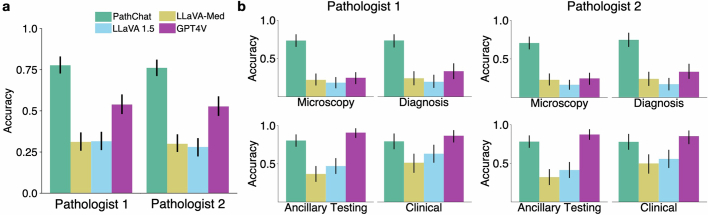
Individual pathologist evaluation of open response performance. **a**. Accuracy of MLLMs on open-ended questions (*n* = 260) as evaluated by two pathologists. See Fig. 3c,d for accuracy on the subset of open-ended questions for which the two pathologists reached a consensus. See **MLLM evaluation** in **Methods** for details. **b**. Accuracy on different categories of questions as rated by two pathologists. Microscopy (*n* = 109), Diagnosis (*n* = 87), Clinical (*n* = 68), Ancillary Testing (*n* = 87). Each question may belong to more than one category. a, b: Error bars represent 95% confidence intervals, and the centers represent the computed accuracy.

Additionally, to establish a more objective metric for each model’s accuracy on the open-ended questions, two board-certified pathologists independently reviewed responses for each question. They assigned a binary label of correct versus incorrect for each model (while remaining blinded to each model’s identity). To mitigate the extent of subjectivity, the two pathologists then discussed all questions where they disagreed in their assessment, in an attempt to reach a consensus. For 235 out of 260 questions, complete agreement was reached for all models, and we used the consensus as the ground truth to compute the accuracy for each model. Specifically, PathChat scored an overall accuracy of 78.7% on the subset of open-ended questions for which the pathologists were able to reach a consensus (Fig. 3c and Supplementary Table 14), which corresponds to a 26.4% improvement (*P* *<* 0.001) compared to the accuracy of 52.3% achieved by the runner-up, GPT-4V. Compared to the publicly available general-purpose MLLM LLaVA 1.5 (accuracy of 29.8%) and the biomedicine-specialized MLLM LLaVA-Med (accuracy of 30.6%), the margin of improvement was even more substantial, at +48.9% and +48.1%, respectively (*P* *<* 0.001 for both). We show the accuracy of each model as assessed by each pathologist on the full set of questions (including the remaining questions for which disagreement remained) in Extended Data Fig. 6.

**Extended Data Fig. 7:**
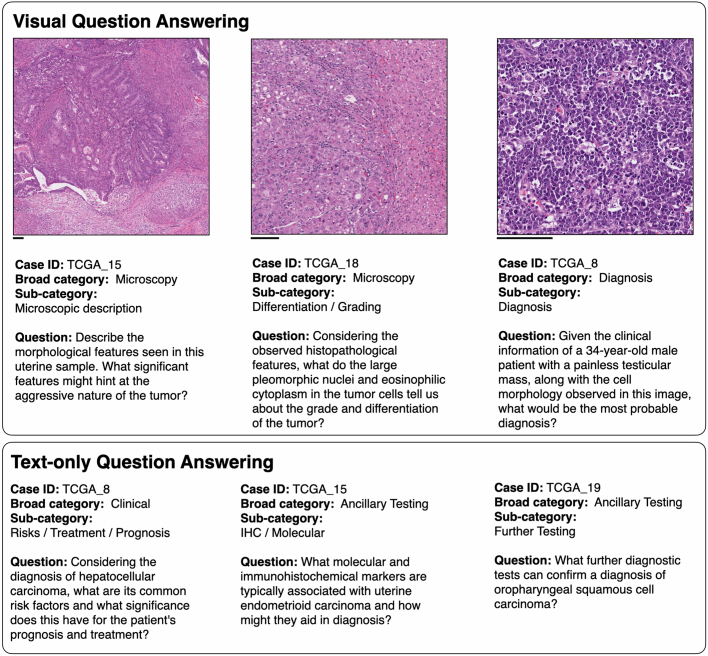
Example questions from PathQABench-Public. PathQABench contains 260 high quality, expert reviewed, open-ended questions created using cases from PathQABench-Public, aimed at assessing a wide range of skills relevant to the practice of pathology. Each question is assigned one or more broad and sub-category based on the topics and skills that it aims to assess. The broad categories are “Microscopy”, “Diagnosis”, “Clinical” and “Ancillary testing”. A detailed description of each category is included in Supplementary Data Table 15. Scale bars are 200 µm.

These results demonstrate that overall, PathChat generated both more accurate as well as more preferable responses to diverse pathology-related queries. Additionally, to better understand the relative strengths and weaknesses of the different models, we analysed their performance for various subgroups of questions (described in Supplementary Tables 15 and 16 with examples provided in Extended Data Fig. 7). In particular, the microscopy category includes questions that test the ability of models to generate accurate and detailed morphological descriptions of histology microscopy images and assess clinically relevant features such as tumour differentiation and grade. Questions in the diagnosis category tested the ability of the models to directly suggest a reasonable diagnosis based on the histology image available and relevant clinical context (unlike the multiple-choice questions for which possible choices are provided). The clinical questions tested the ability to retrieve clinically relevant background knowledge about the disease in question, including risk factors, prognosis and treatment. Ancillary testing questions tested the ability of the models to suggest further testing, such as IHC and molecular workups, to confirm a specific diagnosis or inform prognosis and treatment.

**Extended Data Fig. 8:**
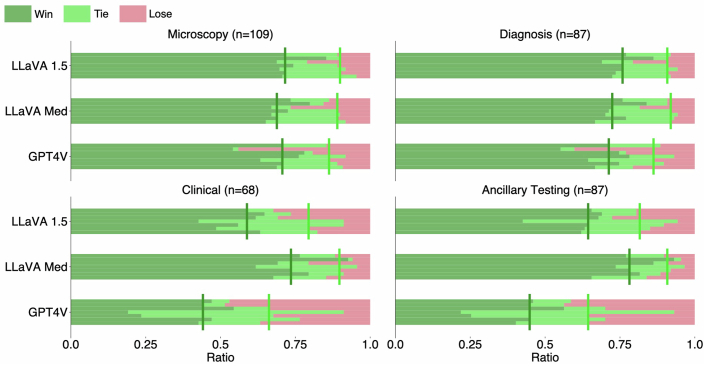
Performance on PathQABench open-ended questions stratified by broad categories. We analyze the head-to-head performance of PathChat against other MLLMs in each broad category of questions evaluated by 7 pathologists independently. For each competing model (LLaVA 1.5, LLaVA-Med, GPT4V), we compute the win/tie/lose rate of PathChat against said model. Win (dark green): PathChat is ranked higher than the model; Tie (light green): PathChat is tied with the model in ranking; Lose (red): PathChat is ranked lower than the model. Vertical bars represent median win rate (dark green) across all 7 pathologists and median win+tie rate (light green).

**Extended Data Fig. 9:**
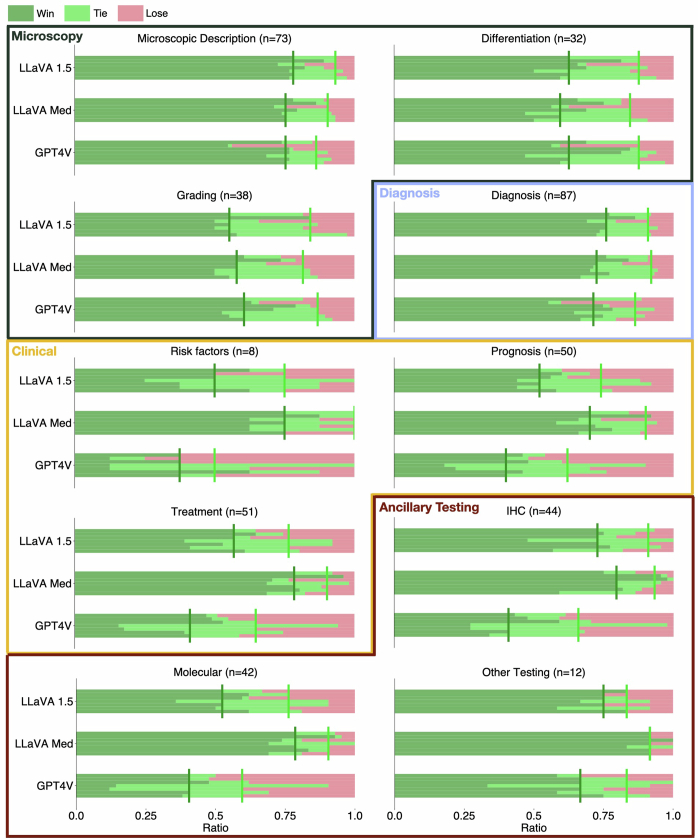
Performance on PathQABench open-ended questions stratified by sub-categories. We further analyze the head-to-head performance of PathChat against other MLLMs in each sub-category of questions evaluated by 7 pathologists independently. For each competing model (LLaVA 1.5, LLaVA-Med, GPT4V), we compute the win/tie/lose rate of PathChat against said model. Win (dark green): PathChat is ranked higher than the model; Tie (light green): PathChat is tied with the model in ranking; Lose (red): PathChat is ranked lower than the model. Vertical bars represent median win rate (dark green) across all 7 pathologists and median win+tie rate (light green).

Although GPT-4V was the runner-up to PathChat overall, PathChat’s responses were especially superior to those of GPT-4V in the categories that require examination of the histology image (microscopy and diagnosis), for which the accuracies on the consensus subset were 73.3% and 78.5% for PathChat respectively versus 22.8% and 31.6% for GPT-4V (Fig. 3d and Supplementary Tables 17–19). Similarly, the median head-to-head win rate against GPT-4V reached 70.6% and 71.3% on these two categories of questions, respectively, compared to the average median win rate of 57.4%. Coupled with a median lose rate against GPT-4V of only 13.8% on both these categories, the results imply that PathChat was better than or as good as GPT-4V in around 86% of queries that emphasize histology image examination (Extended Data Figs. 8 and 9 and Supplementary Tables 20–27). On the other side, we found that PathChat lagged somewhat behind GPT-4V on clinical and ancillary testing, for which, for the consensus subset, PathChat achieved a respectable 80.3% accuracy on both categories compared to GPT-4V’s higher scores of 88.5% and 89.5% on the two categories, respectively. Note that although PathChat convincingly outperformed GPT-4V in accuracy on the microscopy and diagnosis categories according to the consensus (*P* < 0.001 for both, *n* = 101 and 79, respectively), we did not find any statistical significance (*P* > 0.05) for the higher accuracy of GPT-4V for the clinical and ancillary testing categories: *P* = 0.291 for clinical (*n* = 61) and *P* = 0.153 for ancillary testing (*n* = 76) according to the consensus, suggesting that there may not be a meaningful difference in the performances for these categories between PathChat and the runner-up GPT-4V. Similarly, according to the more subjective ranking-based evaluation, we found that PathChat was comparable to and in fact slightly more preferred by the panel of pathologists compared to GPT-4V (a median win rate of 44.1% and lose rate of 33.8% versus GPT-4V for clinical and a median win rate of 44.8% and lose rate of 35.6% for ancillary testing) on these same categories.

Note that we included clinical and ancillary testing questions to comprehensively assess the capabilities of AI assistant models to address pathology-related queries. However, these questions frequently do not require an actual examination of the histology image but instead mostly aim to test the model’s ability to recall background knowledge relevant to pathology (for example, ‘What specific molecular alterations are commonly found in disease X, and how might they influence the prognosis or therapeutic options?’). As a result, it is not too surprising that even general-purpose multimodal AI assistants such as LLaVA 1.5 can often adequately answer questions in these categories and that GPT-4V may, in particular, excel here, as it is presumably much larger and was trained on more extensive knowledge from the internet than open-source models and PathChat. As these queries can often readily be addressed through conventional means of querying, such as internet searches or consulting a reference manual, we focused on the microscopy and diagnosis categories as the main indicators for the utility of different models as vision-language assistants for pathology, given that for the other two categories, AI assistance is not necessarily required to answer visual questions based on pathology images. A further breakdown of model performance by subcategory is included in Supplementary Tables 28–38. Note that, even though our benchmark for answering open-ended questions is specific to pathology, its size is around double the 140 questions used in an earlier work^58^ in which human experts evaluated the ability of LLMs to encode general clinical knowledge.

Lastly, note that like our observation in the multiple-choice evaluation, of the 260 questions submitted to it, GPT-4V obviously refused to answer 38, presumably because of guardrails implemented within it. A maximum of three attempts were made for each question (see ‘Evaluating GPT-4V’ in Methods for more details). Consistent with our assessment of the other models, all GPT-4V responses, regardless of whether they were successful or not, were blinded, shuffled and presented to pathologists for evaluation without special treatment. However, for transparency, we recorded the number of ultimately unsuccessful queries for GPT-4V in each question category (Supplementary Table 39) and report performance on only the subset of questions that GPT-4V successfully answered (Supplementary Tables 40–64), which saw PathChat still outperforming GPT-4V by a margin of over 20% in accuracy (for example, 81.2% versus 59.9% for 202 questions with consensus, *P* *<* 0.001).

## Demonstration of PathChat on diverse use cases

**Fig. 4:**
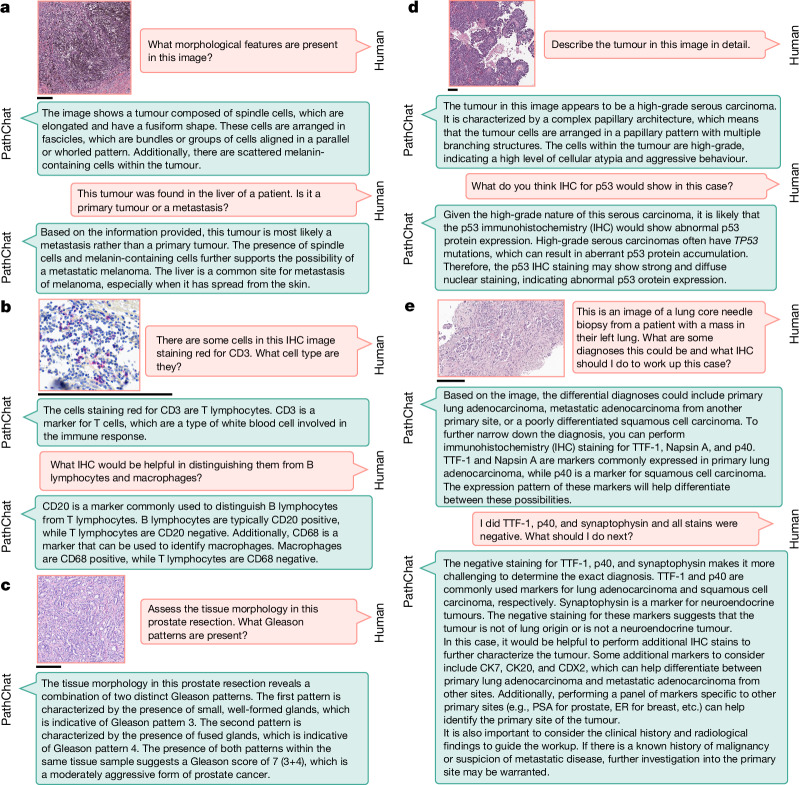
Exploring use cases of PathChat. **a**–**e**, Beyond evaluating PathChat on answering multiple-choice and single-turn open-ended questions, we explored other use cases. The panels contain examples that involve a follow-up from users in the form of interactive, multi-turn conversations. These examples are illustrative in nature and intended to complement our quantitative evaluation of PathQABench. **a**, PathChat summarized key morphological features in an histology image. Based on the clinical context, it could reasonably infer the primary origin of the tumour. **b**, PathChat is familiar with different cell markers and can potentially help by guiding IHC interpretations. **c**, PathChat understands and can attempt to follow well-known guidelines on tumour grading, in this case, the Gleason grading system for prostate adenocarcinoma. **d**, PathChat can describe tumour tissue and cell morphology, infer a diagnosis and correctly suggest potential IHC findings grounded in relevant background knowledge about the suspected malignancy. **e**, PathChat can potentially be consulted to perform human-in-the-loop differential diagnosis that may require several rounds of an IHC workup. Scale bars, 200 µm.

**Extended Data Fig. 10:**
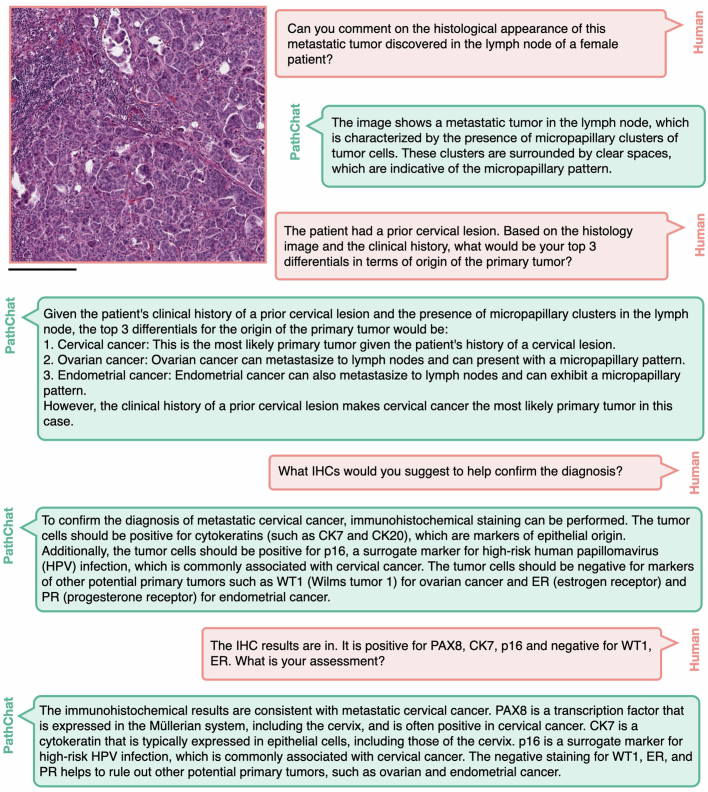
Example of human-in-the-loop differential diagnosis with PathChat in a case of cancer of unknown primary. PathChat can potentially be used to help the user perform human-in-the-loop differential diagnosis that combines a representative histology image, relevant clinical context, as well as follow-up IHC results. Note that in this example, PathChat erroneously implies that cervical cancers should be positive for CK7 and CK20 IHC when in fact, cervical cancers are usually positive for CK7 but negative for CK20. Scale bar is 200 µm.

In addition to evaluating PathChat on answering multiple-choice and open-ended questions, we attempted to conceptualize and explore potential use cases for PathChat as an AI copilot specialized for pathology. We observed that PathChat can analyse and describe notable morphological details in histology images and that it can answer questions that require background knowledge in pathology and general biomedicine in addition to the visual input (Fig. 4a–d). The ability to analyse visual features, flexibly combine them with clinical context and medical knowledge (simply specified with natural language) and then interpret them in the context of specific diagnostic guidelines (for example, Gleason grading) out of the box opens PathChat to a much wider range of applications compared to task-specific visual-question answering or image captioning models fine-tuned on relatively small datasets with a limited scope^39–43^. Additionally, the support for interactive multi-turn conversations enables PathChat to potentially serve as a consultant for human-in-the-loop differential diagnosis, in which an initial AI-assisted assessment can be followed up with further clinical context, namely workups that are subsequently performed to narrow the differential (Fig. 4e and Extended Data Fig. 10). This may be especially valuable in cases that involve more lengthy, complex workups, such as cancers of unknown primary and in low-resource settings where access to experienced pathologists may be limited.

## Discussion

Computational pathology has witnessed substantial progress over the years, such as the development of increasingly accurate, task-specific predictive models based on image or genomics data. For histology images specifically, there has recently been growing interest in building foundational task-agnostic vision encoders pretrained with large numbers of unlabelled images, which can provide robust feature embeddings for diverse supervised and unsupervised downstream workflows. However, the explosive growth in generative AI technology and specifically MLLMs, as exemplified by the likes of ChatGPT, has begun to open up a possible new frontier for both computational pathology research and real-world applications to clinical pathology. Generalist AI models equipped with an understanding of natural language could utilize text as a unified medium both for the flexible specification of user intent (in the form of a tailored prompt) and for producing outputs of various levels of expressiveness (from single-word to binary or multiple-choice responses to coherent sentences with reasoning steps) while performing diverse tasks (for example, summarization, classification, captioning, retrieval, answering questions and more). For pathology specifically, such a model could, in theory, have applications in a wide range of scenarios across education and research as well as human-in-the-loop clinical decision-making.

In this work, we provide a proof of concept for building an AI copilot tailored to human pathology. We also provide, to the best of our knowledge, the most extensive evaluation of such technology for computational pathology by comparing our model, PathChat, both to publicly available models developed for general machine learning and the larger domain of biomedical sciences and to a SOTA commercial solution, GPT-4V. We created PathQABench, a high-quality expert-curated benchmark that aims to assess a diverse range of capabilities relevant to anatomic pathology, including morphological examination of histology microscopy images, making diagnoses based on both histology and clinical context, assessment of tumour grade and differentiation, suggesting further IHC and molecular testing, and understanding the risk factors, prognosis and treatment of the underlying disease. We assessed these skills through a combination of multiple-choice diagnostic questions and open-ended questions coupled with human expert evaluation. In both evaluation settings, PathChat compared favourably to the current best-in-class commercial solution GPT-4V (presumably much larger and expensive to serve than PathChat) and substantially outperformed the publicly available MLLMs tested in terms of diagnostic accuracy and quality of response. Additionally, we demonstrated that the support for interactive multi-turn conversations may enable PathChat to handle other use cases, such as complex diagnostic workups. Considering our findings, we hope PathChat can potentially find impactful applications in pathology education, research and human-in-the-loop clinical decision-making as the technology matures over time.

Further alignment with human intent using techniques such as reinforcement learning from human feedback^44^ may lower hallucination in MLLM-based AI assistant models in general and also help them to capture certain nuances specific to pathology, such as when to request further contextual information or test results when it is not possible or is difficult to rule out certain morphologically similar diseases based on H&E histology alone or when to seek clarification on institutional-specific guidelines for diagnosis and treatment. For real-world deployment, improvement and validation are probably also warranted to ensure that the model can consistently and correctly identify invalid queries (for example, non-pathology-related or nonsensical inputs) and refrain from answering with unexpected or erroneous outputs.

Future research will probably further enhance the capabilities of PathChat and MLLM-based AI assistants by adding support for inputting an entire gigapixel WSI or several WSIs. This may extend their usefulness in the diagnosis of challenging and borderline entities by supplying valuable context beyond preselected representative ROIs. Additionally, owing to their having been trained on retrospectively collected large datasets that inevitably contain outdated information, these models may reflect the scientific consensus of the past rather than that of today^58^. For example, as medical terminology and guidelines evolve, a model response that references the outdated term ‘glioblastoma multiforme’ may result in factual inaccuracies. Besides continual training with fresh, up-to-date knowledge^59^, other research directions may involve curating specific instructions that make the model aware of changes in terminology and guidelines or using retrieval augmented generation^60^ with a continuously updated knowledge database. Lastly, to make these tools more useful to pathologists and researchers, it could be worthwhile to consider explicitly supporting specialized tasks such as precise counting or localization of objects (for example, ‘How many lymphocytes are in this image?’ or ‘Provide the exact bounding box coordinates of mitotic figures’) and integrating PathChat-like AI assistants with tools such as digital slide viewers or electronic health records.

## Methods

### Curation of the PathChat dataset

We curated a dataset of 456,916 instructions with 999,202 question and answer turns, which was used to train PathChat to respond to pathology-specific queries. The instructions were roughly categorized as conversation (*n* = 132,563), description (*n* = 168,440), multiple choice (*n* = 42,445), free response (*n* = 21,686), text-only (*n* = 83,232) and guardrail (*n* = 8,550). An illustrative example of each category is shown in Extended Data Fig. 1. No sample size calculations were performed and all data were de-identified before analysis. To ensure that PathChat could generalize to a diverse range of instructions, the data encompassed several different instruction formats, including open-ended multi-turn dialogues, detailed image descriptions, short-answer questions, multiple-choice questions and text-only questions. A diverse set of data sources were used to generate the instruction dataset, which spanned image captions, educational articles from PubMed Open Access, pathology case reports and ROIs extracted from WSIs, which were sourced from several institutions. Data from TCGA were not used for training and were held out as part of our downstream evaluation. The data for each source were filtered individually to ensure quality and relevance for training a pathology-specific vision-language assistant. Examples of frequently used heuristics for filtering include the removal of image captions that are overly short (for example, less than 12 words) or uninformative and overly generic (for example, ‘An H&E image of a tumour’). We also removed captions or passages related to animal pathology (keywords include ‘rat’ and ‘pig’) and experimental studies (keywords include ‘experimental’ and ‘positive control’) using regex pattern matching. Lastly, we included basic guardrail instruction examples, so that when a model is given image-specific instructions such as ‘Describe this histology image of a lung mass’ but no image is provided, the model is expected to output the response: ‘Sorry, I cannot assist you since you have not uploaded any image.’ Additionally, when given an image not related to pathology (sampled from MS COCO; ref. ^61^), the model is trained to output: ‘Sorry I can only assist you with queries related to pathology.’ For some unstructured data formats, we prompted the open-source general-purpose LLMs^46,62^ to structure the original source text into a structured format automatically. Instructions were then manually created from the structured data with continual input from several board-certified pathologists.

### Design and training of the PathChat model

Compared to text-only LLMs, an MLLM is trained to understand and respond to user instructions in the form of natural language queries that may additionally contain inputs from other modalities such as images. Support for multimodality is essential for histopathology, as examining and interpreting visual information in high-resolution microscopy images (in conjunction with other clinical information) remains the cornerstone of the discipline and extends to many aspects of disease diagnosis and management in modern medicine.

Inspired by LLaVA^5,63^, our MLLM, PathChat, consists of three key components: the vision encoder, the multimodal projector module and the LLM. The vision encoder is responsible for encoding an image from the original high-dimensional RGB pixel space into a low-dimensional feature representation suitable for processing by the downstream modules. The multimodal projector connects the outputs of the vision encoder to the LLM by projecting the visual tokens to the same dimension as the LLM’s embedding space for text tokens. The LLM takes a natural language instruction as input (after it has been tokenized by a tokenizer), combines the embedded text tokens and the image token output from the multimodal projector to form the full sequence of input tokens, and predicts the desirable response through autoregressive next-word prediction. The response produced is finally decoded by the tokenizer back into natural language and presented to the end user.

For the LLM, we adopted the 13-billion-parameter variant from the widely used Meta Llama 2 family^46^ of SOTA open-source LLMs, which is a decoder-only transformer-based autoregressive language model with 40 transformer layers, each with 40 attention heads, an embedding dimension of 5,120 and a hidden dimension of 13,824. It uses rotary positional encodings and natively supports a maximum context length of 4,096. As with LLaVA 1.5, we used a vision encoder based on the standard ViT-Large architecture consisting of 24 transformer multi-headed attention blocks, each with 16 attention heads, an embedding dimension of 1,024 and a feedforward hidden dimension of 4,096. The token size was 16 × 16, and we added learned absolute positional encoding to each token. The multimodal projector consists of an attention pooling layer followed by a two-layer multilayer perceptron. The attention pooling layer (also known as a perceiver resampler in some works^49,64,65^) uses a set of 128 learned latent queries and multi-headed cross-attention with 8 heads to reduce the last layer feature map of the encoder backbone into a fixed-length sequence of image tokens with an initial dimension of 768 to increase training and inference efficiency and to prevent the total sequence length of tokens from potentially exceeding the context window size of the LLM. The subsequent multilayer perceptron follows the design used in LLaVA 1.5 and consists of a single hidden layer and an activation function based on Gaussian error linear units. It projects the image tokens up to the embedding dimension of the LLM (5,120 for the Llama 2 13B model). We initialized the weights of the vision-encoder backbone from UNI^3^, a SOTA vision-only self-supervised pretrained general-purpose encoder for H&E pathology and then fine-tuned the encoder backbone together with the attention pooling module on an expanded dataset of 1.18 paired images and captions from CONCH^34^ and the CoCa visual-language pretraining recipe^66^ (see Supplementary Table 1 for details of the hyperparameters).

We followed the MLLM training recipe of LLaVA 1.5, which involves two stages of training. In the first, pretraining stage, the LLM weights are kept frozen and only the multimodal projector receives parameter updates to learn a suitable projection from the space of image tokens to the shared embedding space of the text tokens used by the LLM. For this simple purpose, the MLLM is supervised and simply predicts the caption corresponding to each image using roughly 100,000 image and caption pairs sampled from our previous dataset^34^, without using any curated instruction data. In the second stage, the instruction fine-tuning stage, both the LLM and projector are trained end-to-end to generate responses to diverse instructions that include both natural language and visual inputs, as described in ‘PathChat dataset curation’. Specifically, given an instruction **X**_instruct_, the reference answer **X**_ans_ and the image **X**_img_, each represented as a sequence of tokenized inputs, we maximized the likelihood of each token in **X**_ans_, indexed by 1*, ..., L*, under the MLLM (viewed as an autoregressive language model):

$${L}_{{\rm{c}}{\rm{l}}{\rm{m}}}({\theta }_{{\rm{p}}{\rm{r}}{\rm{o}}{\rm{j}}{\rm{e}}{\rm{c}}{\rm{t}}{\rm{o}}{\rm{r}}},{\theta }_{{\rm{l}}{\rm{l}}{\rm{m}}})=\mathop{\sum }\limits_{i=1}^{L}\log p({{\bf{X}}}_{{\rm{a}}{\rm{n}}{\rm{s}},i}|{{\bf{X}}}_{{\rm{a}}{\rm{n}}{\rm{s}},1:i-1},{{\bf{X}}}_{{\rm{i}}{\rm{n}}{\rm{s}}{\rm{t}}{\rm{r}}{\rm{u}}{\rm{c}}{\rm{t}}},{{\bf{X}}}_{{\rm{i}}{\rm{m}}{\rm{g}}};{\theta }_{{\rm{p}}{\rm{r}}{\rm{o}}{\rm{j}}{\rm{e}}{\rm{c}}{\rm{t}}{\rm{o}}{\rm{r}}},{\theta }_{{\rm{l}}{\rm{l}}{\rm{m}}}).$$

This instruction tuning objective easily extends to multi-turn instruction data by conditioning on all previous turns of instructions and reference answers. For instructions with no image, **X**_img_ is not defined and is removed from the conditioning sequence. Similarly, if several images accompany a given instruction, we simply concatenate their respective image tokens, with the newline (‘\n’) token inserted between them as a separator, and treat the full sequence as **X**_img_. Both pretraining and fine-tuning were performed using eight 80 GB NVIDIA A100 GPUs. We refer readers to Supplementary Tables 2 and 3 for details of the hyperparameters used in training.

### Benchmark for expert-curated pathology questions

Evaluating powerful multimodal vision-language AI models in histopathology is an outstanding challenge, and, to the best of our knowledge, there is at present no publicly available high-quality expert-curated histopathology-centric quality-assessment benchmark. One possible candidate is PathVQA^43^, which has been used in the literature to demonstrate and evaluate the capabilities of various AI models in understanding pathology images. However, our manual audit revealed numerous types of low-quality examples in the benchmark, probably due to the lack of expert review and the automated nature of the data curation workflow used by PathVQA. Thus motivated, we curated a new high-quality quality-assessment benchmark suitable for evaluating cutting-edge MLLMs for pathology, as described in detail below.

To evaluate PathChat, we curated PathQABench using representative high-resolution ROI images hand-selected by a board-certified pathologist from 105 H&E WSI cases using the open-source QuPath digital viewer^67^. These cases were withheld from all stages of training PathChat. Of the 105 image ROIs, 53 ROIs were curated from private sources in-house at the Brigham & Women’s Hospital for the study, whereas the other 52 ROIs were selected from WSIs in the public TCGA repository. The WSIs cover 11 tissue sites and 54 diagnoses (Supplementary Tables 5 and 6). This design choice enabled us to use the subset of questions based on publicly available WSIs, referred to as PathQABench-Public, to evaluate the SOTA commercial solution GPT-4V (powering ChatGPT-4 with vision capabilities) through API requests, without any risk of violating institutional guidelines for handling patient data. Accordingly, the subset of questions based on private WSIs, referred to as PathQABench-Private, was used to evaluate only other publicly available MLLM solutions that we can run locally inside the hospital without transmitting the data to an external server. To select the ROIs, a board-certified pathologist manually reviewed WSIs related to each diagnosis and distilled a single ROI for each WSI wherein relevant morphologic features of the diagnosis were evident. The diagnosis from these WSIs was then transferred to that of the image ROIs and subsequently used in the evaluation, both for open-ended and multiple-choice questions. These diagnoses were originally made by separate pathologists who had examined the cases clinically. They had full access to any other slides in the case and the patient’s medical record and were able to order and interpret IHC tests as required to work up the case. To accommodate the diversity of diagnoses included in our evaluation, the selected ROIs vary in magnification and dimension. Across PathQABench, the selected magnifications of the ROIs ranged from ×3 to ×34.4 with a median of ×13.3. The widths varied from 859 to 2,388 px with a median of 1,201 px whereas the heights varied from 861 to 2,390 px with a median of 1,191 px. For each case, the pathologist wrote a short clinical summary based on the ground truth diagnosis, which included appropriately devised patient age, sex and clinical symptoms and radiology findings where applicable. This summary is referred to as the clinical context of the case. An example of clinical context is shown in Fig. 2a. We then created both close-ended multiple-choice diagnostic questions and open-ended questions that aimed to assess each model’s capability in assisting with diverse pathology-related queries, which cover a range of topics that include but are not limited to just diagnosis (Extended Data Fig. 7 and Supplementary Table 15).

A total of 105 multiple-choice questions were created using the salient ROIs (one question per ROI). In the evaluation setting with multiple-choice questions, for each organ system, a board-certified pathologist selected a set of ten possible answers that encompassed the correct answers for all questions within that organ system as well as other relatively common diagnoses within that organ system (Supplementary Table 7). For each multiple-choice question, we considered two evaluation strategies. In the first image-only setting, the model was presented with only the image and the multiple-choice question as input. In the second, image + clinical context setting, which was designed to more closely mimic a real-world diagnostic workflow, the clinical context was additionally provided together with the histology image. In both settings, a model was assessed based on its ability to accurately select the ground truth diagnosis from the set of possible options.

In the evaluation setting for answering open-ended questions, we used the 52 cases from PathQABench-Public to curate five questions per case for a total of 260 questions. The questions were broadly categorized as microscopy, diagnosis, clinical and ancillary testing, as described in Supplementary Table 15. The microscopy and diagnosis questions, in particular, focus on targeting diagnosis and morphological examination using the histology images and other relevant context (where applicable), which are essential skills in anatomic pathology. On the other hand, the clinical and ancillary testing categories contain text-only questions that do not require the visual examination of an image to answer, as they cover topics such as how to use IHC to confirm a diagnosis and background knowledge pertaining to the underlying condition. Note that, although our benchmark for answering open-ended questions is specific to pathology, its size is substantially larger than the 140 questions used in an earlier work^58^ in which human experts evaluated the ability of LLMs to encode general clinical knowledge.

### MLLM evaluation

We compared PathChat to the general-purpose SOTA MLLM LLaVA 1.5 (ref. ^63^) and to the biomedically focused MLLM LLaVA-Med^53^ using the full PathQABench dataset. We evaluated the performance of GPT-4V only on cases from PathQABench-Public. The precise pretrained checkpoints for these models are specified in ‘Code availability’ and Reporting summary. We used the default image processor implemented by each model and used greedy decoding during inference when possible (which is not presently supported by the GPT-4V API, so, instead, we used the default arguments set by OpenAI). The evaluation of GPT-4V also required a more involved protocol because of the guardrails implemented by OpenAI, which we detail in the next section (‘Evaluating GPT-4V’). For all models, the maximum length of each generated response was capped to 1,024 new tokens generated.

For the multiple-choice questions, we observed that PathChat, LLaVA 1.5 and GPT-4V can output the predicted choice in a consistent and desirable format (for example, ‘A’ or ‘A. Lung adenocarcinoma’), which can be directly used in our evaluation pipeline to compute the accuracy. However, we found LLaVA-Med could not follow the instruction to answer in a concise and consistent format appropriate for multiple-choice questions and instead would always output a full sentence. Therefore, for LLaVA-Med, a board-certified pathologist first manually reviewed each model response, extracted the predicted diagnosis, assessed its correctness against the ground truth and then computed the accuracy.

For the open-ended questions, we gathered the predictions for each model and presented them to a panel of seven pathologists, who evaluated them by ranking them based on their human expertise. For each question, the order of the model responses was randomly shuffled and the pathologist was blinded as to which model produced which response. The responses were ranked based on, in order of importance: (1) following the prompt (whether the response correctly addressed the instruction), (2) completeness of the answer, (3) succinctness and (4) use of accepted pathology terminology. Ties of two (or more) responses were allowed. This part of the evaluation aimed to capture a wide range of expert judgement (including subjective human preference) on the responses.

Additionally, we attempted to assign a more objective binary correct versus incorrect outcome for each response. For this task, we first asked two board-certified pathologists to independently assess each response to each question (in terms of correct versus incorrect for each model). Both pathologists were blinded to which model produced which response. For questions with a single best answer (for example, ‘What is the most likely diagnosis?’), the responses were labelled as incorrect if the single best answer was not provided. For the open-ended questions (for example, ‘What IHC stains would be useful in working up a glioblastoma?’), responses were labelled as incorrect if any portion of the response was hallucinated or if the response did not answer the question at all. Correct and incorrect labels were mutually exclusive and every response was labelled as correct or incorrect. Overall, across all models and all questions, the two experts agreed 92.6% of the time in their assessment with a corresponding Cohen’s kappa score of 0.852, indicating substantial interobserver agreement, which was expected given the more objective nature of this part of the evaluation. To establish a consensus, we asked the two experts to discuss their assessments for the questions on which they disagreed originally. Following this discussion, they ultimately agreed completely on 235 of the 260 questions for all models. In the ‘Performance on answering open-ended questions’ section, we report the performance on this subset of questions where a consensus was reached (using the consensus as the ground truth) and report the performance according to each individual expert’s assessment for all questions in Extended Data Fig. 6.

### Evaluating GPT-4V

GPT-4V was evaluated using the official API provided by OpenAI. All API calls were made during February 2024 for gpt-4-vision-preview (the default, most up-to-date vision-enabled GPT-4 model available at the time of the study). We observed that guardrails appear to have been implemented into GPT-4V to prevent it from addressing queries that require an examination of histopathology images. In such instances, it may inform the user that it cannot provide an interpretation of the pathology image and that they should instead consult a trained medical professional. Queries for which GPT-4V obviously refused to address the given instructions were deemed ‘unsuccessful’. In such instances, we made a maximum of two further resubmissions for the same query for up to a total of three attempts. Following this evaluation protocol, we recorded 28 out of 52 successful queries in the multiple-choice diagnostic assessment of PathQABench-Public cases when no further clinical context was provided with a question. By contrast, 47 out of 52 queries were eventually successful when the clinical context was included. Using an analogous protocol, in the open-ended quality assessment with PathQABench-Public, we counted 222 out of 260 successful queries. All final responses, regardless of whether they were successful or unsuccessful, were presented to the pathologists for evaluation without special treatment and subjected to the same blinding and shuffling protocol used for the other models (‘MLLM evaluation’). A breakdown of successful queries by category is provided in Supplementary Table 39.

### Statistical analysis

We used nonparametric bootstrapping (*n* = 1,000 replicates) to estimate 95% confidence intervals for the reported metrics. Observed differences in performance for a pair of models were tested for statistical significance using a two-sided paired permutation test (*n* = 1,000 permutations), with the null hypothesis being that there is no difference in the performance of the two models. In each permutation, independent pairs of prediction outcomes for the two models were randomly swapped to obtain a new difference in model performance. The *P* value corresponds to the proportion of differences in model performance with a greater absolute value than the observed difference.

### Computing hardware and software

We used Python (v.3.10.13) for all experiments and analyses in the study. For all model training, we used eight 80 GB NVIDIA A100 GPUs configured for multi-GPU training using the popular open-source deep learning framework PyTorch (v.2.0.1, CUDA 11.8). All inference jobs were performed using 24 GB NVIDIA 3090 GPUs. We used the implementation of MLLM training and inference provided by LLaVA (v.1.1.3) and incorporated our own custom vision encoder and multimodal projector implemented in Timm (v.0.9.2) and PyTorch. Pillow (v.10.1.0) was used for image processing. Flash Attention (v.2.3.3) and DeepSpeed (v.0.9.5) were used to enable accelerated training of PathChat MLLM. For illustration and evaluation, we used images from PathQABench and other real-world cases not used for model training. Matplotlib (v.3.7.1) and Seaborn (v.0.12.2) were used to create plots and figures. Other miscellaneous libraries used are listed in the Reporting summary. UNI, a pretrained foundational vision encoder, was trained for 32 h on 32 80 GB NVIDIA A100 GPUs in a four-node distributed set-up (eight GPUs per node). The vision encoder used in PathChat was fine-tuned from UNI using a single node of eight 80 GB NVIDIA A100 GPUs for 21.5 h. Lastly, the combined system of PathChat (including the vision encoder, the multimodal projector and the LLM) were jointly trained for a total of 17 h and 18 min (includes both pretraining and fine-tuning) on a single node of eight 80 GB NVIDIA A100 GPUs to produce the final model. For inference, the PathChat model was run on two 24 GB NVIDIA RTX 3090 GPUs, which yielded an average time of 9.75 s (standard deviation of 7.71 s) per response on the 260 open-ended questions.

### Ethics approval

The Mass General Brigham institutional review board approved the retrospective analysis of pathology slides and corresponding pathology reports. All pathology images were de-identified before computational analysis and model development.

### Reporting summary

Further information on research design is available in the Nature Portfolio Reporting Summary linked to this article.

## Online content

Any methods, additional references, Nature Portfolio reporting summaries, source data, extended data, supplementary information, acknowledgements, peer review information; details of author contributions and competing interests; and statements of data and code availability are available at 10.1038/s41586-024-07618-3.

## Supplementary information

Supplementary Information Supplementary Tables 1–64.

Reporting Summary

## Data Availability

The PubMed Central-OA dataset can be accessed from the National Institutes of Health (NIH) PubMed Central website (https://www.ncbi.nlm.nih.gov/pmc/tools/openftlist/). The TCGA WSIs and associated clinical metadata are available from the NIH genomic data commons (https://portal.gdc.cancer.gov). The curated PathQABench-Public benchmark is released for research use and can be accessed through: https://github.com/fedshyvana/pathology_mllm_training. Patient data used in this project were curated with institutional permission through approval by the institutional review board for the current study and, thus, cannot be made publicly available in compliance with patient privacy obligations. All requests for processed data curated internally will be evaluated based on institutional and departmental policies to determine whether the data requested are subject to intellectual property or patient privacy obligations. Data that can be transferred will require a material or data transfer agreement between the institutions and will limit the utility of the data to non-commercial academic research purposes. The exact timeline will depend on the execution of such agreements. Please email all requests to the corresponding author (and also include M.Y.L., mlu16@bwh.harvard.edu).

## Code availability

The code used to train PathChat has been made publicly available for non-commercial academic use and can be accessed here: https://github.com/fedshyvana/pathology_mllm_training (ref. ^68^). We have documented all technical deep learning methods and software libraries used in the study, with hyperparameters detailed in Supplementary Tables 1–3. Pathology MLLM weights trained on proprietary internal patient text data are subject to patient privacy and intellectual property obligations and cannot be made available. The code for LLaVA can be accessed here: https://github.com/haotian-liu/LLaVA. The code for LLaVA-Med can be accessed here: https://github.com/microsoft/LLaVA-Med.

## References

[1] Song, A. H. et al. Artificial intelligence for digital and computational pathology. *Nat. Rev. Bioeng.* **1**, 930–949 (2023).

[2] Shmatko, A. et al. Artificial intelligence in histopathology: enhancing cancer research and clinical oncology. *Nat. Cancer* **3**, 1026–1038 (2022). 36138135 10.1038/s43018-022-00436-4

[3] Chen, R. J et al. Towards a general-purpose foundation model for computational pathology. *Nat. Med.* **30**, 850–862 (2024). 10.1038/s41591-024-02857-3 PMC11403354 38504018

[4] Ciga, O., Xu T. & Martel A. L. Self supervised contrastive learning for digital histopathology. *Mach. Learn. Appl.* **7**, 100198 (2022).

[5] Liu, H. et al. Visual instruction tuning. In *Proc. Advances in Neural Information Processing Systems* (eds Oh, A. et al.) 34892–34916 (Curran Associates, 2023).

[6] Josh, A. et al. GPT-4 technical report. Preprint at arxiv.org/abs/2303.08774 (2023).

[7] Lipkova, J. et al. Artificial intelligence for multimodal data integration in oncology. *Cancer Cell* **40**, 1095–1110 (2022). 36220072 10.1016/j.ccell.2022.09.012 PMC10655164

[8] Coudray, N. et al. Classification and mutation prediction from non–small cell lung cancer histopathology images using deep learning. *Nat. Med.* **24**, 1559–1567 (2018). 30224757 10.1038/s41591-018-0177-5 PMC9847512

[9] Lu, M. Y. et al. Data-efficient and weakly supervised computational pathology on whole-slide images. *Nat. Biomed. Eng.* **5**, 555–570 (2021). 33649564 10.1038/s41551-020-00682-w PMC8711640

[10] Bulten, W. et al. Automated deep-learning system for Gleason grading of prostate cancer using biopsies: a diagnostic study. *Lancet Oncol.* **21**, 233–241 (2020). 31926805 10.1016/S1470-2045(19)30739-9

[11] Bulten, W. et al. Artificial intelligence for diagnosis and Gleason grading of prostate cancer: the PANDA challenge. *Nat. Med.* **28**, 154–163 (2022). 35027755 10.1038/s41591-021-01620-2 PMC8799467

[12] Ehteshami Bejnordi, B. et al. Diagnostic assessment of deep learning algorithms for detection of lymph node metastases in women with breast cancer. *J. Am. Med. Assoc.* **318**, 2199–2210 (2017). 10.1001/jama.2017.14585 PMC5820737 29234806

[13] Beck, A. H. et al. Systematic analysis of breast cancer morphology uncovers stromal features associated with survival. *Sci. Transl. Med.* **3**, 108ra113 (2011). 22072638 10.1126/scitranslmed.3002564

[14] Chen, R. J. et al. Pan-cancer integrative histology-genomic analysis via multimodal deep learning. *Cancer Cell* **40**, 865–878 (2022). 35944502 10.1016/j.ccell.2022.07.004 PMC10397370

[15] Lee, Y. et al. Derivation of prognostic contextual histopathological features from whole-slide images of tumours via graph deep learning. *Nat. Biomed. Eng.*10.1038/s41551-022-00923-0 (2022). 10.1038/s41551-022-00923-0 35982331

[16] Amgad, M. et al. A population-level digital histologic biomarker for enhanced prognosis of invasive breast cancer. *Nat. Med.* **30**, 85–97 (2024). 10.1038/s41591-023-02643-7 38012314

[17] Mobadersany, P. et al. Predicting cancer outcomes from histology and genomics using convolutional networks. *Proc. Natl Acad. Sci. USA* **115**, E2970–E2979 (2018). 29531073 10.1073/pnas.1717139115 PMC5879673

[18] Sammut, S.-J. et al. Multi-omic machine learning predictor of breast cancer therapy response. *Nature* **601**, 623–629 (2022). 34875674 10.1038/s41586-021-04278-5 PMC8791834

[19] Huang, Z. et al. Artificial intelligence reveals features associated with breast cancer neoadjuvant chemotherapy responses from multi-stain histopathologic images. *npj Precis. Oncol.* **7**, 14 (2023). 36707660 10.1038/s41698-023-00352-5 PMC9883475

[20] Lu, M. Y. et al. AI-based pathology predicts origins for cancers of unknown primary. *Nature* **594**, 106–110 (2021). 33953404 10.1038/s41586-021-03512-4

[21] Tian, F. et al. Prediction of tumor origin in cancers of unknown primary origin with cytology-based deep learning. *Nat. Med.* **30**, 1309–1319 (2024). 10.1038/s41591-024-02915-w PMC11108774 38627559

[22] Kather, J. N. et al. Pan-cancer image-based detection of clinically actionable genetic alterations. *Nat. Cancer* **1**, 789–799 (2020). 33763651 10.1038/s43018-020-0087-6 PMC7610412

[23] Fu, Y. et al. Pan-cancer computational histopathology reveals mutations, tumor composition and prognosis. *Nat. Cancer* **1**, 800–810 (2020). 35122049 10.1038/s43018-020-0085-8

[24] Wagner, S. J. et al. Transformer-based biomarker prediction from colorectal cancer histology: a large-scale multicentric study. *Cancer Cell* **41**, 1650–1661 (2023). 37652006 10.1016/j.ccell.2023.08.002 PMC10507381

[25] Graham, S. et al. One model is all you need: multi-task learning enables simultaneous histology image segmentation and classification. *Med. Image Anal.* **83**, 102685 (2023). 36410209 10.1016/j.media.2022.102685

[26] Oquab, M. et al. DINOv2: learning robust visual features without supervision. *Trans. Machine Learning Res.*, 1–31 (2024).

[27] Radford, A. et al. Learning transferable visual models from natural language supervision. In *Proc. International Conference on Machine Learning* (eds Meila, M. & Zhang, T.) 8748–8763 (PMLR, 2021).

[28] Lu, J. et al. ViLBERT: pretraining task-agnostic visiolinguistic representations for vision-and-language tasks. In *Proc. Advances in Neural Information Processing Systems* (eds Wallach, H. et al.) (Curran Associates, 2019).

[29] Schaumberg, A. J. et al. Interpretable multimodal deep learning for real-time pan-tissue pan-disease pathology search on social media. *Mod. Pathol.* **33**, 2169–2185 (2020). 32467650 10.1038/s41379-020-0540-1 PMC7581495

[30] Huang, Z. et al. A visual–language foundation model for pathology image analysis using medical Twitter. *Nat. Med.* **29**, 2307–2316 (2023). 37592105 10.1038/s41591-023-02504-3

[31] Zhang, S. et al. BiomedCLIP: a multimodal biomedical foundation model pretrained from fifteen million scientific image-text pairs. Preprint at arxiv.org/abs/2303.00915 (2023).

[32] Gamper, J. & Rajpoot, N. Multiple instance captioning: learning representations from histopathology textbooks and articles. In *Proc. IEEE/CVF Conference on Computer Vision and Pattern Recognition* 16549–16559 (IEEE, 2021).

[33] Ikezogwo, W. et al. Quilt-1m: one million image-text pairs for histopathology. In *Proc. Advances in Neural Information Processing Systems* (eds Oh, A. et al.) 37995–38017 (Curran Associates, 2024). PMC11090501 38742142

[34] Lu, M. Y. et al. A visual-language foundation model for computational pathology. *Nat. Med.* **30**, 863–874 (2024). 38504017 10.1038/s41591-024-02856-4 PMC11384335

[35] Lu, M. Y. et al. Visual language pretrained multiple instance zero-shot transfer for histopathology images. In *Proc. IEEE/CVF Conference on Computer Vision and Pattern Recognition* 19764–19775 (IEEE, 2023).

[36] Tiu, E. et al. Expert-level detection of pathologies from unannotated chest X-ray images via self-supervised learning. *Nat. Biomed. Eng.* **6**, 1399–1406 (2022). 36109605 10.1038/s41551-022-00936-9 PMC9792370

[37] Zhang, Y. et al. Contrastive learning of medical visual representations from paired images and text. In *Proc. Machine Learning for Healthcare Conference* (eds Lipton, Z. et al.) 2–25 (PMLR, 2022).

[38] Boecking, B. et al. Making the most of text semantics to improve biomedical vision–language processing. In *Proc. European Conference on Computer Vision* (eds Avidan, S. et al.) 1–21 (Springer, 2022).

[39] Zhang, H. et al. PathNarratives: data annotation for pathological human–AI collaborative diagnosis. *Front. Med.* **9**, 1070072 (2023). 10.3389/fmed.2022.1070072 PMC9908590 36777158

[40] Tsuneki, M. & Kanavati, F. Inference of captions from histopathological patches. In *Proc. International Conference on Medical Imaging with Deep Learning* (Konukoglu, E. et al.) 1235–1250 (PMLR, 2022).

[41] Zhang, R. et al. Evaluating and interpreting caption prediction for histopathology images. In *Proc. Machine Learning for Healthcare Conference* (eds Doshi-Velez, F. et al.) 418–435 (PMLR, 2020).

[42] Naseem, U., Khushi, M. & Kim, J. Vision-language transformer for interpretable pathology visual question answering. *IEEE J. Biomed. Health Inform.* **27**, 1681–1690 (2022). 10.1109/JBHI.2022.3163751 35358054

[43] He, X. Towards visual question answering on pathology images. In *Proc. 59th Annual Meeting of the Association for Computational Linguistics and the 11th International Joint Conference on Natural Language Processing* (eds Zong, C. et al.) 708–718 (ACL, 2021).

[44] Ouyang, L. et al. Training language models to follow instructions with human feedback. In *Proc. Advances in Neural Information Processing Systems* (eds Koyejo, S. et al.) 27730–27744 (Curran Associates, 2022).

[45] Brown, T. et al. Language models are few-shot learners. In *Proc. Advances in Neural Information Processing Systems* (eds Larochelle, H. et al.) 1877–1901 (Curran Associates, 2020).

[46] Touvron, H. et al. Llama 2: open foundation and fine-tuned chat models. Preprint at arxiv.org/abs/2307.09288 (2023).

[47] Chowdhery, A. et al. Palm: scaling language modeling with pathways. *J. Mach. Learn. Res.* **24**, 1–113 (2023).

[48] Li, C. et al. Multimodal foundation models:: from specialists to general-purpose assistants. *Foundations and Trends® in Computer Graphics and Vision* **16**, 1–214 (2024).

[49] Alayrac, J.-B. et al. Flamingo: a visual language model for few-shot learning. In *Proc.**Advances in Neural Information Processing Systems* (eds Koyejo, S. et al.) 23716–23736 (Curran Associates, 2022).

[50] Moor, M. et al. Foundation models for generalist medical artificial intelligence. *Nature* **616**, 259–265 (2023). 37045921 10.1038/s41586-023-05881-4

[51] Bubeck, S. et al. Sparks of artificial general intelligence: early experiments with GPT-4. Preprint at arxiv.org/abs/2303.12712 (2023).

[52] Sun, Y. et al. PathAsst: a generative foundation AI assistant towards artificial general intelligence of pathology. In *Proc. AAAI Conference on Artificial Intelligence* (eds Wooldridge, M. et al) 5034–5042 (AAAI Press, 2024).

[53] Li, C. et al. LlaVA-Med: training a large language-and-vision assistant for biomedicine in one day. In *Proc. Advances in Neural Information Processing Systems* (eds Oh, A. et al.) 28541–28564 (Curran Associates, 2024).

[54] Tu, T. et al. Towards generalist biomedical AI. *New Engl. J. Med. Artif. Intell.* **1**, AIoa2300138 (2024).

[55] Wu, C. et al. Can GPT-4V (ision) serve medical applications? Case studies on GPT-4V for multimodal medical diagnosis. Preprint at arxiv.org/abs/2310.09909 (2023).

[56] Oon, M. L. et al. Bridging bytes and biopsies: a comparative analysis of ChatGPT and histopathologists in pathology diagnosis and collaborative potential. *Histopathology* **84**, 601–613 (2023). 10.1111/his.15100 38032062

[57] Seyfioglu, M. S. et al. Quilt-LLaVA: visual instruction tuning by extracting localized narratives from open-source histopathology videos.” In *Proc. IEEE/CVF Conf. on Computer Vision and Pattern Recognition*, 13183–13192 (IEEE, 2024).

[58] Singhal, K. et al. Large language models encode clinical knowledge. *Nature* **620**, 172–180 (2023). 37438534 10.1038/s41586-023-06291-2 PMC10396962

[59] Jin, X. et al. Lifelong pretraining: continually adapting language models to emerging corpora. In *Proc. 2022 Conference of the North American Chapter of the Association for Computational Linguistics: Human Language Technologies* (eds Carpuat, M. et al.) 4764–4780 (ACL, 2022).

[60] Lewis, P. et al. Retrieval-augmented generation for knowledge-intensive NLP tasks. In *Proc. Advances in Neural Information Processing Systems* (eds Larochelle, H. et al.) 9459–9474 (Curran Associates, 2020).

[61] Lin, T.-Y. et al. Microsoft COCO: Common objects in context. In *Proc. Computer Vision–ECCV 2014: 13th European Conference* (eds Fleet, D. et al.) 740–755 (Springer, 2014).

[62] Bai, J. et al. Qwen technical report. Preprint at arxiv.org/abs/2309.16609 (2023).

[63] Liu, H. et al. Improved baselines with visual instruction tuning. In *Proc. IEEE/CVF Conf. on Computer Vision and Pattern Recognition*, 26296–26306 (IEEE, 2024).

[64] Zeng, Y. et al. What matters in training a GPT4-style language model with multimodal inputs? In *Proc. 2024 Conf. of the North American Chapter of the Association for Computational Linguistics: Human Language Technologies (Volume 1: Long Papers)*, 7930–7957 (2024).

[65] Jaegle, A. et al. Perceiver: general perception with iterative attention. In *Proc.**International Conference on Machine Learning* (eds Meila, M. & Zhang, T.) 4651–4664 (PMLR, 2021).

[66] Yu, J. et al. CoCa: contrastive captioners are image–text foundation models. *Trans. Mach. Learn. Artif. Intell.* https://openreview.net/forum?id=Ee277P3AYC (2022).

[67] Bankhead, P. et al. QuPath: open source software for digital pathology image analysis. *Sci. Rep.* **7**, 16878 (2017). 29203879 10.1038/s41598-017-17204-5 PMC5715110

[68] Lu, M. Y. et al. Code for pathology MLLM training, version 0.1, April 2024. *GitHub* github.com/fedshyvana/pathology_mllm_training (2024).

