# Supplementary material for: A multimodal generative AI copilot for human pathology
Source: Nature. 2024 Jun 12;634(8033):466–73. doi: 10.1038/s41586-024-07618-3 (PMC11464372; doi:10.1038/s41586-024-07618-3)
Supplement: Supplementary file 1 — Supplementary Tables 1–64. [file 41586_2024_7618_MOESM1_ESM.pdf]

nature portfolio

https://doi.org/10.1038/s41586-024-07618-3

Supplementary information

A multimodal generative AI copilot for human pathology

In the format provided by the
authors and unedited

Hyperparameter	Value
Automatic mixed precision	FP16
Batch size	192
Gradient accumulation steps	4
Learning rate scheduler	Cosine
Warmup steps	250
Peak learning rate	1e-4
AdamW β	(0.9, 0.999)
AdamW ϵ	1e-8
Weight decay	0.2
Softmax temperature	Learned
Epochs	20

Supplementary Data Table 1: Hyperparameters used in visual-language pretraining. *Batch size* refers to the total batch size across GPUs. Effective batch size used for optimization is *batch size* × *gradient accumulation steps*. Learning rate is increased from zero linearly to the *peak learning rate* over the course of *warmup steps* and decays back to zero following the *learning rate scheduler*. The maximum sequence length for captions is set to 128. Non-squared images are first padded to square and then resized to 448 × 448. The same image preprocessor is used in all subsequent stages of model training and evaluation.

Hyperparameter	Value
Automatic mixed precision	BF16
DeepSpeed ZeRO	Stage 3
LLM max. context length	4,096
Batch size	128
Learning rate scheduler	Cosine
Warmup ratio	0.03
Peak learning rate	2e-3
AdamW β	(0.9, 0.999)
AdamW ϵ	1e-8
Weight decay	0.
Gradient clipping max. norm	1.0
Training epochs	1
Gradient checkpointing	Yes
TF32	Yes

Supplementary Data Table 2: Hyperparameters used in PathChat model pretraining. 8 × 80GB NVIDIA A100 GPUs were used for training. *Batch size* refers to the effective batch size (*i.e.*, the total batch size across GPUs). The learning rate is increased from zero linearly to the *peak learning rate* over the course of *total number batches* × *warmup ratio* steps and and decays back to zero following the *learning rate scheduler*.

Hyperparameter	Value
Automatic mixed precision	BF16
DeepSpeed ZeRO	Stage 3
LLM max. context length	4,096
Batch size	64
Gradient accumulation steps	2
Learning rate scheduler	Cosine
Warmup ratio	0.03
Peak learning rate	2e-5
AdamW β	(0.9, 0.999)
AdamW ϵ	1e-8
Weight decay	0.
Gradient clipping max. norm	1.0
Training epochs	1
Gradient checkpointing	Yes
TF32	Yes

Supplementary Data Table 3: Hyperparameters used in PathChat model finetuning. 8 $\times$ 80GB NVIDIA A100 GPUs were used for training. *Batch size* refers to the total batch size across GPUs. The effective batch size used for optimization is *batch size* $\times$ *gradient accumulation steps*. The learning rate is increased from zero linearly to the *peak learning rate* over the course of *total number batches* $\times$ *warmup ratio* steps and decays back to zero following the *learning rate scheduler*.

Major Tissue Type	Num. Slides
Heart	10,427
Lung	9,846
Kidney	8,388
Bowel / Lower GI	8,303
Soft Tissue	7,863
Brain	7,412
Esophagogastric	6,705
Endocrine	6,138
Female Genital Tract	5,796
Lymphatic System	4,957
Liver Biliary Tract	4,677
Male Genital Tract	4,017
Skin	3,653
Breast	3,364
Bone	2,667
Pancreas	2,328
Head & Neck	1,555
Peritoneum	1,210
Bladder	1,059
Eye	61
Major Disease Type	Num. Slides
Neoplasia	54,973
Normal	24,922
Inflammatory / Infectious	15,476
Other	5,055
Total	100,426

Supplementary Data Table 4: Major tissue type and disease Type distribution of self-supervised pre-training. The pretraining dataset for UNI, the foundational vision encoder, consists of 100 million tissue patches from 100,426 whole-slide images (WSIs) sourced from 52,847 cases between 2002 - 2022, spanning routine clinical practice and molecular testing at Massachusetts General Hospital (MGH), Brigham & Women's Hospital (BWH), as well as research conducted at those institutions and the Genotype-Tissue Expression (GTEx) consortium. The slides cover both normal and abnormal tissues from a wide range of disease types including neoplastic and inflammatory. For more details on dataset assembly and model training, we refer readers to the UNI study.

Organ	Included diagnoses
Brain	Oligodendroglioma, glioblastoma, cryptococcal meningitis, arteriovenous malformation, pilocytic astrocytoma, ependymoma
Lung	Lung adenocarcinoma, lung squamous cell carcinoma, mesothelioma, typical carcinoid tumor
GI	Colorectal adenocarcinoma, stomach adenocarcinoma, esophageal adenocarcinoma, esophageal squamous cell carcinoma
Urinary Tract	Chromophobe renal cell carcinoma, clear cell renal cell carcinoma, papillary renal cell carcinoma, urothelial carcinoma, xanthogranulomatous pyelonephritis, renal oncocytoma
Male Reproductive Tract	Testicular lymphoma, seminoma, prostatic adenocarcinoma, embryonal carcinoma, mixed germ cell tumor
Skin, Eye, and Connective Tissue	Cutaneous melanoma, leiomyosarcoma, myxofibrosarcoma, uveal melanoma, molluscum contagiosum, merkel cell carcinoma
Pancreaticohepatobiliary	Hepatocellular carcinoma, pancreatic adenocarcinoma, cholangiocarcinoma, macrovesicular steatosis, pancreatic neuroendocrine tumor
Endocrine	Papillary thyroid carcinoma, adrenal cortical carcinoma, pheochromocytoma, poorly differentiated thyroid carcinoma, hashimoto thyroiditis
Head, Neck, and Mediastinum	Oropharyngeal squamous cell carcinoma, mediastinal lymphoma, thymoma, tonsillar squamous cell carcinoma, branchial cleft cyst, adenoid cystic carcinoma
GYN	High-grade serous ovarian carcinoma, uterine endometrioid carcinoma, carcinosarcoma, cervical squamous cell carcinoma
Breast	Invasive lobular carcinoma, invasive ductal carcinoma, idiopathic granulomatous mastitis

Supplementary Data Table 5: Included diagnoses for PathQABench by organ. GI: Gastrointestinal, GYN: Gynecology.

Organ	PathQABench-Public	PathQABench-Private	Total
Brain	4	7	11
Lung	5	5	10
GI	6	4	10
Urinary Tract	4	6	10
Male Reproductive Tract	5	5	10
Skin, Eye, and Connective Tissue	5	5	10
Pancreaticohepatobiliary	5	5	10
Endocrine	5	4	9
Head, Neck, and Mediastinum	4	5	9
GYN	5	3	8
Breast	4	4	8
Total	52	53	105

Supplementary Data Table 6: Organ distribution of cases in PathQABench. GI: Gastrointestinal, GYN: Gynecology.

Organ	Multiple choice options
Brain	Pilocytic astrocytoma, ependymoma, glioblastoma, pleomorphic xanthoastrocytoma, subependymal giant cell astrocytoma, oligodendroglioma, subependymoma, ganglioglioma, arteriovenous malformation, cryptococcal meningitis
Lung	Lung adenocarcinoma, lung squamous cell carcinoma, typical carcinoid tumor, atypical carcinoid tumor, hamartoma of lung, meningothelial-like nodule, pneumocytoma, small cell carcinoma, mesothelioma, large cell neuroendocrine carcinoma
GI	Colorectal adenocarcinoma, stomach adenocarcinoma, esophageal adenocarcinoma, esophageal squamous cell carcinoma, sessile serrated adenoma, hyperplastic polyp, MALT lymphoma, ulcerative colitis, neuroendocrine tumor, gastrointestinal stromal tumor
Urinary Tract	Chromophobe renal cell carcinoma, clear cell renal cell carcinoma, papillary renal cell carcinoma, renal oncocytoma, urothelial carcinoma, cystitis cystica et glandularis, xanthogranulomatous pyelonephritis, nephroblastoma, mixed epithelial and stromal tumor, collecting duct carcinoma
Male Reproductive Tract	Testicular lymphoma, seminoma, prostatic adenocarcinoma, embryonal carcinoma, prostatic stromal sarcoma, mixed germ cell tumor, penile squamous cell carcinoma, molluscum contagiosum, epididymitis, choriocarcinoma
Skin, Eye, and Connective Tissue	Cutaneous melanoma, leiomyosarcoma, myxofibrosarcoma, basal cell carcinoma, liposarcoma, merkel cell carcinoma, mycosis fungoides, molluscum contagiosum, dermatofibrosarcoma, uveal melanoma
Pancreaticohepatobiliary	Hepatocellular carcinoma, pancreatic adenocarcinoma, cholangiocarcinoma, pancreatic neuroendocrine tumor, macrovesicular steatosis, cirrhosis, hepatoblastoma, intraductal papillary mucinous neoplasm, mucinous cystic neoplasm, fibrolamellar carcinoma
Endocrine	Papillary thyroid carcinoma, medullary thyroid carcinoma, adrenal cortical carcinoma, pheochromocytoma, poorly differentiated thyroid carcinoma, adrenal cortical adenoma, anaplastic thyroid carcinoma, hashimoto thyroiditis, follicular adenoma, multinodular goiter
Head, Neck, and Mediastinum	Oropharyngeal squamous cell carcinoma, mediastinal lymphoma, thymoma, tonsillar squamous cell carcinoma, branchial cleft cyst, adenoid cystic carcinoma, mucoepidermoid carcinoma, pleomorphic adenoma, mucosal melanoma, NUT carcinoma
GYN	High-grade serous ovarian carcinoma, low-grade serous ovarian carcinoma, uterine endometrioid carcinoma, carcinosarcoma, cervical squamous cell carcinoma, mucinous cystadenoma, borderline tumor, condyloma, clear cell carcinoma, brenner tumor
Breast	Invasive ductal carcinoma, invasive lobular carcinoma, phyllodes tumor, DCIS, LCIS, paget disease, metaplastic carcinoma, idiopathic granulomatous mastitis, usual ductal hyperplasia, atypical ductal hyperplasia

Supplementary Data Table 7: Options for multiple choice diagnostic questions in PathQABench by organ. For each organ system, the pathologist selected a set of 10 possible answers that encompasses the correct answers for all questions within that organ system as well as other relatively common diagnoses within that organ system. Finally, when each multiple choice question is constructed using the above options, the order in each the options appear is randomized to ensure the correct answer is equally likely to be slotted into any location among the possible choices. GI: Gastrointestinal, GYN: Gynecology.

Model	Combined	Combined w/ Context
PathChat	0.781 (0.695, 0.857)	0.895 (0.829, 0.952)
LLaVA-Med	0.143 (0.076, 0.210)	0.286 (0.200, 0.371)
LLaVA 1.5	0.257 (0.171, 0.343)	0.505 (0.400, 0.610)

Supplementary Data Table 8: Performance on PathQABench multiple-choice diagnostic questions. Accuracy is reported on the full set of PathQABench multiple choice questions ($n = 105$) in both the image only evaluation setting and the image + clinical context evaluation setting (denoted as "w/ Context"). 95% confidence intervals from bootstrapping are included in parentheses. For more details see **MLLM evaluation** in **Methods**.

Model	PathQABench-Public	PathQABench-Public w/ Context
PathChat	0.788 (0.673, 0.904)	0.904 (0.827, 0.981)
GPT4V	0.250 (0.135, 0.365)	0.635 (0.500, 0.769)
LLaVA-Med	0.115 (0.038, 0.212)	0.250 (0.135, 0.365)
LLaVA 1.5	0.288 (0.173, 0.423)	0.538 (0.404, 0.673)

Supplementary Data Table 9: Performance on PathQABench-Public multiple-choice diagnostic questions. Accuracy is reported on the PathQABench-Public multiple choice questions ($n = 52$) in both the image only evaluation setting and the image with clinical context evaluation setting (denoted as "w/ Context"). 95% confidence intervals from bootstrapping are included in parentheses. For more details see **MLLM evaluation** in **Methods**. ∗Note that due to guardrails implemented by GPT4V, only 28 / 52 questions submitted to the API yielded successful answers for PathQABench-Public in the image only setting and 47 / 52 in the image with context setting (a maximum number of 3 attempts were made for each question). An unsuccessful query was treated as incorrect since the response did not address the question. We also report performance just on the subset of questions that yielded successful queries for GPT4V in **Supplementary Data Table 11**. For more details see **Evaluation of GPT4V** in **Methods**

.

Model	PathQABench-Private	PathQABench-Private w/ Context
PathChat	0.774 (0.660, 0.868)	0.887 (0.792, 0.962)
LLaVA-Med	0.170 (0.075, 0.283)	0.321 (0.208, 0.453)
LLaVA 1.5	0.226 (0.113, 0.340)	0.472 (0.340, 0.604)

Supplementary Data Table 10: Performance on PathQABench-Private multiple-choice diagnostic questions. Accuracy is reported on the PathQABench-Private multiple choice questions ($n = 53$) in both the image only evaluation setting and the image + clinical context evaluation setting (denoted as "w/ Context"). 95% confidence intervals from bootstrapping are included in parentheses. For more details see **MLLM evaluation** in **Methods**.

Model	PathQABench-Public	PathQABench-Public w/ Context
PathChat	0.786 (0.607, 0.929)	0.915 (0.830, 0.979)
GPT4V	0.464 (0.286, 0.643)	0.702 (0.574, 0.830)
LLaVA-Med	0.071 (0.000, 0.179)	0.234 (0.128, 0.362)
LLaVA 1.5	0.286 (0.143, 0.464)	0.574 (0.426, 0.723)

Supplementary Data Table 11: Performance on PathQABench-Public multiple-choice diagnostic questions, restricted to successful GPT4V queries. Accuracy is reported on the subset of PathQABench-Public multiple choice questions for which GPTV successfully answered the question (*i.e.*, did not refuse to give an answer within the maximum number of 3 attempted API calls), for both the image only evaluation setting ($n = 28$) and the image + clinical context evaluation setting (denoted as "w/ Context", $n = 47$). 95% confidence intervals from bootstrapping are included in parentheses. For more details see **MLLM evaluation** in **Methods**.

PathChat *vs.* model	Win	Tie	Lose
GPT4V	0.565 (0.492, 0.612)	0.212 (0.069, 0.308)	0.223 (0.165, 0.265)
LLaVA-Med	0.742 (0.708, 0.750)	0.154 (0.069, 0.215)	0.100 (0.085, 0.119)
LLaVA 1.5	0.677 (0.669, 0.700)	0.215 (0.088, 0.235)	0.112 (0.077, 0.154)

Supplementary Data Table 12: Head-to-head performance of PathChat against other MLLMs on PathQABench open-ended questions. For each open-ended question, 7 pathologists independently ranked the outputs of PathChat and competing models (GPT4V, LLaVA-Med, LLaVA 1.5), blinded to which model produced which response (see **MLLM evaluation** for more details). For each pathologist's evaluation, we compute the win/tie/lose rate of PathChat against said model for the 260 open-ended questions. We report the median win/tie/lose rates across the evaluations of 7 pathologists. See **Supplementary Data Table 13** for metrics from each individual pathologist. Lose: said model is ranked higher than PathChat; Tie: PathChat is tied with the model in ranking; Win: PathChat is ranked higher than the model. 95% confidence intervals from bootstrapping are included in parentheses. ∗Note that due to guardrails implemented by GPT4V, 38 / 260 queries to the API yielded unsuccessful answers for PathQABench-Public (a maximum number of 3 attempts were made for each question). Regardless, all responses were reviewed by pathologists without special treatment but we also report performance of each model after removing obviously unsuccessful queries for GPT4V in **Supplementary Data Table 47**. For more details see **Evaluation of GPT4V** in **Methods**.

Pathologist	PathChat *vs.* model	Win	Tie	Lose
1	GPT4V	0.612 (0.554, 0.669)	0.223 (0.177, 0.273)	0.165 (0.123, 0.212)
	LLaVA-Med	0.746 (0.692, 0.800)	0.154 (0.115, 0.192)	0.100 (0.065, 0.138)
	LLaVA 1.5	0.673 (0.619, 0.727)	0.215 (0.169, 0.265)	0.112 (0.077, 0.150)
2	GPT4V	0.481 (0.419, 0.535)	0.308 (0.250, 0.365)	0.212 (0.162, 0.262)
	LLaVA-Med	0.727 (0.677, 0.781)	0.188 (0.142, 0.235)	0.085 (0.054, 0.123)
	LLaVA 1.5	0.692 (0.638, 0.746)	0.235 (0.185, 0.285)	0.073 (0.042, 0.108)
3	GPT4V	0.604 (0.542, 0.665)	0.131 (0.092, 0.173)	0.265 (0.212, 0.315)
	LLaVA-Med	0.750 (0.700, 0.804)	0.131 (0.092, 0.169)	0.119 (0.081, 0.162)
	LLaVA 1.5	0.700 (0.646, 0.754)	0.146 (0.104, 0.188)	0.154 (0.112, 0.200)
4	GPT4V	0.565 (0.512, 0.623)	0.212 (0.158, 0.262)	0.223 (0.177, 0.277)
	LLaVA-Med	0.665 (0.608, 0.719)	0.227 (0.181, 0.281)	0.108 (0.073, 0.146)
	LLaVA 1.5	0.669 (0.615, 0.723)	0.219 (0.173, 0.273)	0.112 (0.077, 0.154)
5	GPT4V	0.542 (0.485, 0.604)	0.388 (0.327, 0.446)	0.069 (0.042, 0.100)
	LLaVA-Med	0.708 (0.650, 0.758)	0.215 (0.162, 0.269)	0.077 (0.046, 0.112)
	LLaVA 1.5	0.619 (0.558, 0.677)	0.304 (0.246, 0.365)	0.077 (0.046, 0.112)
6	GPT4V	0.681 (0.627, 0.735)	0.069 (0.038, 0.104)	0.250 (0.200, 0.300)
	LLaVA-Med	0.742 (0.688, 0.792)	0.069 (0.038, 0.104)	0.188 (0.142, 0.235)
	LLaVA 1.5	0.677 (0.623, 0.738)	0.088 (0.058, 0.123)	0.235 (0.185, 0.285)
7	GPT4V	0.492 (0.431, 0.554)	0.058 (0.031, 0.088)	0.450 (0.392, 0.512)
	LLaVA-Med	0.858 (0.812, 0.896)	0.042 (0.019, 0.069)	0.100 (0.065, 0.138)
	LLaVA 1.5	0.788 (0.738, 0.835)	0.065 (0.038, 0.096)	0.146 (0.104, 0.192)

Supplementary Data Table 13: Head-to-head performance of PathChat against other MLLMs on PathQABench open-ended questions as evaluated by 7 individual pathologists. For each open-ended question, 7 pathologists independently ranked the outputs of PathChat and competing models (GPT4V, LLaVA-Med, LLaVA 1.5), blinded to which model produced which response (see **MLLM evaluation** for more details). For each pathologist's evaluation, we compute the win/tie/lose rate of PathChat against said model for the 260 questions. We report the win/tie/lose rates for each pathologist. Lose: said model is ranked higher than PathChat; Tie: PathChat is tied with the model in ranking; Win: PathChat is ranked higher than the model. 95% confidence intervals from bootstrapping are included in parentheses. For more details see **PathChat model evaluation** in **Methods**. See **Supplementary Data Table 12** for aggregated metrics across all 7 pathologists. ∗Note that due to guardrails implemented by GPT4V, 38 / 260 queries to the API yielded unsuccessful answers for PathQABench-Public (a maximum number of 3 attempts were made for each question). Regardless, all responses were reviewed by pathologists without special treatment but we also report performance of each model after removing obviously unsuccessful queries for GPT4V in **Supplementary Data Table 48**. For more details see **Evaluation of GPT4V** in **Methods**.

Model	Consensus ($n = 235$)	Pathologist 1 ($n = 260$)	Pathologist 2 ($n = 260$)
PathChat	0.787 (0.732, 0.838)	0.777 (0.727, 0.831)	0.762 (0.712, 0.812)
GPT4V	0.523 (0.464, 0.587)	0.538 (0.481, 0.600)	0.527 (0.469, 0.589)
LLaVA-Med	0.306 (0.251, 0.370)	0.312 (0.258, 0.369)	0.300 (0.250, 0.358)
LLaVA 1.5	0.298 (0.238, 0.362)	0.315 (0.262, 0.373)	0.281 (0.223, 0.335)

Supplementary Data Table 14: Proportion of open-ended questions in PathQABench correctly answered by each model. Two pathologists first independently evaluated the correctness of each model's outputs. Next, after discussion, consensus was reached on 235 of the 260 questions (See **MLLM evaluation** in **Methods** for details). We report the performance on both the subset of cases for which a consensus was reached (using the consensus as ground truth) and also the performance according to each pathologist (including cases for which a consensus was not ultimately achieved). 95% confidence intervals from bootstrapping are included in parentheses. See **Supplementary Data Tables 17–19 and 28–30** for accuracy stratified by category and subcategory. ∗We noted that due to guardrails implemented by GPT4V, 38 / 260 questions in total and 33 / 235 questions of the consensus subset that were submitted to the API yielded unsuccessful answers for PathQABench-Public (a maximum number of 3 attempts were made for each question). However, all responses were reviewed by pathologists and an unsuccessful query was treated as incorrect if the response did not address the question. We also report performance of the models after removing such obviously unsuccessful queries for GPT4V in Table **Supplementary Data Table 40**. For more details see **Evaluation of GPT4V** in **Methods**.

Broad Category	Description
Microscopy	Questions test the ability of models to generate accurate and detailed morphological descriptions of histology microscopy images and assess clinically relevant features such as tumor differentiation and grade<br>Sub-categories: Microscopic description, Differentiation, Grading
Diagnosis	Questions test the ability of models to directly suggest a reasonable diagnosis based on the histology image available and relevant clinical context<br>Sub-categories: Diagnosis
Clinical	Questions test the ability of models to retrieve clinically relevant background knowledge about the disease in question, including risk factors, prognosis and treatment.<br>Sub-categories: Risks, Prognosis, Treatment
Ancillary testing	Questions test the ability of models to suggest additional testing such as IHCs and molecular to confirm a specific diagnosis<br>Sub-categories: IHC, Molecular, Further testing

Supplementary Data Table 15: Categorization of open-ended questions in PathQABench. Number of questions in each category is summarized in **Supplementary Data Table 16**. Some questions may fit the description of more than one category or sub-category. Examples of each category can be found in **Extended Data Figure 7**.

Broad category	Count	Sub-category	Count
Microscopy	109	Microscopic Description	73
		Differentiation	32
		Grading	38
Diagnosis	87	Diagnosis	87
Clinical	68	Risk Factors	8
		Prognosis	50
		Treatment	51
Ancillary Testing	87	IHC	44
		Molecular	42
		Other Testing	12

Supplementary Data Table 16: Broad and sub-categories for PathQABench open-ended questions. In total of 260 questions were curated and reviewed by a board-certified anatomic pathologist from 52 cases in PathQABench-Public. Each question may fall under more than one category.

Category	PathChat	GPT4V∗	LLaVA-Med	LLaVA 1.5
Microscopy	0.733 (0.653, 0.822)	0.228 (0.149, 0.317)	0.228 (0.158, 0.307)	0.178 (0.109, 0.257)
Diagnosis	0.785 (0.684, 0.873)	0.316 (0.215, 0.430)	0.241 (0.152, 0.342)	0.177 (0.101, 0.266)
Clinical	0.803 (0.705, 0.902)	0.885 (0.803, 0.967)	0.508 (0.393, 0.623)	0.623 (0.508, 0.738)
Ancillary Testing	0.803 (0.711, 0.882)	0.895 (0.829, 0.961)	0.355 (0.250, 0.461)	0.447 (0.342, 0.553)

Supplementary Data Table 17: Proportion of open-ended questions in the consensus subset of PathQABench correctly answered by each model, stratified by broad categories. Two pathologists first independently evaluated the correctness of each model's outputs. Next, after discussion, consensus was reached on 235 of the 260 questions (See **MLLM evaluation** in **Methods** for details). We report the performance on the subset of cases for which a consensus was reached (using the consensus as ground truth). 95% confidence intervals from bootstrapping are included in parentheses. ∗We noted that due to guardrails implemented by GPT4V, 33 / 235 questions of the consensus subset that were submitted to the API yielded unsuccessful answers for PathQABench-Public (a maximum number of 3 attempts were made for each question). However, all responses were reviewed by pathologists and an unsuccessful query was treated as incorrect if the response did not address the question. We also report performance of the models after removing such obviously unsuccessful queries for GPT4V in **Supplementary Data Table 41**. For more details see **Evaluation of GPT4V** in **Methods**.

Category	PathChat	GPT4V*	LLaVA-Med	LLaVA 1.5
Microscopy	0.734 (0.651, 0.817)	0.248 (0.165, 0.330)	0.220 (0.147, 0.294)	0.183 (0.119, 0.257)
Diagnosis	0.736 (0.644, 0.816)	0.333 (0.241, 0.437)	0.241 (0.161, 0.333)	0.195 (0.126, 0.276)
Clinical	0.794 (0.691, 0.882)	0.868 (0.779, 0.941)	0.515 (0.397, 0.632)	0.632 (0.515, 0.750)
Ancillary Testing	0.805 (0.713, 0.885)	0.908 (0.839, 0.966)	0.368 (0.264, 0.471)	0.471 (0.368, 0.575)

Supplementary Data Table 18: Proportion of open-ended questions in PathQABench correctly answered by each model as evaluated by pathologist 1, stratified by broad categories. Two pathologists first independently evaluated the correctness of each model's outputs. Next, after discussion, consensus was reached on 235 of the 260 questions (See **MLLM evaluation** in **Methods** for details). We report the performance according to pathologist 1 (including cases for which a consensus was not ultimately achieved). 95% confidence intervals from bootstrapping are included in parentheses. *We noted that due to guardrails implemented by GPT4V, 33 / 235 questions of the consensus subset that were submitted to the API yielded unsuccessful answers for PathQABench-Public (a maximum number of 3 attempts were made for each question). However, all responses were reviewed by pathologists and an unsuccessful query was treated as incorrect if the response did not address the question. We also report performance of the models after removing such obviously unsuccessful queries for GPT4V in **Supplementary Data Table 42**. For more details see **Evaluation of GPT4V** in **Methods**.

Category	PathChat	GPT4V*	LLaVA-Med	LLaVA 1.5
Microscopy	0.706 (0.615, 0.789)	0.248 (0.165, 0.330)	0.229 (0.156, 0.303)	0.165 (0.101, 0.239)
Diagnosis	0.747 (0.655, 0.839)	0.333 (0.241, 0.437)	0.241 (0.161, 0.333)	0.172 (0.092, 0.253)
Clinical	0.779 (0.676, 0.882)	0.853 (0.765, 0.926)	0.500 (0.382, 0.618)	0.559 (0.441, 0.676)
Ancillary Testing	0.782 (0.690, 0.862)	0.874 (0.804, 0.931)	0.322 (0.230, 0.425)	0.414 (0.310, 0.517)

Supplementary Data Table 19: Proportion of open-ended questions in PathQABench correctly answered by each model as evaluated by pathologist 2, stratified by broad categories. Two pathologists first independently evaluated the correctness of each model's outputs. Next, after discussion, consensus was reached on 235 of the 260 questions (See **MLLM evaluation** in **Methods** for details). We report the performance according to pathologist 2 (including cases for which a consensus was not ultimately achieved). 95% confidence intervals from bootstrapping are included in parentheses. *We noted that due to guardrails implemented by GPT4V, 33 / 235 questions of the consensus subset that were submitted to the API yielded unsuccessful answers for PathQABench-Public (a maximum number of 3 attempts were made for each question). However, all responses were reviewed by pathologists and an unsuccessful query was treated as incorrect if the response did not address the question. We also report performance of the models after removing such obviously unsuccessful queries for GPT4V in **Supplementary Data Table 43**. For more details see **Evaluation of GPT4V** in **Methods**.

Category	PathChat *vs.* model	Win	Tie	Lose
Microscopy	GPT4V	0.706 (0.633, 0.761)	0.156 (0.028, 0.220)	0.138 (0.092, 0.193)
	LLaVA-Med	0.688 (0.670, 0.734)	0.165 (0.064, 0.229)	0.110 (0.101, 0.156)
	LLaVA 1.5	0.716 (0.697, 0.743)	0.174 (0.101, 0.220)	0.101 (0.083, 0.110)
Diagnosis	GPT4V	0.713 (0.644, 0.747)	0.149 (0.046, 0.172)	0.138 (0.103, 0.230)
	LLaVA-Med	0.724 (0.701, 0.770)	0.161 (0.092, 0.241)	0.080 (0.069, 0.092)
	LLaVA 1.5	0.759 (0.724, 0.770)	0.149 (0.103, 0.184)	0.092 (0.080, 0.092)
Clinical	GPT4V	0.441 (0.235, 0.471)	0.206 (0.074, 0.441)	0.338 (0.235, 0.471)
	LLaVA-Med	0.735 (0.676, 0.794)	0.118 (0.103, 0.176)	0.103 (0.059, 0.147)
	LLaVA 1.5	0.588 (0.485, 0.632)	0.191 (0.088, 0.353)	0.206 (0.088, 0.309)
Ancillary Testing	GPT4V	0.448 (0.253, 0.460)	0.241 (0.126, 0.391)	0.356 (0.299, 0.414)
	LLaVA-Med	0.782 (0.736, 0.862)	0.126 (0.046, 0.184)	0.092 (0.046, 0.115)
	LLaVA 1.5	0.644 (0.621, 0.678)	0.195 (0.115, 0.253)	0.184 (0.103, 0.195)

Supplementary Data Table 20: Head-to-head performance of PathChat against other MLLMs on PathQABench open-ended questions, stratified by category. For each open-ended question, 7 pathologists independently ranked the outputs of PathChat and competing models (GPT4V, LLaVA-Med, LLaVA 1.5), blinded to which model produced which response (see **MLLM evaluation** for more details). For each pathologist's evaluation, we compute the win/tie/lose rate of PathChat against said model for the 260 open-ended questions. We report the median win/tie/lose rates across the evaluations of 7 pathologists. See **Supplementary Data Table 21–27** for metrics from each individual pathologist. Lose: said model is ranked higher than PathChat; Tie: PathChat is tied with the model in ranking; Win: PathChat is ranked higher than the model. 95% confidence intervals from bootstrapping are included in parentheses. ∗Note that due to guardrails implemented by GPT4V, 38 / 260 queries to the API yielded unsuccessful answers for PathQABench-Public (a maximum number of 3 attempts were made for each question). Regardless, all responses were reviewed by pathologists without special treatment but we also report performance of each model after removing obviously unsuccessful queries for GPT4V in **Supplementary Data Table 49**. For more details see **Evaluation of GPT4V** in **Methods**.

Category	PathChat *vs.* model	Win	Tie	Lose
Microscopy	GPT4V	0.706 (0.624, 0.789)	0.183 (0.110, 0.257)	0.110 (0.055, 0.165)
	LLaVA-Med	0.688 (0.596, 0.771)	0.202 (0.128, 0.284)	0.110 (0.055, 0.174)
	LLaVA 1.5	0.716 (0.633, 0.798)	0.183 (0.110, 0.257)	0.101 (0.046, 0.156)
Diagnosis	GPT4V	0.747 (0.655, 0.839)	0.149 (0.080, 0.230)	0.103 (0.046, 0.172)
	LLaVA-Med	0.770 (0.678, 0.851)	0.161 (0.092, 0.241)	0.069 (0.023, 0.126)
	LLaVA 1.5	0.736 (0.644, 0.828)	0.172 (0.092, 0.253)	0.092 (0.034, 0.149)
Clinical	GPT4V	0.471 (0.353, 0.588)	0.294 (0.191, 0.397)	0.235 (0.147, 0.338)
	LLaVA-Med	0.794 (0.691, 0.882)	0.118 (0.044, 0.191)	0.088 (0.029, 0.162)
	LLaVA 1.5	0.485 (0.368, 0.603)	0.309 (0.206, 0.412)	0.206 (0.118, 0.309)
Ancillary Testing	GPT4V	0.448 (0.356, 0.540)	0.253 (0.161, 0.334)	0.299 (0.207, 0.391)
	LLaVA-Med	0.816 (0.736, 0.885)	0.069 (0.023, 0.126)	0.115 (0.057, 0.184)
	LLaVA 1.5	0.632 (0.529, 0.736)	0.218 (0.138, 0.299)	0.149 (0.080, 0.230)

Supplementary Data Table 21: Head-to-head performance of PathChat against other MLLMs on PathQABench open-ended questions as evaluated by pathologist 1, stratified by category. For each open-ended question, 7 pathologists independently ranked the outputs of PathChat and competing models (GPT4V, LLaVA-Med, LLaVA 1.5), blinded to which model produced which response (see **MLLM evaluation** for more details). For each pathologist's evaluation, we compute the win/tie/lose rate of PathChat against said model for the 260 open-ended questions. We report the win/tie/lose rates according to the assessment of pathologist 1. Lose: said model is ranked higher than PathChat; Tie: PathChat is tied with the model in ranking; Win: PathChat is ranked higher than the model. 95% confidence intervals from bootstrapping are included in parentheses. ∗Note that due to guardrails implemented by GPT4V, 38 / 260 queries to the API yielded unsuccessful answers for PathQABench-Public (a maximum number of 3 attempts were made for each question). Regardless, all responses were reviewed by pathologists without special treatment but we also report performance of each model after removing obviously unsuccessful queries for GPT4V in **Supplementary Data Table 50**. For more details see **Evaluation of GPT4V** in **Methods**.

Category	PathChat *vs.* model	Win	Tie	Lose
Microscopy	GPT4V	0.633 (0.541, 0.716)	0.229 (0.156, 0.312)	0.138 (0.083, 0.202)
	LLaVA-Med	0.670 (0.578, 0.752)	0.229 (0.147, 0.312)	0.101 (0.055, 0.165)
	LLaVA 1.5	0.697 (0.615, 0.780)	0.220 (0.147, 0.303)	0.083 (0.037, 0.138)
Diagnosis	GPT4V	0.644 (0.540, 0.736)	0.218 (0.138, 0.310)	0.138 (0.069, 0.207)
	LLaVA-Med	0.701 (0.598, 0.793)	0.241 (0.149, 0.333)	0.057 (0.011, 0.103)
	LLaVA 1.5	0.759 (0.667, 0.839)	0.184 (0.103, 0.276)	0.057 (0.011, 0.103)
Clinical	GPT4V	0.235 (0.147, 0.338)	0.441 (0.324, 0.559)	0.324 (0.206, 0.441)
	LLaVA-Med	0.735 (0.632, 0.838)	0.162 (0.074, 0.250)	0.103 (0.044, 0.176)
	LLaVA 1.5	0.559 (0.441, 0.676)	0.353 (0.250, 0.456)	0.088 (0.029, 0.162)
Ancillary Testing	GPT4V	0.253 (0.172, 0.345)	0.391 (0.287, 0.494)	0.356 (0.264, 0.460)
	LLaVA-Med	0.782 (0.690, 0.862)	0.138 (0.069, 0.218)	0.080 (0.023, 0.138)
	LLaVA 1.5	0.644 (0.529, 0.736)	0.253 (0.172, 0.356)	0.103 (0.046, 0.172)

Supplementary Data Table 22: Head-to-head performance of PathChat against other MLLMs on PathQABench open-ended questions as evaluated by pathologist 2, stratified by category. For each open-ended question, 7 pathologists independently ranked the outputs of PathChat and competing models (GPT4V, LLaVA-Med, LLaVA 1.5), blinded to which model produced which response (see **MLLM evaluation** for more details). For each pathologist's evaluation, we compute the win/tie/lose rate of PathChat against said model for the 260 open-ended questions. We report the win/tie/lose rates according to the assessment of pathologist 2. Lose: said model is ranked higher than PathChat; Tie: PathChat is tied with the model in ranking; Win: PathChat is ranked higher than the model. 95% confidence intervals from bootstrapping are included in parentheses. ∗Note that due to guardrails implemented by GPT4V, 38 / 260 queries to the API yielded unsuccessful answers for PathQABench-Public (a maximum number of 3 attempts were made for each question). Regardless, all responses were reviewed by pathologists without special treatment but we also report performance of each model after removing obviously unsuccessful queries for GPT4V in **Supplementary Data Table 51**. For more details see **Evaluation of GPT4V** in **Methods**.

Category	PathChat *vs.* model	Win	Tie	Lose
Microscopy	GPT4V	0.706 (0.615, 0.789)	0.147 (0.083, 0.220)	0.147 (0.083, 0.211)
	LLaVA-Med	0.734 (0.651, 0.817)	0.128 (0.073, 0.193)	0.138 (0.073, 0.202)
	LLaVA 1.5	0.716 (0.624, 0.798)	0.174 (0.110, 0.248)	0.110 (0.055, 0.174)
Diagnosis	GPT4V	0.713 (0.621, 0.793)	0.172 (0.092, 0.253)	0.115 (0.057, 0.184)
	LLaVA-Med	0.759 (0.667, 0.839)	0.149 (0.080, 0.230)	0.092 (0.034, 0.161)
	LLaVA 1.5	0.770 (0.678, 0.851)	0.149 (0.080, 0.230)	0.080 (0.034, 0.138)
Clinical	GPT4V	0.471 (0.353, 0.588)	0.059 (0.015, 0.118)	0.471 (0.353, 0.588)
	LLaVA-Med	0.765 (0.662, 0.853)	0.118 (0.044, 0.191)	0.118 (0.044, 0.191)
	LLaVA 1.5	0.588 (0.471, 0.706)	0.088 (0.029, 0.162)	0.324 (0.221, 0.441)
Ancillary Testing	GPT4V	0.460 (0.356, 0.563)	0.126 (0.057, 0.207)	0.414 (0.310, 0.517)
	LLaVA-Med	0.770 (0.690, 0.851)	0.126 (0.057, 0.195)	0.103 (0.046, 0.172)
	LLaVA 1.5	0.655 (0.563, 0.759)	0.149 (0.069, 0.230)	0.195 (0.115, 0.287)

Supplementary Data Table 23: Head-to-head performance of PathChat against other MLLMs on PathQABench open-ended questions as evaluated by pathologist 3, stratified by category. For each open-ended question, 7 pathologists independently ranked the outputs of PathChat and competing models (GPT4V, LLaVA-Med, LLaVA 1.5), blinded to which model produced which response (see **MLLM evaluation** for more details). For each pathologist's evaluation, we compute the win/tie/lose rate of PathChat against said model for the 260 open-ended questions. We report the win/tie/lose rates according to the assessment of pathologist 3. Lose: said model is ranked higher than PathChat; Tie: PathChat is tied with the model in ranking; Win: PathChat is ranked higher than the model. 95% confidence intervals from bootstrapping are included in parentheses. ∗Note that due to guardrails implemented by GPT4V, 38 / 260 queries to the API yielded unsuccessful answers for PathQABench-Public (a maximum number of 3 attempts were made for each question). Regardless, all responses were reviewed by pathologists without special treatment but we also report performance of each model after removing obviously unsuccessful queries for GPT4V in **Supplementary Data Table 52**. For more details see **Evaluation of GPT4V** in **Methods**.

Category	PathChat *vs.* model	Win	Tie	Lose
Microscopy	GPT4V	0.688 (0.606, 0.771)	0.220 (0.138, 0.294)	0.092 (0.046, 0.147)
	LLaVA-Med	0.651 (0.560, 0.743)	0.266 (0.174, 0.349)	0.083 (0.037, 0.138)
	LLaVA 1.5	0.706 (0.624, 0.789)	0.248 (0.165, 0.321)	0.046 (0.009, 0.083)
Diagnosis	GPT4V	0.667 (0.563, 0.759)	0.126 (0.057, 0.207)	0.207 (0.126, 0.287)
	LLaVA-Med	0.667 (0.563, 0.770)	0.253 (0.161, 0.356)	0.080 (0.034, 0.138)
	LLaVA 1.5	0.724 (0.621, 0.816)	0.195 (0.115, 0.276)	0.080 (0.023, 0.138)
Clinical	GPT4V	0.426 (0.309, 0.544)	0.206 (0.118, 0.309)	0.368 (0.250, 0.471)
	LLaVA-Med	0.676 (0.559, 0.779)	0.176 (0.088, 0.279)	0.147 (0.074, 0.235)
	LLaVA 1.5	0.632 (0.515, 0.735)	0.191 (0.103, 0.279)	0.176 (0.103, 0.279)
Ancillary Testing	GPT4V	0.402 (0.299, 0.506)	0.241 (0.149, 0.333)	0.356 (0.264, 0.460)
	LLaVA-Med	0.655 (0.552, 0.759)	0.184 (0.103, 0.276)	0.161 (0.080, 0.241)
	LLaVA 1.5	0.621 (0.517, 0.724)	0.195 (0.115, 0.276)	0.184 (0.103, 0.264)

Supplementary Data Table 24: Head-to-head performance of PathChat against other MLLMs on PathQABench open-ended questions as evaluated by pathologist 4, stratified by category. For each open-ended question, 7 pathologists independently ranked the outputs of PathChat and competing models (GPT4V, LLaVA-Med, LLaVA 1.5), blinded to which model produced which response (see **MLLM evaluation** for more details). For each pathologist's evaluation, we compute the win/tie/lose rate of PathChat against said model for the 260 open-ended questions. We report the win/tie/lose rates according to the assessment of pathologist 4. Lose: said model is ranked higher than PathChat; Tie: PathChat is tied with the model in ranking; Win: PathChat is ranked higher than the model. 95% confidence intervals from bootstrapping are included in parentheses. ∗Note that due to guardrails implemented by GPT4V, 38 / 260 queries to the API yielded unsuccessful answers for PathQABench-Public (a maximum number of 3 attempts were made for each question). Regardless, all responses were reviewed by pathologists without special treatment but we also report performance of each model after removing obviously unsuccessful queries for GPT4V in **Supplementary Data Table 53**. For more details see **Evaluation of GPT4V** in **Methods**.

Category	PathChat *vs.* model	Win	Tie	Lose
Microscopy	GPT4V	0.761 (0.688, 0.844)	0.156 (0.092, 0.220)	0.083 (0.037, 0.138)
	LLaVA-Med	0.725 (0.642, 0.807)	0.165 (0.101, 0.239)	0.110 (0.055, 0.174)
	LLaVA 1.5	0.743 (0.661, 0.826)	0.147 (0.092, 0.211)	0.110 (0.055, 0.174)
Diagnosis	GPT4V	0.782 (0.690, 0.862)	0.149 (0.080, 0.230)	0.069 (0.023, 0.126)
	LLaVA-Med	0.713 (0.621, 0.805)	0.207 (0.126, 0.288)	0.080 (0.023, 0.149)
	LLaVA 1.5	0.759 (0.655, 0.851)	0.149 (0.080, 0.219)	0.092 (0.034, 0.161)
Clinical	GPT4V	0.191 (0.103, 0.279)	0.721 (0.618, 0.824)	0.088 (0.029, 0.162)
	LLaVA-Med	0.618 (0.500, 0.735)	0.338 (0.221, 0.441)	0.044 (0.000, 0.103)
	LLaVA 1.5	0.426 (0.309, 0.544)	0.485 (0.368, 0.603)	0.088 (0.029, 0.162)
Ancillary Testing	GPT4V	0.218 (0.126, 0.299)	0.713 (0.621, 0.816)	0.069 (0.023, 0.126)
	LLaVA-Med	0.736 (0.644, 0.828)	0.230 (0.149, 0.322)	0.034 (0.000, 0.069)
	LLaVA 1.5	0.425 (0.322, 0.517)	0.517 (0.425, 0.632)	0.057 (0.011, 0.103)

Supplementary Data Table 25: Head-to-head performance of PathChat against other MLLMs on PathQABench open-ended questions as evaluated by pathologist 5, stratified by category. For each open-ended question, 7 pathologists independently ranked the outputs of PathChat and competing models (GPT4V, LLaVA-Med, LLaVA 1.5), blinded to which model produced which response (see **MLLM evaluation** for more details). For each pathologist's evaluation, we compute the win/tie/lose rate of PathChat against said model for the 260 open-ended questions. We report the win/tie/lose rates according to the assessment of pathologist 5. Lose: said model is ranked higher than PathChat; Tie: PathChat is tied with the model in ranking; Win: PathChat is ranked higher than the model. 95% confidence intervals from bootstrapping are included in parentheses. ∗Note that due to guardrails implemented by GPT4V, 38 / 260 queries to the API yielded unsuccessful answers for PathQABench-Public (a maximum number of 3 attempts were made for each question). Regardless, all responses were reviewed by pathologists without special treatment but we also report performance of each model after removing obviously unsuccessful queries for GPT4V in **Supplementary Data Table 54**. For more details see **Evaluation of GPT4V** in **Methods**.

Category	PathChat *vs.* model	Win	Tie	Lose
Microscopy	GPT4V	0.780 (0.706, 0.853)	0.028 (0.000, 0.064)	0.193 (0.119, 0.266)
	LLaVA-Med	0.670 (0.587, 0.752)	0.064 (0.027, 0.110)	0.266 (0.183, 0.349)
	LLaVA 1.5	0.688 (0.606, 0.771)	0.101 (0.046, 0.156)	0.211 (0.138, 0.294)
Diagnosis	GPT4V	0.747 (0.655, 0.839)	0.023 (0.000, 0.057)	0.230 (0.138, 0.322)
	LLaVA-Med	0.724 (0.632, 0.816)	0.092 (0.034, 0.149)	0.184 (0.103, 0.276)
	LLaVA 1.5	0.690 (0.586, 0.782)	0.103 (0.046, 0.172)	0.207 (0.126, 0.299)
Clinical	GPT4V	0.544 (0.426, 0.676)	0.118 (0.044, 0.191)	0.338 (0.221, 0.441)
	LLaVA-Med	0.691 (0.588, 0.794)	0.103 (0.044, 0.176)	0.206 (0.103, 0.294)
	LLaVA 1.5	0.618 (0.500, 0.750)	0.074 (0.015, 0.147)	0.309 (0.206, 0.412)
Ancillary Testing	GPT4V	0.563 (0.460, 0.667)	0.138 (0.069, 0.207)	0.299 (0.207, 0.391)
	LLaVA-Med	0.862 (0.782, 0.931)	0.046 (0.011, 0.092)	0.092 (0.034, 0.161)
	LLaVA 1.5	0.678 (0.575, 0.770)	0.046 (0.011, 0.092)	0.276 (0.184, 0.368)

Supplementary Data Table 26: Head-to-head performance of PathChat against other MLLMs on PathQABench open-ended questions as evaluated by pathologist 6, stratified by category. For each open-ended question, 7 pathologists independently ranked the outputs of PathChat and competing models (GPT4V, LLaVA-Med, LLaVA 1.5), blinded to which model produced which response (see **MLLM evaluation** for more details). For each pathologist's evaluation, we compute the win/tie/lose rate of PathChat against said model for the 260 open-ended questions. We report the win/tie/lose rates according to the assessment of pathologist 6. Lose: said model is ranked higher than PathChat; Tie: PathChat is tied with the model in ranking; Win: PathChat is ranked higher than the model. 95% confidence intervals from bootstrapping are included in parentheses. ∗Note that due to guardrails implemented by GPT4V, 38 / 260 queries to the API yielded unsuccessful answers for PathQABench-Public (a maximum number of 3 attempts were made for each question). Regardless, all responses were reviewed by pathologists without special treatment but we also report performance of each model after removing obviously unsuccessful queries for GPT4V in **Supplementary Data Table 55**. For more details see **Evaluation of GPT4V** in **Methods**.

Category	PathChat *vs.* model	Win	Tie	Lose
Microscopy	GPT4V	0.541 (0.450, 0.633)	0.018 (0.000, 0.046)	0.440 (0.349, 0.532)
	LLaVA-Med	0.798 (0.725, 0.872)	0.046 (0.009, 0.083)	0.156 (0.092, 0.229)
	LLaVA 1.5	0.853 (0.780, 0.917)	0.046 (0.009, 0.092)	0.101 (0.046, 0.165)
Diagnosis	GPT4V	0.552 (0.448, 0.644)	0.046 (0.011, 0.092)	0.402 (0.299, 0.517)
	LLaVA-Med	0.839 (0.770, 0.920)	0.069 (0.023, 0.126)	0.092 (0.034, 0.161)
	LLaVA 1.5	0.862 (0.782, 0.931)	0.046 (0.011, 0.092)	0.092 (0.034, 0.161)
Clinical	GPT4V	0.441 (0.338, 0.559)	0.074 (0.015, 0.147)	0.485 (0.368, 0.603)
	LLaVA-Med	0.926 (0.853, 0.985)	0.015 (0.000, 0.044)	0.059 (0.015, 0.118)
	LLaVA 1.5	0.647 (0.529, 0.750)	0.088 (0.029, 0.162)	0.265 (0.162, 0.368)
Ancillary Testing	GPT4V	0.448 (0.345, 0.552)	0.115 (0.057, 0.184)	0.437 (0.333, 0.540)
	LLaVA-Med	0.931 (0.874, 0.977)	0.023 (0.000, 0.057)	0.046 (0.011, 0.092)
	LLaVA 1.5	0.690 (0.597, 0.782)	0.115 (0.046, 0.184)	0.195 (0.115, 0.276)

Supplementary Data Table 27: Head-to-head performance of PathChat against other MLLMs on PathQABench open-ended questions as evaluated by pathologist 7, stratified by category. For each open-ended question, 7 pathologists independently ranked the outputs of PathChat and competing models (GPT4V, LLaVA-Med, LLaVA 1.5), blinded to which model produced which response (see **MLLM evaluation** for more details). For each pathologist's evaluation, we compute the win/tie/lose rate of PathChat against said model for the 260 open-ended questions. We report the win/tie/lose rates according to the assessment of pathologist 7. Lose: said model is ranked higher than PathChat; Tie: PathChat is tied with the model in ranking; Win: PathChat is ranked higher than the model. 95% confidence intervals from bootstrapping are included in parentheses. ∗Note that due to guardrails implemented by GPT4V, 38 / 260 queries to the API yielded unsuccessful answers for PathQABench-Public (a maximum number of 3 attempts were made for each question). Regardless, all responses were reviewed by pathologists without special treatment but we also report performance of each model after removing obviously unsuccessful queries for GPT4V in **Supplementary Data Table 56**. For more details see **Evaluation of GPT4V** in **Methods**.

Category	PathChat	GPT4V∗	LLaVA-Med	LLaVA 1.5
Microscopic	0.797 (0.696, 0.884)	0.217 (0.130, 0.319)	0.159 (0.072, 0.246)	0.159 (0.072, 0.246)
Differentiation	0.567 (0.400, 0.733)	0.200 (0.067, 0.333)	0.267 (0.133, 0.433)	0.167 (0.033, 0.300)
Grading	0.529 (0.353, 0.706)	0.235 (0.088, 0.382)	0.382 (0.235, 0.559)	0.265 (0.118, 0.412)
Diagnosis	0.785 (0.684, 0.873)	0.316 (0.215, 0.430)	0.241 (0.152, 0.342)	0.177 (0.101, 0.266)
Risk factors	0.857 (0.571, 1.000)	1.000 (1.000, 1.000)	0.571 (0.143, 0.857)	0.571 (0.143, 1.000)
Prognosis	0.778 (0.644, 0.889)	0.844 (0.733, 0.933)	0.578 (0.422, 0.711)	0.644 (0.489, 0.778)
Treatment	0.826 (0.717, 0.935)	0.957 (0.891, 1.000)	0.522 (0.370, 0.653)	0.696 (0.543, 0.826)
IHC	0.838 (0.703, 0.946)	0.838 (0.703, 0.946)	0.189 (0.081, 0.324)	0.189 (0.081, 0.324)
Molecular	0.763 (0.632, 0.895)	0.947 (0.868, 1.000)	0.368 (0.211, 0.526)	0.632 (0.500, 0.789)
Other Testing	0.900 (0.700, 1.000)	1.000 (1.000, 1.000)	0.700 (0.400, 1.000)	0.800 (0.500, 1.000)

Supplementary Data Table 28: Proportion of open-ended questions in the consensus subset of PathQABench correctly answered by each model, stratified by sub-categories. Two pathologists first independently evaluated the correctness of each model's outputs. Next, after discussion, consensus was reached on 235 of the 260 questions (See **MLLM evaluation** in **Methods** for details). We report the performance on the subset of cases for which a consensus was reached (using the consensus as ground truth). 95% confidence intervals from bootstrapping are included in parentheses. ∗We noted that due to guardrails implemented by GPT4V, 33 / 235 questions of the consensus subset that were submitted to the API yielded unsuccessful answers for PathQABench-Public (a maximum number of 3 attempts were made for each question). However, all responses were reviewed by pathologists and an unsuccessful query was treated as incorrect if the response did not address the question. We also report performance of the models after removing such obviously unsuccessful queries for GPT4V in **Supplementary Data Table 44**. For more details see **Evaluation of GPT4V** in **Methods**.

Category	PathChat	GPT4V∗	LLaVA-Med	LLaVA 1.5
Microscopic	0.781 (0.685, 0.877)	0.247 (0.151, 0.342)	0.164 (0.082, 0.260)	0.164 (0.082, 0.247)
Differentiation	0.594 (0.438, 0.750)	0.188 (0.062, 0.312)	0.250 (0.124, 0.406)	0.156 (0.031, 0.281)
Grading	0.579 (0.421, 0.737)	0.237 (0.105, 0.368)	0.342 (0.211, 0.500)	0.263 (0.132, 0.395)
Diagnosis	0.736 (0.644, 0.816)	0.333 (0.241, 0.437)	0.241 (0.161, 0.333)	0.195 (0.126, 0.276)
Risk factors	0.750 (0.500, 1.000)	1.000 (1.000, 1.000)	0.500 (0.125, 0.875)	0.500 (0.125, 0.875)
Prognosis	0.780 (0.660, 0.880)	0.840 (0.720, 0.940)	0.580 (0.440, 0.720)	0.640 (0.520, 0.760)
Treatment	0.804 (0.686, 0.902)	0.941 (0.863, 1.000)	0.529 (0.392, 0.667)	0.706 (0.569, 0.843)
IHC	0.864 (0.750, 0.955)	0.864 (0.750, 0.955)	0.227 (0.114, 0.341)	0.250 (0.136, 0.386)
Molecular	0.738 (0.619, 0.857)	0.952 (0.881, 1.000)	0.381 (0.238, 0.548)	0.643 (0.500, 0.786)
Other Testing	0.917 (0.750, 1.000)	1.000 (1.000, 1.000)	0.583 (0.333, 0.833)	0.750 (0.500, 1.000)

Supplementary Data Table 29: Proportion of open-ended questions in PathQABench correctly answered by each model as evaluated by pathologist 1, stratified by sub-categories. Two pathologists first independently evaluated the correctness of each model's outputs. Next, after discussion, consensus was reached on 235 of the 260 questions (See **MLLM evaluation** in **Methods** for details). We report the performance according to pathologist 1 (including cases for which a consensus was not ultimately achieved). 95% confidence intervals from bootstrapping are included in parentheses. ∗We noted that due to guardrails implemented by GPT4V, 33 / 235 questions of the consensus subset that were submitted to the API yielded unsuccessful answers for PathQABench-Public (a maximum number of 3 attempts were made for each question). However, all responses were reviewed by pathologists and an unsuccessful query was treated as incorrect if the response did not address the question. We also report performance of the models after removing such obviously unsuccessful queries for GPT4V in **Supplementary Data Table 45**. For more details see **Evaluation of GPT4V** in **Methods**.

Category	PathChat	GPT4V*	LLaVA-Med	LLaVA 1.5
Microscopic	0.781 (0.685, 0.877)	0.247 (0.151, 0.342)	0.178 (0.096, 0.274)	0.151 (0.068, 0.233)
Differentiation	0.531 (0.374, 0.688)	0.188 (0.062, 0.312)	0.250 (0.124, 0.406)	0.156 (0.031, 0.281)
Grading	0.500 (0.368, 0.658)	0.237 (0.105, 0.368)	0.342 (0.211, 0.500)	0.237 (0.105, 0.368)
Diagnosis	0.747 (0.655, 0.839)	0.333 (0.241, 0.437)	0.241 (0.161, 0.333)	0.172 (0.092, 0.253)
Risk factors	0.750 (0.500, 1.000)	0.875 (0.625, 1.000)	0.500 (0.125, 0.875)	0.500 (0.125, 0.875)
Prognosis	0.760 (0.640, 0.880)	0.820 (0.700, 0.920)	0.560 (0.420, 0.680)	0.580 (0.440, 0.700)
Treatment	0.804 (0.686, 0.902)	0.922 (0.843, 0.980)	0.510 (0.373, 0.647)	0.627 (0.490, 0.765)
IHC	0.818 (0.705, 0.932)	0.818 (0.705, 0.932)	0.159 (0.068, 0.273)	0.205 (0.091, 0.341)
Molecular	0.738 (0.619, 0.857)	0.929 (0.857, 1.000)	0.357 (0.214, 0.524)	0.571 (0.429, 0.714)
Other Testing	0.833 (0.583, 1.000)	0.917 (0.750, 1.000)	0.583 (0.333, 0.833)	0.750 (0.500, 1.000)

Supplementary Data Table 30: Proportion of open-ended questions in PathQABench correctly answered by each model as evaluated by pathologist 2, stratified by sub-categories. Two pathologists first independently evaluated the correctness of each model's outputs. Next, after discussion, consensus was reached on 235 of the 260 questions (See **MLLM evaluation** in **Methods** for details). We report the performance according to pathologist 1 (including cases for which a consensus was not ultimately achieved). 95% confidence intervals from bootstrapping are included in parentheses. *We noted that due to guardrails implemented by GPT4V, 33 / 235 questions of the consensus subset that were submitted to the API yielded unsuccessful answers for PathQABench-Public (a maximum number of 3 attempts were made for each question). However, all responses were reviewed by pathologists and an unsuccessful query was treated as incorrect if the response did not address the question. We also report performance of the models after removing such obviously unsuccessful queries for GPT4V in **Supplementary Data Table 46**. For more details see **Evaluation of GPT4V** in **Methods**.

Category	PathChat *vs.* model	Win	Tie	Lose
Microscopic	GPT4V	0.753 (0.685, 0.767)	0.137 (0.014, 0.151)	0.137 (0.096, 0.219)
	LLaVA-Med	0.753 (0.740, 0.795)	0.110 (0.041, 0.178)	0.096 (0.068, 0.110)
	LLaVA 1.5	0.781 (0.767, 0.822)	0.151 (0.068, 0.192)	0.068 (0.041, 0.110)
Differentiation	GPT4V	0.625 (0.562, 0.812)	0.188 (0.031, 0.375)	0.125 (0.062, 0.125)
	LLaVA-Med	0.594 (0.500, 0.688)	0.156 (0.062, 0.375)	0.156 (0.156, 0.188)
	LLaVA 1.5	0.625 (0.594, 0.688)	0.250 (0.062, 0.344)	0.125 (0.094, 0.156)
Grading	GPT4V	0.605 (0.553, 0.711)	0.211 (0.053, 0.342)	0.132 (0.105, 0.184)
	LLaVA-Med	0.579 (0.500, 0.605)	0.237 (0.105, 0.342)	0.184 (0.158, 0.263)
	LLaVA 1.5	0.553 (0.500, 0.579)	0.289 (0.158, 0.316)	0.158 (0.132, 0.184)
Diagnosis	GPT4V	0.713 (0.644, 0.747)	0.149 (0.046, 0.172)	0.138 (0.103, 0.230)
	LLaVA-Med	0.724 (0.701, 0.770)	0.161 (0.092, 0.241)	0.080 (0.069, 0.092)
	LLaVA 1.5	0.759 (0.724, 0.770)	0.149 (0.103, 0.184)	0.092 (0.080, 0.092)
Risk factors	GPT4V	0.375 (0.125, 0.375)	0.125 (0.000, 0.500)	0.500 (0.125, 0.625)
	LLaVA-Med	0.750 (0.625, 0.750)	0.250 (0.125, 0.250)	0.000 (0.000, 0.125)
	LLaVA 1.5	0.500 (0.375, 0.625)	0.250 (0.125, 0.500)	0.250 (0.125, 0.250)
Prognosis	GPT4V	0.400 (0.220, 0.460)	0.220 (0.080, 0.480)	0.380 (0.240, 0.460)
	LLaVA-Med	0.700 (0.660, 0.780)	0.140 (0.080, 0.220)	0.100 (0.080, 0.160)
	LLaVA 1.5	0.520 (0.440, 0.580)	0.200 (0.080, 0.400)	0.260 (0.120, 0.380)
Treatment	GPT4V	0.412 (0.176, 0.490)	0.176 (0.059, 0.471)	0.353 (0.255, 0.451)
	LLaVA-Med	0.784 (0.686, 0.804)	0.098 (0.059, 0.137)	0.098 (0.039, 0.176)
	LLaVA 1.5	0.569 (0.412, 0.608)	0.196 (0.078, 0.392)	0.235 (0.078, 0.353)
IHC	GPT4V	0.409 (0.273, 0.477)	0.273 (0.114, 0.386)	0.341 (0.295, 0.386)
	LLaVA-Med	0.795 (0.750, 0.955)	0.114 (0.023, 0.159)	0.068 (0.023, 0.136)
	LLaVA 1.5	0.727 (0.568, 0.750)	0.182 (0.114, 0.250)	0.091 (0.045, 0.182)
Molecular	GPT4V	0.405 (0.143, 0.405)	0.214 (0.095, 0.476)	0.405 (0.310, 0.500)
	LLaVA-Med	0.786 (0.690, 0.833)	0.119 (0.071, 0.143)	0.095 (0.048, 0.190)
	LLaVA 1.5	0.524 (0.500, 0.619)	0.190 (0.143, 0.381)	0.238 (0.095, 0.333)
Other Testing	GPT4V	0.667 (0.583, 0.667)	0.167 (0.083, 0.333)	0.167 (0.083, 0.250)
	LLaVA-Med	0.917 (0.917, 0.917)	0.000 (0.000, 0.000)	0.083 (0.000, 0.083)
	LLaVA 1.5	0.750 (0.667, 0.833)	0.000 (0.000, 0.250)	0.167 (0.083, 0.250)

Supplementary Data Table 31: Head-to-head performance of PathChat against other MLLMs on PathQABench open-ended questions, stratified by sub-category. For each open-ended question, 7 pathologists independently ranked the outputs of PathChat and competing models (GPT4V, LLaVA-Med, LLaVA 1.5), blinded to which model produced which response (see **MLLM evaluation** for more details). For each pathologist's evaluation, we compute the win/tie/lose rate of PathChat against said model for the 260 open-ended questions. We report the median win/tie/lose rates across the evaluations of 7 pathologists. See **Supplementary Data Table 32–38** for metrics from each individual pathologist. Lose: said model is ranked higher than PathChat; Tie: PathChat is tied with the model in ranking; Win: PathChat is ranked higher than the model. 95% confidence intervals from bootstrapping are included in parentheses. ∗Note that due to guardrails implemented by GPT4V, 38 / 260 queries to the API yielded unsuccessful answers for PathQABench-Public (a maximum number of 3 attempts were made for each question). Regardless, all responses were reviewed by pathologists without special treatment but we also report performance of each model after removing obviously unsuccessful queries for GPT4V in **Supplementary Data Table 57**. For more details see **Evaluation of GPT4V** in **Methods**.

Category	PathChat *vs.* model	Win	Tie	Lose
Microscopic	GPT4V	0.767 (0.671, 0.849)	0.151 (0.068, 0.233)	0.082 (0.027, 0.151)
	LLaVA-Med	0.753 (0.658, 0.849)	0.178 (0.096, 0.260)	0.068 (0.014, 0.137)
	LLaVA 1.5	0.781 (0.685, 0.863)	0.151 (0.068, 0.233)	0.068 (0.014, 0.123)
Differentiation	GPT4V	0.625 (0.438, 0.781)	0.250 (0.125, 0.406)	0.125 (0.031, 0.250)
	LLaVA-Med	0.594 (0.406, 0.750)	0.250 (0.125, 0.406)	0.156 (0.031, 0.281)
	LLaVA 1.5	0.625 (0.438, 0.781)	0.250 (0.094, 0.406)	0.125 (0.031, 0.250)
Grading	GPT4V	0.553 (0.395, 0.711)	0.342 (0.211, 0.500)	0.105 (0.026, 0.211)
	LLaVA-Med	0.500 (0.342, 0.658)	0.342 (0.184, 0.500)	0.158 (0.053, 0.264)
	LLaVA 1.5	0.553 (0.395, 0.711)	0.289 (0.158, 0.447)	0.158 (0.053, 0.289)
Diagnosis	GPT4V	0.747 (0.655, 0.839)	0.149 (0.080, 0.230)	0.103 (0.046, 0.172)
	LLaVA-Med	0.770 (0.678, 0.851)	0.161 (0.092, 0.241)	0.069 (0.023, 0.126)
	LLaVA 1.5	0.736 (0.644, 0.828)	0.172 (0.092, 0.253)	0.092 (0.034, 0.149)
Risk factors	GPT4V	0.625 (0.375, 1.000)	0.250 (0.000, 0.625)	0.125 (0.000, 0.375)
	LLaVA-Med	0.625 (0.375, 1.000)	0.375 (0.000, 0.625)	0.000 (0.000, 0.000)
	LLaVA 1.5	0.375 (0.125, 0.625)	0.500 (0.247, 0.875)	0.125 (0.000, 0.375)
Prognosis	GPT4V	0.460 (0.320, 0.600)	0.300 (0.180, 0.420)	0.240 (0.140, 0.360)
	LLaVA-Med	0.780 (0.660, 0.880)	0.120 (0.040, 0.220)	0.100 (0.020, 0.180)
	LLaVA 1.5	0.440 (0.300, 0.580)	0.300 (0.180, 0.440)	0.260 (0.140, 0.380)
Treatment	GPT4V	0.392 (0.255, 0.529)	0.353 (0.235, 0.490)	0.255 (0.137, 0.373)
	LLaVA-Med	0.804 (0.686, 0.902)	0.098 (0.020, 0.176)	0.098 (0.020, 0.196)
	LLaVA 1.5	0.412 (0.275, 0.549)	0.353 (0.235, 0.471)	0.235 (0.118, 0.353)
IHC	GPT4V	0.409 (0.273, 0.568)	0.273 (0.136, 0.409)	0.318 (0.182, 0.455)
	LLaVA-Med	0.818 (0.705, 0.932)	0.068 (0.000, 0.159)	0.114 (0.023, 0.205)
	LLaVA 1.5	0.773 (0.636, 0.886)	0.182 (0.068, 0.295)	0.045 (0.000, 0.114)
Molecular	GPT4V	0.405 (0.262, 0.548)	0.286 (0.167, 0.429)	0.310 (0.167, 0.452)
	LLaVA-Med	0.833 (0.714, 0.929)	0.071 (0.000, 0.167)	0.095 (0.024, 0.190)
	LLaVA 1.5	0.500 (0.357, 0.667)	0.262 (0.119, 0.381)	0.238 (0.119, 0.357)
Other Testing	GPT4V	0.583 (0.333, 0.833)	0.250 (0.000, 0.500)	0.167 (0.000, 0.417)
	LLaVA-Med	0.917 (0.750, 1.000)	0.000 (0.000, 0.000)	0.083 (0.000, 0.250)
	LLaVA 1.5	0.583 (0.331, 0.833)	0.333 (0.083, 0.583)	0.083 (0.000, 0.250)

Supplementary Data Table 32: Head-to-head performance of PathChat against other MLLMs on PathQABench open-ended questions as evaluated by pathologist 1, stratified by sub-category. For each open-ended question, 7 pathologists independently ranked the outputs of PathChat and competing models (GPT4V, LLaVA-Med, LLaVA 1.5), blinded to which model produced which response (see **MLLM evaluation** for more details). For each pathologist's evaluation, we compute the win/tie/lose rate of PathChat against said model for the 260 open-ended questions. We report the win/tie/lose rates according to the assessment of pathologist 1. Lose: said model is ranked higher than PathChat; Tie: PathChat is tied with the model in ranking; Win: PathChat is ranked higher than the model. 95% confidence intervals from bootstrapping are included in parentheses. ∗Note that due to guardrails implemented by GPT4V, 38 / 260 queries to the API yielded unsuccessful answers for PathQABench-Public (a maximum number of 3 attempts were made for each question). Regardless, all responses were reviewed by pathologists without special treatment but we also report performance of each model after removing obviously unsuccessful queries for GPT4V in **Supplementary Data Table 58**. For more details see **Evaluation of GPT4V** in **Methods**.

Category	PathChat *vs.* model	Win	Tie	Lose
Microscopic	GPT4V	0.685 (0.575, 0.781)	0.178 (0.096, 0.260)	0.137 (0.068, 0.219)
	LLaVA-Med	0.740 (0.630, 0.836)	0.178 (0.096, 0.274)	0.082 (0.027, 0.151)
	LLaVA 1.5	0.767 (0.671, 0.863)	0.192 (0.110, 0.288)	0.041 (0.000, 0.096)
Differentiation	GPT4V	0.469 (0.281, 0.656)	0.438 (0.281, 0.625)	0.094 (0.000, 0.219)
	LLaVA-Med	0.469 (0.312, 0.625)	0.375 (0.219, 0.562)	0.156 (0.062, 0.281)
	LLaVA 1.5	0.500 (0.312, 0.656)	0.344 (0.188, 0.500)	0.156 (0.062, 0.281)
Grading	GPT4V	0.526 (0.368, 0.684)	0.342 (0.184, 0.500)	0.132 (0.026, 0.237)
	LLaVA-Med	0.500 (0.342, 0.658)	0.342 (0.184, 0.500)	0.158 (0.053, 0.289)
	LLaVA 1.5	0.500 (0.342, 0.658)	0.316 (0.184, 0.474)	0.184 (0.079, 0.316)
Diagnosis	GPT4V	0.644 (0.540, 0.736)	0.218 (0.138, 0.310)	0.138 (0.069, 0.207)
	LLaVA-Med	0.701 (0.598, 0.793)	0.241 (0.149, 0.333)	0.057 (0.011, 0.103)
	LLaVA 1.5	0.759 (0.667, 0.839)	0.184 (0.103, 0.276)	0.057 (0.011, 0.103)
Risk factors	GPT4V	0.125 (0.000, 0.375)	0.500 (0.125, 0.875)	0.375 (0.122, 0.750)
	LLaVA-Med	0.625 (0.250, 1.000)	0.250 (0.000, 0.503)	0.125 (0.000, 0.375)
	LLaVA 1.5	0.375 (0.000, 0.750)	0.500 (0.125, 0.875)	0.125 (0.000, 0.375)
Prognosis	GPT4V	0.220 (0.120, 0.340)	0.480 (0.340, 0.620)	0.300 (0.180, 0.440)
	LLaVA-Med	0.720 (0.600, 0.840)	0.180 (0.080, 0.300)	0.100 (0.020, 0.180)
	LLaVA 1.5	0.520 (0.380, 0.660)	0.400 (0.260, 0.540)	0.080 (0.020, 0.160)
Treatment	GPT4V	0.176 (0.078, 0.275)	0.471 (0.333, 0.608)	0.353 (0.235, 0.490)
	LLaVA-Med	0.784 (0.667, 0.882)	0.098 (0.020, 0.176)	0.118 (0.039, 0.216)
	LLaVA 1.5	0.529 (0.412, 0.667)	0.392 (0.255, 0.510)	0.078 (0.020, 0.157)
IHC	GPT4V	0.273 (0.159, 0.409)	0.386 (0.250, 0.545)	0.341 (0.205, 0.477)
	LLaVA-Med	0.795 (0.659, 0.909)	0.136 (0.045, 0.250)	0.068 (0.000, 0.159)
	LLaVA 1.5	0.727 (0.591, 0.841)	0.182 (0.091, 0.295)	0.091 (0.000, 0.182)
Molecular	GPT4V	0.119 (0.024, 0.238)	0.476 (0.333, 0.619)	0.405 (0.262, 0.548)
	LLaVA-Med	0.786 (0.643, 0.905)	0.119 (0.024, 0.238)	0.095 (0.024, 0.190)
	LLaVA 1.5	0.524 (0.357, 0.667)	0.381 (0.238, 0.524)	0.095 (0.024, 0.190)
Other Testing	GPT4V	0.667 (0.417, 0.917)	0.083 (0.000, 0.250)	0.250 (0.000, 0.500)
	LLaVA-Med	0.833 (0.583, 1.000)	0.167 (0.000, 0.417)	0.000 (0.000, 0.000)
	LLaVA 1.5	0.750 (0.500, 1.000)	0.000 (0.000, 0.000)	0.250 (0.000, 0.500)

Supplementary Data Table 33: Head-to-head performance of PathChat against other MLLMs on PathQABench open-ended questions as evaluated by pathologist 2, stratified by sub-category. For each open-ended question, 7 pathologists independently ranked the outputs of PathChat and competing models (GPT4V, LLaVA-Med, LLaVA 1.5), blinded to which model produced which response (see **MLLM evaluation** for more details). For each pathologist's evaluation, we compute the win/tie/lose rate of PathChat against said model for the 260 open-ended questions. We report the win/tie/lose rates according to the assessment of pathologist 2. Lose: said model is ranked higher than PathChat; Tie: PathChat is tied with the model in ranking; Win: PathChat is ranked higher than the model. 95% confidence intervals from bootstrapping are included in parentheses. ∗Note that due to guardrails implemented by GPT4V, 38 / 260 queries to the API yielded unsuccessful answers for PathQABench-Public (a maximum number of 3 attempts were made for each question). Regardless, all responses were reviewed by pathologists without special treatment but we also report performance of each model after removing obviously unsuccessful queries for GPT4V in **Supplementary Data Table 59**. For more details see **Evaluation of GPT4V** in **Methods**.

Category	PathChat *vs.* model	Win	Tie	Lose
Microscopic	GPT4V	0.740 (0.643, 0.836)	0.110 (0.041, 0.192)	0.151 (0.068, 0.233)
	LLaVA-Med	0.781 (0.685, 0.863)	0.110 (0.041, 0.192)	0.110 (0.041, 0.178)
	LLaVA 1.5	0.781 (0.685, 0.877)	0.151 (0.082, 0.233)	0.068 (0.014, 0.137)
Differentiation	GPT4V	0.688 (0.531, 0.844)	0.188 (0.062, 0.344)	0.125 (0.031, 0.250)
	LLaVA-Med	0.656 (0.500, 0.812)	0.156 (0.031, 0.281)	0.188 (0.062, 0.312)
	LLaVA 1.5	0.625 (0.438, 0.781)	0.250 (0.125, 0.406)	0.125 (0.031, 0.250)
Grading	GPT4V	0.605 (0.447, 0.737)	0.211 (0.105, 0.342)	0.184 (0.079, 0.316)
	LLaVA-Med	0.605 (0.447, 0.763)	0.132 (0.053, 0.237)	0.263 (0.132, 0.395)
	LLaVA 1.5	0.553 (0.395, 0.711)	0.263 (0.132, 0.421)	0.184 (0.053, 0.316)
Diagnosis	GPT4V	0.713 (0.621, 0.793)	0.172 (0.092, 0.253)	0.115 (0.057, 0.184)
	LLaVA-Med	0.759 (0.667, 0.839)	0.149 (0.080, 0.230)	0.092 (0.034, 0.161)
	LLaVA 1.5	0.770 (0.678, 0.851)	0.149 (0.080, 0.230)	0.080 (0.034, 0.138)
Risk factors	GPT4V	0.375 (0.000, 0.750)	0.000 (0.000, 0.000)	0.625 (0.250, 1.000)
	LLaVA-Med	0.750 (0.500, 1.000)	0.250 (0.000, 0.500)	0.000 (0.000, 0.000)
	LLaVA 1.5	0.625 (0.250, 0.875)	0.125 (0.000, 0.375)	0.250 (0.000, 0.625)
Prognosis	GPT4V	0.460 (0.320, 0.600)	0.080 (0.020, 0.160)	0.460 (0.340, 0.600)
	LLaVA-Med	0.700 (0.560, 0.820)	0.140 (0.040, 0.240)	0.160 (0.060, 0.260)
	LLaVA 1.5	0.520 (0.380, 0.660)	0.080 (0.020, 0.160)	0.400 (0.280, 0.540)
Treatment	GPT4V	0.471 (0.333, 0.608)	0.039 (0.000, 0.098)	0.490 (0.353, 0.647)
	LLaVA-Med	0.784 (0.667, 0.882)	0.137 (0.059, 0.235)	0.078 (0.020, 0.157)
	LLaVA 1.5	0.569 (0.431, 0.706)	0.078 (0.020, 0.157)	0.353 (0.216, 0.490)
IHC	GPT4V	0.432 (0.295, 0.591)	0.182 (0.068, 0.296)	0.386 (0.249, 0.545)
	LLaVA-Med	0.750 (0.614, 0.886)	0.114 (0.023, 0.227)	0.136 (0.045, 0.250)
	LLaVA 1.5	0.727 (0.591, 0.841)	0.205 (0.091, 0.318)	0.068 (0.000, 0.159)
Molecular	GPT4V	0.405 (0.262, 0.548)	0.095 (0.024, 0.190)	0.500 (0.357, 0.667)
	LLaVA-Med	0.786 (0.667, 0.905)	0.143 (0.048, 0.262)	0.071 (0.000, 0.167)
	LLaVA 1.5	0.524 (0.381, 0.667)	0.143 (0.048, 0.262)	0.333 (0.190, 0.476)
Other Testing	GPT4V	0.583 (0.333, 0.833)	0.083 (0.000, 0.250)	0.333 (0.083, 0.583)
	LLaVA-Med	0.917 (0.750, 1.000)	0.000 (0.000, 0.000)	0.083 (0.000, 0.250)
	LLaVA 1.5	0.833 (0.583, 1.000)	0.000 (0.000, 0.000)	0.167 (0.000, 0.417)

Supplementary Data Table 34: Head-to-head performance of PathChat against other MLLMs on PathQABench open-ended questions as evaluated by pathologist 3, stratified by sub-category. For each open-ended question, 7 pathologists independently ranked the outputs of PathChat and competing models (GPT4V, LLaVA-Med, LLaVA 1.5), blinded to which model produced which response (see **MLLM evaluation** for more details). For each pathologist's evaluation, we compute the win/tie/lose rate of PathChat against said model for the 260 open-ended questions. We report the win/tie/lose rates according to the assessment of pathologist 3. Lose: said model is ranked higher than PathChat; Tie: PathChat is tied with the model in ranking; Win: PathChat is ranked higher than the model. 95% confidence intervals from bootstrapping are included in parentheses. ∗Note that due to guardrails implemented by GPT4V, 38 / 260 queries to the API yielded unsuccessful answers for PathQABench-Public (a maximum number of 3 attempts were made for each question). Regardless, all responses were reviewed by pathologists without special treatment but we also report performance of each model after removing obviously unsuccessful queries for GPT4V in **Supplementary Data Table 60**. For more details see **Evaluation of GPT4V** in **Methods**.

Category	PathChat *vs.* model	Win	Tie	Lose
Microscopic	GPT4V	0.753 (0.658, 0.849)	0.137 (0.055, 0.219)	0.110 (0.041, 0.178)
	LLaVA-Med	0.753 (0.658, 0.849)	0.178 (0.096, 0.260)	0.068 (0.014, 0.123)
	LLaVA 1.5	0.767 (0.671, 0.863)	0.205 (0.110, 0.301)	0.027 (0.000, 0.068)
Differentiation	GPT4V	0.594 (0.406, 0.750)	0.375 (0.219, 0.531)	0.031 (0.000, 0.094)
	LLaVA-Med	0.500 (0.344, 0.688)	0.406 (0.219, 0.562)	0.094 (0.000, 0.219)
	LLaVA 1.5	0.594 (0.406, 0.750)	0.344 (0.188, 0.531)	0.062 (0.000, 0.156)
Grading	GPT4V	0.605 (0.447, 0.763)	0.316 (0.158, 0.474)	0.079 (0.000, 0.184)
	LLaVA-Med	0.553 (0.395, 0.711)	0.316 (0.158, 0.474)	0.132 (0.026, 0.237)
	LLaVA 1.5	0.579 (0.421, 0.737)	0.395 (0.237, 0.553)	0.026 (0.000, 0.079)
Diagnosis	GPT4V	0.667 (0.563, 0.759)	0.126 (0.057, 0.207)	0.207 (0.126, 0.287)
	LLaVA-Med	0.667 (0.563, 0.770)	0.253 (0.161, 0.356)	0.080 (0.034, 0.138)
	LLaVA 1.5	0.724 (0.621, 0.816)	0.195 (0.115, 0.276)	0.080 (0.023, 0.138)
Risk factors	GPT4V	0.375 (0.125, 0.750)	0.125 (0.000, 0.375)	0.500 (0.125, 0.875)
	LLaVA-Med	0.750 (0.500, 1.000)	0.000 (0.000, 0.000)	0.250 (0.000, 0.500)
	LLaVA 1.5	0.625 (0.250, 0.875)	0.125 (0.000, 0.375)	0.250 (0.000, 0.625)
Prognosis	GPT4V	0.400 (0.280, 0.540)	0.220 (0.120, 0.340)	0.380 (0.240, 0.520)
	LLaVA-Med	0.660 (0.520, 0.800)	0.220 (0.120, 0.340)	0.120 (0.040, 0.220)
	LLaVA 1.5	0.580 (0.440, 0.720)	0.200 (0.100, 0.300)	0.220 (0.100, 0.340)
Treatment	GPT4V	0.412 (0.275, 0.549)	0.176 (0.078, 0.275)	0.412 (0.294, 0.549)
	LLaVA-Med	0.686 (0.569, 0.804)	0.137 (0.059, 0.235)	0.176 (0.078, 0.294)
	LLaVA 1.5	0.608 (0.490, 0.745)	0.196 (0.098, 0.295)	0.196 (0.098, 0.314)
IHC	GPT4V	0.341 (0.205, 0.500)	0.318 (0.182, 0.455)	0.341 (0.205, 0.477)
	LLaVA-Med	0.591 (0.432, 0.728)	0.273 (0.159, 0.409)	0.136 (0.045, 0.250)
	LLaVA 1.5	0.568 (0.432, 0.705)	0.250 (0.136, 0.386)	0.182 (0.068, 0.295)
Molecular	GPT4V	0.381 (0.238, 0.524)	0.214 (0.095, 0.333)	0.405 (0.262, 0.548)
	LLaVA-Med	0.690 (0.548, 0.833)	0.119 (0.024, 0.214)	0.190 (0.071, 0.310)
	LLaVA 1.5	0.619 (0.476, 0.762)	0.190 (0.071, 0.310)	0.190 (0.071, 0.310)
Other Testing	GPT4V	0.750 (0.500, 0.917)	0.167 (0.000, 0.417)	0.083 (0.000, 0.250)
	LLaVA-Med	0.917 (0.750, 1.000)	0.000 (0.000, 0.000)	0.083 (0.000, 0.250)
	LLaVA 1.5	0.833 (0.583, 1.000)	0.000 (0.000, 0.000)	0.167 (0.000, 0.417)

Supplementary Data Table 35: Head-to-head performance of PathChat against other MLLMs on PathQABench open-ended questions as evaluated by pathologist 4, stratified by sub-category. For each open-ended question, 7 pathologists independently ranked the outputs of PathChat and competing models (GPT4V, LLaVA-Med, LLaVA 1.5), blinded to which model produced which response (see **MLLM evaluation** for more details). For each pathologist's evaluation, we compute the win/tie/lose rate of PathChat against said model for the 260 open-ended questions. We report the win/tie/lose rates according to the assessment of pathologist 4. Lose: said model is ranked higher than PathChat; Tie: PathChat is tied with the model in ranking; Win: PathChat is ranked higher than the model. 95% confidence intervals from bootstrapping are included in parentheses. $*$Note that due to guardrails implemented by GPT4V, 38 / 260 queries to the API yielded unsuccessful answers for PathQABench-Public (a maximum number of 3 attempts were made for each question). Regardless, all responses were reviewed by pathologists without special treatment but we also report performance of each model after removing obviously unsuccessful queries for GPT4V in **Supplementary Data Table 61**. For more details see **Evaluation of GPT4V** in **Methods**.

Category	PathChat *vs.* model	Win	Tie	Lose
Microscopic	GPT4V	0.767 (0.671, 0.863)	0.137 (0.068, 0.219)	0.096 (0.041, 0.178)
	LLaVA-Med	0.795 (0.699, 0.877)	0.110 (0.041, 0.178)	0.096 (0.027, 0.165)
	LLaVA 1.5	0.822 (0.726, 0.904)	0.068 (0.014, 0.137)	0.110 (0.041, 0.178)
Differentiation	GPT4V	0.812 (0.688, 0.938)	0.125 (0.031, 0.250)	0.062 (0.000, 0.188)
	LLaVA-Med	0.688 (0.531, 0.844)	0.156 (0.031, 0.281)	0.156 (0.031, 0.281)
	LLaVA 1.5	0.688 (0.531, 0.844)	0.219 (0.094, 0.375)	0.094 (0.000, 0.188)
Grading	GPT4V	0.711 (0.553, 0.842)	0.158 (0.053, 0.289)	0.132 (0.026, 0.263)
	LLaVA-Med	0.579 (0.421, 0.737)	0.237 (0.105, 0.368)	0.184 (0.079, 0.316)
	LLaVA 1.5	0.553 (0.395, 0.711)	0.316 (0.184, 0.474)	0.132 (0.026, 0.237)
Diagnosis	GPT4V	0.782 (0.690, 0.862)	0.149 (0.080, 0.230)	0.069 (0.023, 0.126)
	LLaVA-Med	0.713 (0.621, 0.805)	0.207 (0.126, 0.288)	0.080 (0.023, 0.149)
	LLaVA 1.5	0.759 (0.655, 0.851)	0.149 (0.080, 0.219)	0.092 (0.034, 0.161)
Risk factors	GPT4V	0.125 (0.000, 0.375)	0.875 (0.625, 1.000)	0.000 (0.000, 0.000)
	LLaVA-Med	0.750 (0.500, 1.000)	0.250 (0.000, 0.500)	0.000 (0.000, 0.000)
	LLaVA 1.5	0.250 (0.000, 0.500)	0.750 (0.500, 1.000)	0.000 (0.000, 0.000)
Prognosis	GPT4V	0.180 (0.080, 0.280)	0.720 (0.600, 0.840)	0.100 (0.020, 0.200)
	LLaVA-Med	0.580 (0.440, 0.720)	0.360 (0.240, 0.500)	0.060 (0.000, 0.120)
	LLaVA 1.5	0.440 (0.320, 0.580)	0.440 (0.300, 0.580)	0.120 (0.040, 0.220)
Treatment	GPT4V	0.157 (0.059, 0.255)	0.784 (0.667, 0.882)	0.059 (0.000, 0.118)
	LLaVA-Med	0.686 (0.549, 0.804)	0.294 (0.176, 0.431)	0.020 (0.000, 0.059)
	LLaVA 1.5	0.392 (0.255, 0.529)	0.529 (0.392, 0.667)	0.078 (0.020, 0.157)
IHC	GPT4V	0.273 (0.136, 0.409)	0.705 (0.568, 0.841)	0.023 (0.000, 0.068)
	LLaVA-Med	0.795 (0.659, 0.909)	0.159 (0.068, 0.273)	0.045 (0.000, 0.114)
	LLaVA 1.5	0.477 (0.341, 0.614)	0.523 (0.386, 0.659)	0.000 (0.000, 0.000)
Molecular	GPT4V	0.143 (0.048, 0.262)	0.762 (0.642, 0.881)	0.095 (0.024, 0.190)
	LLaVA-Med	0.690 (0.548, 0.833)	0.310 (0.167, 0.452)	0.000 (0.000, 0.000)
	LLaVA 1.5	0.357 (0.214, 0.500)	0.548 (0.381, 0.690)	0.095 (0.024, 0.190)
Other Testing	GPT4V	0.333 (0.083, 0.583)	0.583 (0.250, 0.833)	0.083 (0.000, 0.250)
	LLaVA-Med	0.917 (0.750, 1.000)	0.000 (0.000, 0.000)	0.083 (0.000, 0.250)
	LLaVA 1.5	0.667 (0.417, 0.917)	0.250 (0.000, 0.500)	0.083 (0.000, 0.250)

Supplementary Data Table 36: Head-to-head performance of PathChat against other MLLMs on PathQABench open-ended questions as evaluated by pathologist 5, stratified by sub-category. For each open-ended question, 7 pathologists independently ranked the outputs of PathChat and competing models (GPT4V, LLaVA-Med, LLaVA 1.5), blinded to which model produced which response (see **MLLM evaluation** for more details). For each pathologist's evaluation, we compute the win/tie/lose rate of PathChat against said model for the 260 open-ended questions. We report the win/tie/lose rates according to the assessment of pathologist 5. Lose: said model is ranked higher than PathChat; Tie: PathChat is tied with the model in ranking; Win: PathChat is ranked higher than the model. 95% confidence intervals from bootstrapping are included in parentheses. ∗Note that due to guardrails implemented by GPT4V, 38 / 260 queries to the API yielded unsuccessful answers for PathQABench-Public (a maximum number of 3 attempts were made for each question). Regardless, all responses were reviewed by pathologists without special treatment but we also report performance of each model after removing obviously unsuccessful queries for GPT4V in **Supplementary Data Table 62**. For more details see **Evaluation of GPT4V** in **Methods**.

Category	PathChat *vs.* model	Win	Tie	Lose
Microscopic	GPT4V	0.767 (0.658, 0.863)	0.014 (0.000, 0.041)	0.219 (0.137, 0.315)
	LLaVA-Med	0.712 (0.603, 0.808)	0.041 (0.000, 0.096)	0.247 (0.151, 0.356)
	LLaVA 1.5	0.726 (0.616, 0.822)	0.096 (0.041, 0.164)	0.178 (0.096, 0.274)
Differentiation	GPT4V	0.844 (0.688, 0.969)	0.031 (0.000, 0.094)	0.125 (0.031, 0.250)
	LLaVA-Med	0.562 (0.406, 0.719)	0.062 (0.000, 0.156)	0.375 (0.219, 0.562)
	LLaVA 1.5	0.656 (0.469, 0.812)	0.031 (0.000, 0.094)	0.312 (0.156, 0.469)
Grading	GPT4V	0.789 (0.658, 0.895)	0.053 (0.000, 0.132)	0.158 (0.053, 0.289)
	LLaVA-Med	0.579 (0.421, 0.737)	0.105 (0.026, 0.211)	0.316 (0.184, 0.474)
	LLaVA 1.5	0.500 (0.342, 0.658)	0.158 (0.053, 0.289)	0.342 (0.210, 0.500)
Diagnosis	GPT4V	0.747 (0.655, 0.839)	0.023 (0.000, 0.057)	0.230 (0.138, 0.322)
	LLaVA-Med	0.724 (0.632, 0.816)	0.092 (0.034, 0.149)	0.184 (0.103, 0.276)
	LLaVA 1.5	0.690 (0.586, 0.782)	0.103 (0.046, 0.172)	0.207 (0.126, 0.299)
Risk factors	GPT4V	0.375 (0.000, 0.750)	0.000 (0.000, 0.000)	0.625 (0.250, 1.000)
	LLaVA-Med	0.625 (0.250, 0.875)	0.250 (0.000, 0.500)	0.125 (0.000, 0.375)
	LLaVA 1.5	0.500 (0.125, 0.750)	0.250 (0.000, 0.625)	0.250 (0.000, 0.625)
Prognosis	GPT4V	0.480 (0.340, 0.600)	0.120 (0.040, 0.220)	0.400 (0.280, 0.540)
	LLaVA-Med	0.660 (0.520, 0.800)	0.080 (0.020, 0.160)	0.260 (0.140, 0.380)
	LLaVA 1.5	0.560 (0.420, 0.700)	0.060 (0.000, 0.120)	0.380 (0.240, 0.500)
Treatment	GPT4V	0.529 (0.392, 0.667)	0.118 (0.039, 0.216)	0.353 (0.235, 0.490)
	LLaVA-Med	0.706 (0.588, 0.824)	0.059 (0.000, 0.118)	0.235 (0.137, 0.353)
	LLaVA 1.5	0.569 (0.431, 0.687)	0.059 (0.000, 0.137)	0.373 (0.255, 0.490)
IHC	GPT4V	0.591 (0.455, 0.727)	0.114 (0.023, 0.205)	0.295 (0.159, 0.432)
	LLaVA-Med	0.977 (0.932, 1.000)	0.023 (0.000, 0.068)	0.000 (0.000, 0.000)
	LLaVA 1.5	0.727 (0.591, 0.841)	0.068 (0.000, 0.159)	0.205 (0.091, 0.318)
Molecular	GPT4V	0.476 (0.333, 0.643)	0.143 (0.048, 0.262)	0.381 (0.238, 0.524)
	LLaVA-Med	0.738 (0.595, 0.857)	0.071 (0.000, 0.167)	0.190 (0.071, 0.310)
	LLaVA 1.5	0.595 (0.452, 0.762)	0.024 (0.000, 0.071)	0.381 (0.238, 0.524)
Other Testing	GPT4V	0.667 (0.417, 0.917)	0.333 (0.083, 0.583)	0.000 (0.000, 0.000)
	LLaVA-Med	1.000 (1.000, 1.000)	0.000 (0.000, 0.000)	0.000 (0.000, 0.000)
	LLaVA 1.5	0.750 (0.500, 0.919)	0.083 (0.000, 0.250)	0.167 (0.000, 0.417)

Supplementary Data Table 37: Head-to-head performance of PathChat against other MLLMs on PathQABench open-ended questions as evaluated by pathologist 6, stratified by sub-category. For each open-ended question, 7 pathologists independently ranked the outputs of PathChat and competing models (GPT4V, LLaVA-Med, LLaVA 1.5), blinded to which model produced which response (see **MLLM evaluation** for more details). For each pathologist's evaluation, we compute the win/tie/lose rate of PathChat against said model for the 260 open-ended questions. We report the win/tie/lose rates according to the assessment of pathologist 6. Lose: said model is ranked higher than PathChat; Tie: PathChat is tied with the model in ranking; Win: PathChat is ranked higher than the model. 95% confidence intervals from bootstrapping are included in parentheses. $*$Note that due to guardrails implemented by GPT4V, 38 / 260 queries to the API yielded unsuccessful answers for PathQABench-Public (a maximum number of 3 attempts were made for each question). Regardless, all responses were reviewed by pathologists without special treatment but we also report performance of each model after removing obviously unsuccessful queries for GPT4V in **Supplementary Data Table 63**. For more details see **Evaluation of GPT4V** in **Methods**.

Category	PathChat *vs.* model	Win	Tie	Lose
Microscopic	GPT4V	0.548 (0.425, 0.658)	0.014 (0.000, 0.041)	0.438 (0.329, 0.548)
	LLaVA-Med	0.863 (0.794, 0.932)	0.041 (0.000, 0.096)	0.096 (0.041, 0.164)
	LLaVA 1.5	0.890 (0.808, 0.959)	0.041 (0.000, 0.096)	0.068 (0.014, 0.124)
Differentiation	GPT4V	0.562 (0.406, 0.750)	0.031 (0.000, 0.094)	0.406 (0.249, 0.562)
	LLaVA-Med	0.750 (0.594, 0.875)	0.062 (0.000, 0.156)	0.188 (0.062, 0.344)
	LLaVA 1.5	0.812 (0.656, 0.938)	0.062 (0.000, 0.156)	0.125 (0.031, 0.250)
Grading	GPT4V	0.632 (0.474, 0.789)	0.026 (0.000, 0.079)	0.342 (0.184, 0.500)
	LLaVA-Med	0.737 (0.579, 0.868)	0.053 (0.000, 0.132)	0.211 (0.079, 0.342)
	LLaVA 1.5	0.842 (0.737, 0.947)	0.000 (0.000, 0.000)	0.158 (0.053, 0.263)
Diagnosis	GPT4V	0.552 (0.448, 0.644)	0.046 (0.011, 0.092)	0.402 (0.299, 0.517)
	LLaVA-Med	0.839 (0.770, 0.920)	0.069 (0.023, 0.126)	0.092 (0.034, 0.161)
	LLaVA 1.5	0.862 (0.782, 0.931)	0.046 (0.011, 0.092)	0.092 (0.034, 0.161)
Risk factors	GPT4V	0.125 (0.000, 0.375)	0.125 (0.000, 0.375)	0.750 (0.500, 1.000)
	LLaVA-Med	0.875 (0.625, 1.000)	0.125 (0.000, 0.375)	0.000 (0.000, 0.000)
	LLaVA 1.5	0.500 (0.125, 0.875)	0.000 (0.000, 0.000)	0.500 (0.125, 0.875)
Prognosis	GPT4V	0.400 (0.280, 0.540)	0.080 (0.020, 0.160)	0.520 (0.380, 0.660)
	LLaVA-Med	0.920 (0.840, 0.980)	0.000 (0.000, 0.000)	0.080 (0.020, 0.160)
	LLaVA 1.5	0.600 (0.460, 0.740)	0.100 (0.020, 0.200)	0.300 (0.180, 0.420)
Treatment	GPT4V	0.490 (0.353, 0.627)	0.059 (0.000, 0.118)	0.451 (0.314, 0.588)
	LLaVA-Med	0.961 (0.902, 1.000)	0.000 (0.000, 0.000)	0.039 (0.000, 0.098)
	LLaVA 1.5	0.647 (0.510, 0.765)	0.098 (0.020, 0.196)	0.255 (0.137, 0.373)
IHC	GPT4V	0.477 (0.341, 0.614)	0.114 (0.023, 0.205)	0.409 (0.273, 0.545)
	LLaVA-Med	0.955 (0.886, 1.000)	0.023 (0.000, 0.068)	0.023 (0.000, 0.068)
	LLaVA 1.5	0.750 (0.614, 0.886)	0.114 (0.045, 0.205)	0.136 (0.045, 0.250)
Molecular	GPT4V	0.405 (0.262, 0.548)	0.071 (0.000, 0.143)	0.524 (0.357, 0.667)
	LLaVA-Med	0.929 (0.833, 1.000)	0.024 (0.000, 0.071)	0.048 (0.000, 0.119)
	LLaVA 1.5	0.619 (0.476, 0.762)	0.143 (0.048, 0.262)	0.238 (0.119, 0.381)
Other Testing	GPT4V	0.667 (0.417, 0.917)	0.167 (0.000, 0.417)	0.167 (0.000, 0.417)
	LLaVA-Med	0.917 (0.750, 1.000)	0.000 (0.000, 0.000)	0.083 (0.000, 0.250)
	LLaVA 1.5	0.750 (0.500, 1.000)	0.000 (0.000, 0.000)	0.250 (0.000, 0.500)

Supplementary Data Table 38: Head-to-head performance of PathChat against other MLLMs on PathQABench open-ended questions as evaluated by pathologist 7, stratified by sub-category. For each open-ended question, 7 pathologists independently ranked the outputs of PathChat and competing models (GPT4V, LLaVA-Med, LLaVA 1.5), blinded to which model produced which response (see **MLLM evaluation** for more details). For each pathologist's evaluation, we compute the win/tie/lose rate of PathChat against said model for the 260 open-ended questions. We report the win/tie/lose rates according to the assessment of pathologist 7. Lose: said model is ranked higher than PathChat; Tie: PathChat is tied with the model in ranking; Win: PathChat is ranked higher than the model. 95% confidence intervals from bootstrapping are included in parentheses. ∗Note that due to guardrails implemented by GPT4V, 38 / 260 queries to the API yielded unsuccessful answers for PathQABench-Public (a maximum number of 3 attempts were made for each question). Regardless, all responses were reviewed by pathologists without special treatment but we also report performance of each model after removing obviously unsuccessful queries for GPT4V in **Supplementary Data Table 64**. For more details see **Evaluation of GPT4V** in **Methods**.

Broad category	Count	Sub-category	Count
Microscopy	78 / 109	Microscopic Description	53 / 73
		Differentiation	22 / 32
		Grading	23 / 38
Diagnosis	74 / 87	Diagnosis	74 / 87
Clinical	64 / 68	Risk Factors	8 / 8
		Prognosis	48 / 50
		Treatment	49 / 51
Ancillary Testing	86 / 87	IHC	37 / 44
		Molecular	41 / 42
		Other Testing	12 / 12

Supplementary Data Table 39: Broad and sub-categories for PathQABench open-ended questions succesfully answered by GPT4V Of the 260 open-ended questions in PathQABench, GPT4V successfully answered 222. Each question may fall under more than one category. For each category, we indicate how many of the total questions were successfully answered by GPT4V within a maximum of 3 attempts (see **Evaluation of GPT4V** of **Methods**).

Model	Consensus ($n = 202$)	Pathologist 1 ($n = 222$)	Pathologist 2 ($n = 222$)
PathChat	0.812 (0.757, 0.861)	0.802 (0.752, 0.851)	0.788 (0.734, 0.838)
GPT4V	0.599 (0.530, 0.663)	0.622 (0.550, 0.685)	0.608 (0.541, 0.671)
LLaVA-Med	0.307 (0.247, 0.371)	0.311 (0.252, 0.369)	0.297 (0.239, 0.351)
LLaVA 1.5	0.317 (0.248, 0.381)	0.333 (0.270, 0.396)	0.297 (0.239, 0.356)

Supplementary Data Table 40: Proportion of open-ended questions in PathQABench correctly answered by each model, restricted to successful GPT4V queries. Two pathologists first independently evaluated the correctness of each model's outputs. Next, after discussion, consensus was reached on 235 of the 260 questions (See **MLLM evaluation** in **Methods** for details). We report the performance on both the subset of cases for which a consensus was reached (using the consensus as ground truth) and also the performance according to each pathologist (including cases for which a consensus was not ultimately achieved). 95% confidence intervals from bootstrapping are included in parentheses. See **Supplementary Data Table 41–46** for accuracy stratified by category and subcategory. Due to guardrails implemented by GPT4V, 38 / 260 questions in total and 33 / 235 questions of the consensus subset that were submitted to the API yielded unsuccessful answers for PathQABench-Public (a maximum number of 3 attempts were made for each question). However, all responses were reviewed by pathologists and an unsuccessful query was treated as incorrect if the response did not address the question. We report performance of the models after removing such obviously unsuccessful queries for GPT4V here. For more details see **Evaluation of GPT4V** in **Methods**.

Category	PathChat	GPT4V	LLaVA-Med	LLaVA 1.5
Microscopy	0.764 (0.667, 0.861)	0.306 (0.208, 0.417)	0.194 (0.111, 0.292)	0.181 (0.097, 0.264)
Diagnosis	0.809 (0.706, 0.897)	0.353 (0.250, 0.456)	0.235 (0.147, 0.338)	0.191 (0.103, 0.279)
Clinical	0.828 (0.724, 0.914)	0.914 (0.845, 0.983)	0.500 (0.379, 0.621)	0.638 (0.517, 0.759)
Ancillary Testing	0.813 (0.720, 0.893)	0.893 (0.813, 0.960)	0.347 (0.240, 0.453)	0.440 (0.333, 0.547)

Supplementary Data Table 41: Proportion of open-ended questions in the consensus subset of PathQABench correctly answered by each model, stratified by broad categories and restricted to successful GPT4V queries. Two pathologists first independently evaluated the correctness of each model's outputs. Next, after discussion, consensus was reached on 235 of the 260 questions (See **MLLM evaluation** in **Methods** for details). We report the performance on the subset of cases for which a consensus was reached (using the consensus as ground truth). 95% confidence intervals from bootstrapping are included in parentheses. Due to guardrails implemented by GPT4V, 33 / 235 questions of the consensus subset that were submitted to the API yielded unsuccessful answers for PathQABench-Public (a maximum number of 3 attempts were made for each question). However, all responses were reviewed by pathologists and an unsuccessful query was treated as incorrect if the response did not address the question. We report performance of the models after removing such obviously unsuccessful queries for GPT4V here ($n = 202$). For more details see **Evaluation of GPT4V** in **Methods**.

Category	PathChat	GPT4V	LLaVA-Med	LLaVA 1.5
Microscopy	0.756 (0.654, 0.846)	0.333 (0.231, 0.436)	0.192 (0.115, 0.282)	0.192 (0.103, 0.282)
Diagnosis	0.770 (0.662, 0.865)	0.378 (0.270, 0.486)	0.230 (0.135, 0.311)	0.203 (0.108, 0.297)
Clinical	0.812 (0.719, 0.906)	0.906 (0.828, 0.969)	0.500 (0.391, 0.625)	0.641 (0.516, 0.766)
Ancillary Testing	0.814 (0.721, 0.895)	0.907 (0.837, 0.965)	0.360 (0.267, 0.465)	0.465 (0.372, 0.570)

Supplementary Data Table 42: Proportion of open-ended questions in PathQABench correctly answered by each model as evaluated by pathologist 1, stratified by broad categories and restricted to successful GPT4V queries. Two pathologists first independently evaluated the correctness of each model's outputs. Next, after discussion, consensus was reached on 235 of the 260 questions (See **MLLM evaluation** in **Methods** for details). We report the performance according to pathologist 1 (including cases for which a consensus was not ultimately achieved). 95% confidence intervals from bootstrapping are included in parentheses. Due to guardrails implemented by GPT4V, 38 / 260 questions submitted to the API yielded unsuccessful answers for PathQABench-Public (a maximum number of 3 attempts were made for each question). However, all responses were reviewed by pathologists and an unsuccessful query was treated as incorrect if the response did not address the question. We report performance of the models after removing such obviously unsuccessful queries for GPT4V here ($n = 222$). For more details see **Evaluation of GPT4V** in **Methods**.

Category	PathChat	GPT4V	LLaVA-Med	LLaVA 1.5
Microscopy	0.744 (0.641, 0.833)	0.333 (0.231, 0.436)	0.205 (0.115, 0.295)	0.167 (0.077, 0.244)
Diagnosis	0.770 (0.675, 0.865)	0.378 (0.270, 0.486)	0.230 (0.135, 0.311)	0.176 (0.095, 0.270)
Clinical	0.797 (0.688, 0.891)	0.891 (0.812, 0.953)	0.484 (0.375, 0.609)	0.578 (0.469, 0.688)
Ancillary Testing	0.791 (0.698, 0.872)	0.872 (0.802, 0.942)	0.314 (0.221, 0.419)	0.407 (0.314, 0.500)

Supplementary Data Table 43: Proportion of open-ended questions in PathQABench correctly answered by each model as evaluated by pathologist 2, stratified by broad categories and restricted to successful GPT4V queries. Two pathologists first independently evaluated the correctness of each model's outputs. Next, after discussion, consensus was reached on 235 of the 260 questions (See **MLLM evaluation** in **Methods** for details). We report the performance according to pathologist 2 (including cases for which a consensus was not ultimately achieved). 95% confidence intervals from bootstrapping are included in parentheses. Due to guardrails implemented by GPT4V, 38 / 260 questions that were submitted to the API yielded unsuccessful answers for PathQABench-Public (a maximum number of 3 attempts were made for each question). However, all responses were reviewed by pathologists and an unsuccessful query was treated as incorrect if the response did not address the question. We report performance of the models after removing such obviously unsuccessful queries for GPT4V here ($n = 222$). For more details see **Evaluation of GPT4V** in **Methods**.

Category	PathChat	GPT4V	LLaVA-Med	LLaVA 1.5
Microscopic	0.816 (0.694, 0.918)	0.306 (0.184, 0.449)	0.143 (0.041, 0.245)	0.143 (0.061, 0.245)
Differentiation	0.636 (0.455, 0.818)	0.273 (0.091, 0.456)	0.227 (0.091, 0.409)	0.182 (0.045, 0.364)
Grading	0.571 (0.380, 0.762)	0.333 (0.143, 0.524)	0.333 (0.143, 0.525)	0.286 (0.095, 0.476)
Diagnosis	0.809 (0.706, 0.897)	0.353 (0.250, 0.456)	0.235 (0.147, 0.338)	0.191 (0.103, 0.279)
Risk factors	0.857 (0.571, 1.000)	1.000 (1.000, 1.000)	0.571 (0.143, 0.857)	0.571 (0.143, 1.000)
Prognosis	0.791 (0.651, 0.907)	0.884 (0.791, 0.953)	0.581 (0.442, 0.721)	0.674 (0.535, 0.814)
Treatment	0.844 (0.733, 0.933)	0.956 (0.889, 1.000)	0.511 (0.377, 0.644)	0.689 (0.556, 0.822)
IHC	0.838 (0.703, 0.946)	0.838 (0.703, 0.946)	0.189 (0.081, 0.324)	0.189 (0.081, 0.324)
Molecular	0.784 (0.649, 0.919)	0.946 (0.865, 1.000)	0.351 (0.189, 0.514)	0.622 (0.459, 0.784)
Other Testing	0.900 (0.700, 1.000)	1.000 (1.000, 1.000)	0.700 (0.400, 1.000)	0.800 (0.500, 1.000)

Supplementary Data Table 44: Proportion of open-ended questions in the consensus subset of PathQABench correctly answered by each model, stratified by sub-categories and restricted to successful GPT4V queries. Two pathologists first independently evaluated the correctness of each model's outputs. Next, after discussion, consensus was reached on 235 of the 260 questions (See **MLLM evaluation** in **Methods** for details). We report the performance on the subset of cases for which a consensus was reached (using the consensus as ground truth). 95% confidence intervals from bootstrapping are included in parentheses. Due to guardrails implemented by GPT4V, 33 / 235 questions of the consensus subset that were submitted to the API yielded unsuccessful answers for PathQABench-Public (a maximum number of 3 attempts were made for each question). However, all responses were reviewed by pathologists and an unsuccessful query was treated as incorrect if the response did not address the question. We report performance of the models after removing such obviously unsuccessful queries for GPT4V here. For more details see **Evaluation of GPT4V** in **Methods**.

Category	PathChat	GPT4V	LLaVA-Med	LLaVA 1.5
Microscopic	0.792 (0.679, 0.887)	0.340 (0.208, 0.472)	0.151 (0.057, 0.264)	0.208 (0.094, 0.321)
Differentiation	0.636 (0.455, 0.818)	0.409 (0.227, 0.636)	0.182 (0.045, 0.364)	0.182 (0.045, 0.364)
Grading	0.609 (0.435, 0.783)	0.435 (0.217, 0.609)	0.261 (0.087, 0.435)	0.304 (0.130, 0.522)
Diagnosis	0.770 (0.662, 0.865)	0.378 (0.270, 0.486)	0.230 (0.135, 0.311)	0.176 (0.094, 0.270)
Risk factors	0.750 (0.500, 1.000)	1.000 (1.000, 1.000)	0.500 (0.125, 0.875)	0.500 (0.125, 0.875)
Prognosis	0.812 (0.688, 0.917)	0.896 (0.792, 0.958)	0.583 (0.438, 0.729)	0.667 (0.542, 0.792)
Treatment	0.837 (0.714, 0.939)	0.959 (0.898, 1.000)	0.510 (0.388, 0.653)	0.694 (0.571, 0.817)
IHC	0.864 (0.750, 0.955)	0.864 (0.750, 0.955)	0.273 (0.159, 0.409)	0.250 (0.136, 0.386)
Molecular	0.780 (0.659, 0.902)	0.951 (0.878, 1.000)	0.366 (0.220, 0.512)	0.634 (0.488, 0.780)
Other Testing	0.917 (0.750, 1.000)	1.000 (1.000, 1.000)	0.667 (0.417, 0.917)	0.750 (0.500, 1.000)

Supplementary Data Table 45: Proportion of open-ended questions in PathQABench correctly answered by each model as evaluated by pathologist 1, stratified by sub-categories and restricted to successful GPT4V queries. Two pathologists first independently evaluated the correctness of each model's outputs. Next, after discussion, consensus was reached on 235 of the 260 questions (See **MLLM evaluation** in **Methods** for details). We report the performance according to pathologist 1 (including cases for which a consensus was not ultimately achieved). 95% confidence intervals from bootstrapping are included in parentheses. Due to guardrails implemented by GPT4V, 33 / 235 questions of the consensus subset that were submitted to the API yielded unsuccessful answers for PathQABench-Public (a maximum number of 3 attempts were made for each question). However, all responses were reviewed by pathologists and an unsuccessful query was treated as incorrect if the response did not address the question. We report performance of the models after removing such obviously unsuccessful queries for GPT4V here. For more details see **Evaluation of GPT4V** in **Methods**.

Category	PathChat	GPT4V	LLaVA-Med	LLaVA 1.5
Microscopic	0.792 (0.679, 0.887)	0.340 (0.208, 0.472)	0.170 (0.075, 0.283)	0.132 (0.056, 0.226)
Differentiation	0.636 (0.455, 0.818)	0.273 (0.091, 0.456)	0.227 (0.091, 0.409)	0.182 (0.045, 0.364)
Grading	0.565 (0.348, 0.740)	0.348 (0.174, 0.522)	0.304 (0.130, 0.522)	0.261 (0.087, 0.435)
Diagnosis	0.770 (0.675, 0.865)	0.378 (0.270, 0.486)	0.230 (0.135, 0.311)	0.176 (0.095, 0.270)
Risk factors	0.750 (0.500, 1.000)	0.875 (0.625, 1.000)	0.500 (0.125, 0.875)	0.500 (0.125, 0.875)
Prognosis	0.771 (0.646, 0.875)	0.854 (0.750, 0.938)	0.562 (0.417, 0.688)	0.604 (0.479, 0.729)
Treatment	0.816 (0.714, 0.918)	0.939 (0.857, 1.000)	0.490 (0.367, 0.633)	0.633 (0.490, 0.756)
IHC	0.818 (0.705, 0.932)	0.818 (0.705, 0.932)	0.159 (0.068, 0.273)	0.205 (0.091, 0.341)
Molecular	0.756 (0.610, 0.878)	0.927 (0.829, 1.000)	0.341 (0.195, 0.488)	0.561 (0.415, 0.707)
Other Testing	0.833 (0.583, 1.000)	0.917 (0.750, 1.000)	0.583 (0.333, 0.833)	0.750 (0.500, 1.000)

Supplementary Data Table 46: Proportion of open-ended questions in PathQABench correctly answered by each model as evaluated by pathologist 2, stratified by sub-categories and restricted to successful GPT4V queries. Two pathologists first independently evaluated the correctness of each model's outputs. Next, after discussion, consensus was reached on 235 of the 260 questions (See **MLLM evaluation** in **Methods** for details). We report the performance according to pathologist 1 (including cases for which a consensus was not ultimately achieved). 95% confidence intervals from bootstrapping are included in parentheses. Due to guardrails implemented by GPT4V, 33 / 235 questions of the consensus subset that were submitted to the API yielded unsuccessful answers for PathQABench-Public (a maximum number of 3 attempts were made for each question). However, all responses were reviewed by pathologists and an unsuccessful query was treated as incorrect if the response did not address the question. We report performance of the models after removing such obviously unsuccessful queries for GPT4V here. For more details see **Evaluation of GPT4V** in **Methods**.

PathChat *vs.* model	Win	Tie	Lose
GPT4V	0.532 (0.459, 0.595)	0.216 (0.081, 0.311)	0.252 (0.189, 0.311)
LLaVA-Med	0.761 (0.712, 0.770)	0.144 (0.068, 0.212)	0.086 (0.072, 0.117)
LLaVA 1.5	0.685 (0.658, 0.712)	0.216 (0.086, 0.225)	0.126 (0.072, 0.158)

Supplementary Data Table 47: Head-to-head performance of PathChat against other MLLMs on PathQABench open-ended questions, restricted to successful GPT4V queries. For each open-ended question, 7 pathologists independently ranked the outputs of PathChat and competing models (GPT4V, LLaVA-Med, LLaVA 1.5), blinded to which model produced which response (see **MLLM evaluation** for more details). For each pathologist's evaluation, we compute the win/tie/lose rate of PathChat against said model for the 260 open-ended questions. We report the median win/tie/lose rates across the evaluations of 7 pathologists. See **Supplementary Data Table 48** for metrics from each individual pathologist. Lose: said model is ranked higher than PathChat; Tie: PathChat is tied with the model in ranking; Win: PathChat is ranked higher than the model. 95% confidence intervals from bootstrapping are included in parentheses. Due to guardrails implemented by GPT4V, 38 / 260 queries to the API yielded unsuccessful answers for PathQABench-Public (a maximum number of 3 attempts were made for each question). We report performance of each model after removing obviously unsuccessful queries for GPT4V here. For more details see **Evaluation of GPT4V** in **Methods**.

Pathologist	PathChat *vs.* model	Win	Tie	Lose
1	GPT4V	0.595 (0.532, 0.658)	0.216 (0.167, 0.275)	0.189 (0.140, 0.243)
	LLaVA-Med	0.770 (0.716, 0.820)	0.144 (0.104, 0.189)	0.086 (0.054, 0.126)
	LLaVA 1.5	0.671 (0.608, 0.730)	0.221 (0.167, 0.275)	0.108 (0.068, 0.149)
2	GPT4V	0.459 (0.392, 0.527)	0.311 (0.257, 0.374)	0.230 (0.176, 0.293)
	LLaVA-Med	0.757 (0.698, 0.811)	0.162 (0.113, 0.207)	0.081 (0.050, 0.122)
	LLaVA 1.5	0.712 (0.649, 0.770)	0.225 (0.171, 0.284)	0.063 (0.036, 0.095)
3	GPT4V	0.577 (0.509, 0.635)	0.113 (0.077, 0.153)	0.311 (0.257, 0.374)
	LLaVA-Med	0.761 (0.703, 0.820)	0.122 (0.081, 0.167)	0.117 (0.081, 0.162)
	LLaVA 1.5	0.703 (0.640, 0.761)	0.140 (0.095, 0.189)	0.158 (0.113, 0.207)
4	GPT4V	0.532 (0.464, 0.595)	0.216 (0.167, 0.275)	0.252 (0.198, 0.315)
	LLaVA-Med	0.671 (0.608, 0.730)	0.212 (0.158, 0.270)	0.117 (0.077, 0.158)
	LLaVA 1.5	0.658 (0.595, 0.716)	0.216 (0.162, 0.270)	0.126 (0.085, 0.171)
5	GPT4V	0.473 (0.405, 0.545)	0.455 (0.392, 0.523)	0.072 (0.041, 0.108)
	LLaVA-Med	0.712 (0.653, 0.775)	0.216 (0.158, 0.270)	0.072 (0.040, 0.113)
	LLaVA 1.5	0.595 (0.523, 0.662)	0.333 (0.275, 0.396)	0.072 (0.041, 0.113)
6	GPT4V	0.644 (0.581, 0.707)	0.081 (0.045, 0.117)	0.275 (0.221, 0.333)
	LLaVA-Med	0.761 (0.703, 0.815)	0.068 (0.036, 0.104)	0.171 (0.122, 0.225)
	LLaVA 1.5	0.685 (0.626, 0.739)	0.086 (0.050, 0.126)	0.230 (0.176, 0.288)
7	GPT4V	0.432 (0.365, 0.495)	0.063 (0.032, 0.095)	0.505 (0.437, 0.572)
	LLaVA-Med	0.883 (0.842, 0.923)	0.050 (0.023, 0.081)	0.068 (0.041, 0.099)
	LLaVA 1.5	0.770 (0.716, 0.824)	0.077 (0.045, 0.113)	0.153 (0.108, 0.203)

Supplementary Data Table 48: Head-to-head performance of PathChat against other MLLMs on PathQABench open-ended questions as evaluated by 7 individual pathologists, restricted to successful GPT4V queries. For each open-ended question, 7 pathologists independently ranked the outputs of PathChat and competing models (GPT4V, LLaVA-Med, LLaVA 1.5), blinded to which model produced which response (see **MLLM evaluation** for more details). For each pathologist's evaluation, we compute the win/tie/lose rate of PathChat against said model for the 260 questions. We report the win/tie/lose rates for each pathologist. Lose: said model is ranked higher than PathChat; Tie: PathChat is tied with the model in ranking; Win: PathChat is ranked higher than the model. 95% confidence intervals from bootstrapping are included in parentheses. For more details see **PathChat model evaluation** in **Methods**. See **Supplementary Data Table 47** for aggregated metrics across all 7 pathologists. Due to guardrails implemented by GPT4V, 38 / 260 queries to the API yielded unsuccessful answers for PathQABench-Public (a maximum number of 3 attempts were made for each question). We report performance of each model after removing obviously unsuccessful queries for GPT4V here. For more details see **Evaluation of GPT4V** in **Methods**.

Category	PathChat *vs.* model	Win	Tie	Lose
Microscopy	GPT4V	0.679 (0.641, 0.692)	0.167 (0.038, 0.218)	0.167 (0.115, 0.244)
	LLaVA-Med	0.718 (0.667, 0.769)	0.167 (0.077, 0.205)	0.115 (0.090, 0.141)
	LLaVA 1.5	0.731 (0.692, 0.756)	0.154 (0.090, 0.192)	0.103 (0.064, 0.115)
Diagnosis	GPT4V	0.703 (0.622, 0.757)	0.149 (0.054, 0.176)	0.135 (0.108, 0.230)
	LLaVA-Med	0.743 (0.730, 0.784)	0.162 (0.081, 0.216)	0.081 (0.054, 0.095)
	LLaVA 1.5	0.743 (0.703, 0.784)	0.162 (0.108, 0.176)	0.081 (0.081, 0.095)
Clinical	GPT4V	0.422 (0.219, 0.469)	0.219 (0.078, 0.453)	0.344 (0.234, 0.500)
	LLaVA-Med	0.766 (0.688, 0.812)	0.125 (0.094, 0.172)	0.094 (0.031, 0.141)
	LLaVA 1.5	0.578 (0.484, 0.625)	0.188 (0.078, 0.359)	0.203 (0.094, 0.312)
Ancillary Testing	GPT4V	0.442 (0.256, 0.453)	0.244 (0.128, 0.395)	0.349 (0.291, 0.419)
	LLaVA-Med	0.791 (0.744, 0.872)	0.128 (0.035, 0.186)	0.093 (0.047, 0.105)
	LLaVA 1.5	0.640 (0.616, 0.674)	0.198 (0.116, 0.256)	0.186 (0.105, 0.198)

Supplementary Data Table 49: Head-to-head performance of PathChat against other MLLMs on PathQABench open-ended questions, stratified by category and restricted to successful GPT4V categories. For each open-ended question, 7 pathologists independently ranked the outputs of PathChat and competing models (GPT4V, LLaVA-Med, LLaVA 1.5), blinded to which model produced which response (see **MLLM evaluation** for more details). For each pathologist's evaluation, we compute the win/tie/lose rate of PathChat against said model for the 260 open-ended questions. We report the median win/tie/lose rates across the evaluations of 7 pathologists. See **Supplementary Data Table 50–56** for metrics from each individual pathologist. Lose: said model is ranked higher than PathChat; Tie: PathChat is tied with the model in ranking; Win: PathChat is ranked higher than the model. 95% confidence intervals from bootstrapping are included in parentheses. Due to guardrails implemented by GPT4V, 38 / 260 queries to the API yielded unsuccessful answers for PathQABench-Public (a maximum number of 3 attempts were made for each question). We report performance of each model after removing obviously unsuccessful queries for GPT4V here. For more details see **Evaluation of GPT4V** in **Methods**.

Category	PathChat *vs.* model	Win	Tie	Lose
Microscopy	GPT4V	0.679 (0.577, 0.782)	0.167 (0.090, 0.256)	0.154 (0.077, 0.244)
	LLaVA-Med	0.705 (0.615, 0.795)	0.205 (0.115, 0.308)	0.090 (0.038, 0.154)
	LLaVA 1.5	0.705 (0.603, 0.795)	0.192 (0.103, 0.282)	0.103 (0.038, 0.167)
Diagnosis	GPT4V	0.757 (0.662, 0.851)	0.135 (0.068, 0.216)	0.108 (0.041, 0.176)
	LLaVA-Med	0.784 (0.689, 0.865)	0.162 (0.081, 0.243)	0.054 (0.014, 0.108)
	LLaVA 1.5	0.743 (0.649, 0.838)	0.176 (0.095, 0.270)	0.081 (0.027, 0.149)
Clinical	GPT4V	0.469 (0.359, 0.594)	0.297 (0.188, 0.391)	0.234 (0.141, 0.344)
	LLaVA-Med	0.812 (0.719, 0.906)	0.125 (0.047, 0.203)	0.062 (0.016, 0.125)
	LLaVA 1.5	0.484 (0.359, 0.609)	0.312 (0.203, 0.422)	0.203 (0.109, 0.312)
Ancillary Testing	GPT4V	0.453 (0.349, 0.547)	0.256 (0.163, 0.349)	0.291 (0.198, 0.384)
	LLaVA-Med	0.826 (0.744, 0.907)	0.070 (0.023, 0.128)	0.105 (0.047, 0.174)
	LLaVA 1.5	0.640 (0.546, 0.744)	0.221 (0.140, 0.314)	0.140 (0.081, 0.209)

Supplementary Data Table 50: Head-to-head performance of PathChat against other MLLMs on PathQABench open-ended questions as evaluated by pathologist 1, stratified by category and restricted to successful GPT4V queries. For each open-ended question, 7 pathologists independently ranked the outputs of PathChat and competing models (GPT4V, LLaVA-Med, LLaVA 1.5), blinded to which model produced which response (see **MLLM evaluation** for more details). For each pathologist's evaluation, we compute the win/tie/lose rate of PathChat against said model for the 260 open-ended questions. We report the win/tie/lose rates according to the assessment of pathologist 1. Lose: said model is ranked higher than PathChat; Tie: PathChat is tied with the model in ranking; Win: PathChat is ranked higher than the model. 95% confidence intervals from bootstrapping are included in parentheses. Due to guardrails implemented by GPT4V, 38 / 260 queries to the API yielded unsuccessful answers for PathQABench-Public (a maximum number of 3 attempts were made for each question). We report performance of each model after removing obviously unsuccessful queries for GPT4V here. For more details see **Evaluation of GPT4V** in **Methods**.

Category	PathChat *vs.* model	Win	Tie	Lose
Microscopy	GPT4V	0.641 (0.538, 0.744)	0.192 (0.115, 0.282)	0.167 (0.090, 0.256)
	LLaVA-Med	0.718 (0.615, 0.808)	0.167 (0.090, 0.244)	0.115 (0.051, 0.192)
	LLaVA 1.5	0.756 (0.667, 0.846)	0.179 (0.103, 0.256)	0.064 (0.013, 0.115)
Diagnosis	GPT4V	0.662 (0.554, 0.757)	0.216 (0.135, 0.311)	0.122 (0.041, 0.203)
	LLaVA-Med	0.743 (0.635, 0.838)	0.216 (0.135, 0.311)	0.041 (0.000, 0.095)
	LLaVA 1.5	0.784 (0.676, 0.865)	0.162 (0.081, 0.243)	0.054 (0.014, 0.108)
Clinical	GPT4V	0.219 (0.125, 0.328)	0.453 (0.328, 0.578)	0.328 (0.219, 0.438)
	LLaVA-Med	0.766 (0.656, 0.859)	0.141 (0.062, 0.234)	0.094 (0.031, 0.172)
	LLaVA 1.5	0.547 (0.422, 0.656)	0.359 (0.250, 0.469)	0.094 (0.031, 0.172)
Ancillary Testing	GPT4V	0.256 (0.174, 0.349)	0.395 (0.291, 0.500)	0.349 (0.256, 0.453)
	LLaVA-Med	0.791 (0.698, 0.872)	0.140 (0.070, 0.221)	0.070 (0.023, 0.128)
	LLaVA 1.5	0.640 (0.535, 0.733)	0.256 (0.163, 0.349)	0.105 (0.047, 0.163)

Supplementary Data Table 51: Head-to-head performance of PathChat against other MLLMs on PathQABench open-ended questions as evaluated by pathologist 2, stratified by category and restricted to successful GPT4V queries. For each open-ended question, 7 pathologists independently ranked the outputs of PathChat and competing models (GPT4V, LLaVA-Med, LLaVA 1.5), blinded to which model produced which response (see **MLLM evaluation** for more details). For each pathologist's evaluation, we compute the win/tie/lose rate of PathChat against said model for the 260 open-ended questions. We report the win/tie/lose rates according to the assessment of pathologist 2. Lose: said model is ranked higher than PathChat; Tie: PathChat is tied with the model in ranking; Win: PathChat is ranked higher than the model. 95% confidence intervals from bootstrapping are included in parentheses. Due to guardrails implemented by GPT4V, 38 / 260 queries to the API yielded unsuccessful answers for PathQABench-Public (a maximum number of 3 attempts were made for each question). We report performance of each model after removing obviously unsuccessful queries for GPT4V here. For more details see **Evaluation of GPT4V** in **Methods**.

Category	PathChat *vs.* model	Win	Tie	Lose
Microscopy	GPT4V	0.692 (0.590, 0.795)	0.103 (0.038, 0.179)	0.205 (0.115, 0.295)
	LLaVA-Med	0.769 (0.679, 0.859)	0.090 (0.026, 0.154)	0.141 (0.064, 0.231)
	LLaVA 1.5	0.744 (0.654, 0.846)	0.154 (0.077, 0.244)	0.103 (0.038, 0.179)
Diagnosis	GPT4V	0.703 (0.595, 0.797)	0.162 (0.081, 0.257)	0.135 (0.068, 0.216)
	LLaVA-Med	0.757 (0.649, 0.851)	0.149 (0.068, 0.243)	0.095 (0.027, 0.176)
	LLaVA 1.5	0.770 (0.662, 0.851)	0.149 (0.068, 0.230)	0.081 (0.027, 0.149)
Clinical	GPT4V	0.453 (0.328, 0.578)	0.047 (0.000, 0.109)	0.500 (0.359, 0.625)
	LLaVA-Med	0.766 (0.672, 0.859)	0.125 (0.047, 0.219)	0.109 (0.031, 0.188)
	LLaVA 1.5	0.578 (0.453, 0.703)	0.078 (0.016, 0.156)	0.344 (0.234, 0.469)
Ancillary Testing	GPT4V	0.453 (0.349, 0.547)	0.128 (0.058, 0.198)	0.419 (0.326, 0.512)
	LLaVA-Med	0.767 (0.674, 0.849)	0.128 (0.070, 0.198)	0.105 (0.047, 0.163)
	LLaVA 1.5	0.651 (0.558, 0.756)	0.151 (0.081, 0.221)	0.198 (0.116, 0.279)

Supplementary Data Table 52: Head-to-head performance of PathChat against other MLLMs on PathQABench open-ended questions as evaluated by pathologist 3, stratified by category and restricted to successful GPT4V queries. For each open-ended question, 7 pathologists independently ranked the outputs of PathChat and competing models (GPT4V, LLaVA-Med, LLaVA 1.5), blinded to which model produced which response (see **MLLM evaluation** for more details). For each pathologist's evaluation, we compute the win/tie/lose rate of PathChat against said model for the 260 open-ended questions. We report the win/tie/lose rates according to the assessment of pathologist 3. Lose: said model is ranked higher than PathChat; Tie: PathChat is tied with the model in ranking; Win: PathChat is ranked higher than the model. 95% confidence intervals from bootstrapping are included in parentheses. Due to guardrails implemented by GPT4V, 38 / 260 queries to the API yielded unsuccessful answers for PathQABench-Public (a maximum number of 3 attempts were made for each question). We report performance of each model after removing obviously unsuccessful queries for GPT4V here. For more details see **Evaluation of GPT4V** in **Methods**.

Category	PathChat *vs.* model	Win	Tie	Lose
Microscopy	GPT4V	0.667 (0.564, 0.782)	0.218 (0.128, 0.308)	0.115 (0.051, 0.192)
	LLaVA-Med	0.654 (0.551, 0.756)	0.244 (0.154, 0.346)	0.103 (0.038, 0.179)
	LLaVA 1.5	0.692 (0.590, 0.782)	0.244 (0.154, 0.333)	0.064 (0.013, 0.115)
Diagnosis	GPT4V	0.622 (0.514, 0.717)	0.149 (0.068, 0.230)	0.230 (0.135, 0.324)
	LLaVA-Med	0.662 (0.554, 0.770)	0.257 (0.162, 0.351)	0.081 (0.027, 0.149)
	LLaVA 1.5	0.689 (0.581, 0.784)	0.230 (0.135, 0.324)	0.081 (0.027, 0.149)
Clinical	GPT4V	0.406 (0.281, 0.516)	0.219 (0.125, 0.328)	0.375 (0.266, 0.484)
	LLaVA-Med	0.688 (0.578, 0.797)	0.172 (0.094, 0.266)	0.141 (0.062, 0.234)
	LLaVA 1.5	0.625 (0.500, 0.735)	0.188 (0.094, 0.281)	0.188 (0.094, 0.281)
Ancillary Testing	GPT4V	0.407 (0.314, 0.523)	0.244 (0.151, 0.337)	0.349 (0.244, 0.442)
	LLaVA-Med	0.663 (0.558, 0.756)	0.186 (0.105, 0.279)	0.151 (0.081, 0.233)
	LLaVA 1.5	0.616 (0.512, 0.721)	0.198 (0.116, 0.291)	0.186 (0.105, 0.267)

Supplementary Data Table 53: Head-to-head performance of PathChat against other MLLMs on PathQABench open-ended questions as evaluated by pathologist 4, stratified by category and restricted to successful GPT4V queries. For each open-ended question, 7 pathologists independently ranked the outputs of PathChat and competing models (GPT4V, LLaVA-Med, LLaVA 1.5), blinded to which model produced which response (see **MLLM evaluation** for more details). For each pathologist's evaluation, we compute the win/tie/lose rate of PathChat against said model for the 260 open-ended questions. We report the win/tie/lose rates according to the assessment of pathologist 4. Lose: said model is ranked higher than PathChat; Tie: PathChat is tied with the model in ranking; Win: PathChat is ranked higher than the model. 95% confidence intervals from bootstrapping are included in parentheses. Due to guardrails implemented by GPT4V, 38 / 260 queries to the API yielded unsuccessful answers for PathQABench-Public (a maximum number of 3 attempts were made for each question). We report performance of each model after removing obviously unsuccessful queries for GPT4V here. For more details see **Evaluation of GPT4V** in **Methods**.

Category	PathChat *vs.* model	Win	Tie	Lose
Microscopy	GPT4V	0.679 (0.590, 0.782)	0.218 (0.141, 0.308)	0.103 (0.038, 0.167)
	LLaVA-Med	0.718 (0.615, 0.808)	0.167 (0.090, 0.244)	0.115 (0.051, 0.192)
	LLaVA 1.5	0.731 (0.628, 0.833)	0.154 (0.077, 0.231)	0.115 (0.051, 0.192)
Diagnosis	GPT4V	0.757 (0.662, 0.838)	0.176 (0.095, 0.270)	0.068 (0.014, 0.135)
	LLaVA-Med	0.730 (0.622, 0.824)	0.189 (0.108, 0.284)	0.081 (0.027, 0.149)
	LLaVA 1.5	0.730 (0.635, 0.824)	0.176 (0.095, 0.270)	0.095 (0.027, 0.162)
Clinical	GPT4V	0.156 (0.062, 0.250)	0.766 (0.656, 0.859)	0.078 (0.016, 0.141)
	LLaVA-Med	0.625 (0.516, 0.734)	0.344 (0.234, 0.453)	0.031 (0.000, 0.078)
	LLaVA 1.5	0.422 (0.312, 0.547)	0.500 (0.375, 0.609)	0.078 (0.016, 0.156)
Ancillary Testing	GPT4V	0.209 (0.128, 0.291)	0.721 (0.628, 0.826)	0.070 (0.023, 0.128)
	LLaVA-Med	0.744 (0.651, 0.826)	0.221 (0.128, 0.314)	0.035 (0.000, 0.081)
	LLaVA 1.5	0.419 (0.314, 0.512)	0.523 (0.430, 0.628)	0.058 (0.012, 0.105)

Supplementary Data Table 54: Head-to-head performance of PathChat against other MLLMs on PathQABench open-ended questions as evaluated by pathologist 5, stratified by category and restricted to successful GPT4V queries. For each open-ended question, 7 pathologists independently ranked the outputs of PathChat and competing models (GPT4V, LLaVA-Med, LLaVA 1.5), blinded to which model produced which response (see **MLLM evaluation** for more details). For each pathologist's evaluation, we compute the win/tie/lose rate of PathChat against said model for the 260 open-ended questions. We report the win/tie/lose rates according to the assessment of pathologist 5. Lose: said model is ranked higher than PathChat; Tie: PathChat is tied with the model in ranking; Win: PathChat is ranked higher than the model. 95% confidence intervals from bootstrapping are included in parentheses. Due to guardrails implemented by GPT4V, 38 / 260 queries to the API yielded unsuccessful answers for PathQABench-Public (a maximum number of 3 attempts were made for each question). We report performance of each model after removing obviously unsuccessful queries for GPT4V here. For more details see **Evaluation of GPT4V** in **Methods**.

Category	PathChat *vs.* model	Win	Tie	Lose
Microscopy	GPT4V	0.718 (0.615, 0.821)	0.038 (0.000, 0.077)	0.244 (0.154, 0.333)
	LLaVA-Med	0.667 (0.564, 0.769)	0.077 (0.026, 0.141)	0.256 (0.167, 0.359)
	LLaVA 1.5	0.692 (0.590, 0.782)	0.090 (0.038, 0.154)	0.218 (0.128, 0.321)
Diagnosis	GPT4V	0.743 (0.649, 0.838)	0.027 (0.000, 0.068)	0.230 (0.149, 0.324)
	LLaVA-Med	0.743 (0.649, 0.838)	0.081 (0.027, 0.149)	0.176 (0.095, 0.257)
	LLaVA 1.5	0.703 (0.608, 0.797)	0.108 (0.041, 0.189)	0.189 (0.108, 0.284)
Clinical	GPT4V	0.531 (0.406, 0.656)	0.125 (0.047, 0.203)	0.344 (0.234, 0.469)
	LLaVA-Med	0.703 (0.594, 0.812)	0.094 (0.031, 0.172)	0.203 (0.094, 0.312)
	LLaVA 1.5	0.609 (0.484, 0.734)	0.078 (0.016, 0.156)	0.312 (0.203, 0.438)
Ancillary Testing	GPT4V	0.558 (0.453, 0.663)	0.140 (0.070, 0.221)	0.302 (0.209, 0.395)
	LLaVA-Med	0.872 (0.791, 0.942)	0.035 (0.000, 0.081)	0.093 (0.035, 0.163)
	LLaVA 1.5	0.674 (0.570, 0.767)	0.047 (0.012, 0.093)	0.279 (0.198, 0.384)

Supplementary Data Table 55: Head-to-head performance of PathChat against other MLLMs on PathQABench open-ended questions as evaluated by pathologist 6, stratified by category and restricted to successful GPT4V queries. For each open-ended question, 7 pathologists independently ranked the outputs of PathChat and competing models (GPT4V, LLaVA-Med, LLaVA 1.5), blinded to which model produced which response (see **MLLM evaluation** for more details). For each pathologist's evaluation, we compute the win/tie/lose rate of PathChat against said model for the 260 open-ended questions. We report the win/tie/lose rates according to the assessment of pathologist 6. Lose: said model is ranked higher than PathChat; Tie: PathChat is tied with the model in ranking; Win: PathChat is ranked higher than the model. 95% confidence intervals from bootstrapping are included in parentheses. Due to guardrails implemented by GPT4V, 38 / 260 queries to the API yielded unsuccessful answers for PathQABench-Public (a maximum number of 3 attempts were made for each question). We report performance of each model after removing obviously unsuccessful queries for GPT4V here. For more details see **Evaluation of GPT4V** in **Methods**.

Category	PathChat *vs.* model	Win	Tie	Lose
Microscopy	GPT4V	0.436 (0.321, 0.538)	0.013 (0.000, 0.038)	0.551 (0.448, 0.667)
	LLaVA-Med	0.846 (0.756, 0.923)	0.064 (0.013, 0.115)	0.090 (0.026, 0.154)
	LLaVA 1.5	0.833 (0.744, 0.923)	0.064 (0.013, 0.115)	0.103 (0.038, 0.167)
Diagnosis	GPT4V	0.486 (0.378, 0.608)	0.054 (0.014, 0.108)	0.459 (0.338, 0.581)
	LLaVA-Med	0.838 (0.757, 0.905)	0.081 (0.027, 0.149)	0.081 (0.027, 0.149)
	LLaVA 1.5	0.851 (0.770, 0.919)	0.054 (0.014, 0.108)	0.095 (0.041, 0.162)
Clinical	GPT4V	0.422 (0.312, 0.547)	0.078 (0.016, 0.156)	0.500 (0.375, 0.609)
	LLaVA-Med	0.953 (0.891, 1.000)	0.016 (0.000, 0.047)	0.031 (0.000, 0.078)
	LLaVA 1.5	0.641 (0.516, 0.750)	0.094 (0.031, 0.172)	0.266 (0.156, 0.375)
Ancillary Testing	GPT4V	0.442 (0.337, 0.547)	0.116 (0.058, 0.186)	0.442 (0.337, 0.535)
	LLaVA-Med	0.930 (0.872, 0.977)	0.023 (0.000, 0.058)	0.047 (0.012, 0.093)
	LLaVA 1.5	0.686 (0.581, 0.779)	0.116 (0.047, 0.186)	0.198 (0.116, 0.291)

Supplementary Data Table 56: Head-to-head performance of PathChat against other MLLMs on PathQABench open-ended questions as evaluated by pathologist 7, stratified by category and restricted to successful GPT4V queries. For each open-ended question, 7 pathologists independently ranked the outputs of PathChat and competing models (GPT4V, LLaVA-Med, LLaVA 1.5), blinded to which model produced which response (see **MLLM evaluation** for more details). For each pathologist's evaluation, we compute the win/tie/lose rate of PathChat against said model for the 260 open-ended questions. We report the win/tie/lose rates according to the assessment of pathologist 7. Lose: said model is ranked higher than PathChat; Tie: PathChat is tied with the model in ranking; Win: PathChat is ranked higher than the model. 95% confidence intervals from bootstrapping are included in parentheses. Due to guardrails implemented by GPT4V, 38 / 260 queries to the API yielded unsuccessful answers for PathQABench-Public (a maximum number of 3 attempts were made for each question). We report performance of each model after removing obviously unsuccessful queries for GPT4V here. For more details see **Evaluation of GPT4V** in **Methods**.

Category	PathChat *vs.* model	Win	Tie	Lose
Microscopic	GPT4V	0.717 (0.698, 0.717)	0.151 (0.019, 0.151)	0.151 (0.113, 0.264)
	LLaVA-Med	0.755 (0.736, 0.811)	0.113 (0.057, 0.189)	0.113 (0.075, 0.113)
	LLaVA 1.5	0.792 (0.755, 0.811)	0.132 (0.075, 0.151)	0.075 (0.038, 0.113)
Differentiation	GPT4V	0.636 (0.500, 0.727)	0.182 (0.045, 0.364)	0.182 (0.091, 0.182)
	LLaVA-Med	0.636 (0.545, 0.682)	0.182 (0.091, 0.273)	0.136 (0.091, 0.227)
	LLaVA 1.5	0.636 (0.636, 0.682)	0.273 (0.091, 0.273)	0.091 (0.091, 0.091)
Grading	GPT4V	0.565 (0.522, 0.609)	0.261 (0.087, 0.261)	0.217 (0.174, 0.304)
	LLaVA-Med	0.609 (0.565, 0.652)	0.217 (0.087, 0.304)	0.174 (0.087, 0.217)
	LLaVA 1.5	0.565 (0.522, 0.565)	0.261 (0.130, 0.348)	0.174 (0.087, 0.174)
Diagnosis	GPT4V	0.703 (0.622, 0.757)	0.149 (0.054, 0.176)	0.135 (0.108, 0.230)
	LLaVA-Med	0.743 (0.730, 0.784)	0.162 (0.081, 0.216)	0.081 (0.054, 0.095)
	LLaVA 1.5	0.743 (0.703, 0.784)	0.162 (0.108, 0.176)	0.081 (0.081, 0.095)
Risk factors	GPT4V	0.375 (0.125, 0.375)	0.125 (0.000, 0.500)	0.500 (0.125, 0.625)
	LLaVA-Med	0.750 (0.625, 0.750)	0.250 (0.125, 0.250)	0.000 (0.000, 0.125)
	LLaVA 1.5	0.500 (0.375, 0.625)	0.250 (0.125, 0.500)	0.250 (0.125, 0.250)
Prognosis	GPT4V	0.396 (0.208, 0.458)	0.229 (0.083, 0.479)	0.396 (0.250, 0.479)
	LLaVA-Med	0.708 (0.667, 0.792)	0.146 (0.083, 0.208)	0.104 (0.042, 0.146)
	LLaVA 1.5	0.521 (0.438, 0.562)	0.208 (0.062, 0.396)	0.271 (0.104, 0.375)
Treatment	GPT4V	0.408 (0.163, 0.469)	0.184 (0.061, 0.490)	0.367 (0.245, 0.469)
	LLaVA-Med	0.776 (0.694, 0.816)	0.102 (0.041, 0.143)	0.082 (0.041, 0.163)
	LLaVA 1.5	0.551 (0.408, 0.612)	0.184 (0.082, 0.408)	0.224 (0.082, 0.367)
IHC	GPT4V	0.409 (0.273, 0.477)	0.273 (0.114, 0.386)	0.341 (0.295, 0.386)
	LLaVA-Med	0.795 (0.750, 0.955)	0.114 (0.023, 0.159)	0.068 (0.023, 0.136)
	LLaVA 1.5	0.727 (0.568, 0.750)	0.182 (0.114, 0.250)	0.091 (0.045, 0.182)
Molecular	GPT4V	0.390 (0.122, 0.415)	0.220 (0.098, 0.488)	0.390 (0.293, 0.512)
	LLaVA-Med	0.780 (0.707, 0.854)	0.122 (0.049, 0.146)	0.073 (0.049, 0.171)
	LLaVA 1.5	0.512 (0.512, 0.610)	0.195 (0.146, 0.390)	0.220 (0.098, 0.341)
Other Testing	GPT4V	0.667 (0.583, 0.667)	0.167 (0.083, 0.333)	0.167 (0.083, 0.250)
	LLaVA-Med	0.917 (0.917, 0.917)	0.000 (0.000, 0.000)	0.083 (0.000, 0.083)
	LLaVA 1.5	0.750 (0.667, 0.833)	0.000 (0.000, 0.250)	0.167 (0.083, 0.250)

Supplementary Data Table 57: Head-to-head performance of PathChat against other MLLMs on PathQABench open-ended questions, stratified by sub-category and restricted to successful GPT4V queries. For each open-ended question, 7 pathologists independently ranked the outputs of PathChat and competing models (GPT4V, LLaVA-Med, LLaVA 1.5), blinded to which model produced which response (see **MLLM evaluation** for more details). For each pathologist's evaluation, we compute the win/tie/lose rate of PathChat against said model for the 260 open-ended questions. We report the median win/tie/lose rates across the evaluations of 7 pathologists. See **Supplementary Data Table 58–64** for metrics from each individual pathologist. Lose: said model is ranked higher than PathChat; Tie: PathChat is tied with the model in ranking; Win: PathChat is ranked higher than the model. 95% confidence intervals from bootstrapping are included in parentheses. Due to guardrails implemented by GPT4V, 38 / 260 queries to the API yielded unsuccessful answers for PathQABench-Public (a maximum number of 3 attempts were made for each question). We report performance of each model after removing obviously unsuccessful queries for GPT4V here. For more details see **Evaluation of GPT4V** in **Methods**.

Category	PathChat *vs.* model	Win	Tie	Lose
Microscopic	GPT4V	0.736 (0.623, 0.849)	0.151 (0.057, 0.245)	0.113 (0.038, 0.208)
	LLaVA-Med	0.755 (0.642, 0.868)	0.189 (0.094, 0.283)	0.057 (0.000, 0.132)
	LLaVA 1.5	0.774 (0.660, 0.868)	0.151 (0.057, 0.245)	0.075 (0.019, 0.151)
Differentiation	GPT4V	0.636 (0.409, 0.818)	0.182 (0.045, 0.364)	0.182 (0.045, 0.364)
	LLaVA-Med	0.636 (0.409, 0.818)	0.182 (0.045, 0.364)	0.182 (0.045, 0.364)
	LLaVA 1.5	0.636 (0.409, 0.818)	0.273 (0.091, 0.455)	0.091 (0.000, 0.227)
Grading	GPT4V	0.522 (0.304, 0.739)	0.304 (0.130, 0.479)	0.174 (0.043, 0.348)
	LLaVA-Med	0.565 (0.348, 0.783)	0.348 (0.174, 0.565)	0.087 (0.000, 0.217)
	LLaVA 1.5	0.522 (0.304, 0.739)	0.304 (0.130, 0.479)	0.174 (0.043, 0.348)
Diagnosis	GPT4V	0.757 (0.662, 0.851)	0.135 (0.068, 0.216)	0.108 (0.041, 0.176)
	LLaVA-Med	0.784 (0.689, 0.865)	0.162 (0.081, 0.243)	0.054 (0.014, 0.108)
	LLaVA 1.5	0.743 (0.649, 0.838)	0.176 (0.095, 0.270)	0.081 (0.027, 0.149)
Risk factors	GPT4V	0.625 (0.375, 1.000)	0.250 (0.000, 0.625)	0.125 (0.000, 0.375)
	LLaVA-Med	0.625 (0.375, 1.000)	0.375 (0.000, 0.625)	0.000 (0.000, 0.000)
	LLaVA 1.5	0.375 (0.125, 0.625)	0.500 (0.247, 0.875)	0.125 (0.000, 0.375)
Prognosis	GPT4V	0.458 (0.312, 0.604)	0.292 (0.167, 0.417)	0.250 (0.125, 0.375)
	LLaVA-Med	0.792 (0.667, 0.896)	0.125 (0.042, 0.208)	0.083 (0.021, 0.167)
	LLaVA 1.5	0.438 (0.292, 0.583)	0.292 (0.167, 0.417)	0.271 (0.146, 0.396)
Treatment	GPT4V	0.388 (0.265, 0.510)	0.367 (0.245, 0.490)	0.245 (0.142, 0.367)
	LLaVA-Med	0.816 (0.694, 0.918)	0.102 (0.020, 0.204)	0.082 (0.020, 0.163)
	LLaVA 1.5	0.408 (0.286, 0.551)	0.367 (0.224, 0.510)	0.224 (0.102, 0.347)
IHC	GPT4V	0.409 (0.273, 0.568)	0.273 (0.136, 0.409)	0.318 (0.182, 0.455)
	LLaVA-Med	0.818 (0.705, 0.932)	0.068 (0.000, 0.159)	0.114 (0.023, 0.205)
	LLaVA 1.5	0.773 (0.636, 0.886)	0.182 (0.068, 0.295)	0.045 (0.000, 0.114)
Molecular	GPT4V	0.415 (0.268, 0.561)	0.293 (0.146, 0.439)	0.293 (0.146, 0.439)
	LLaVA-Med	0.854 (0.732, 0.951)	0.073 (0.000, 0.171)	0.073 (0.000, 0.146)
	LLaVA 1.5	0.512 (0.366, 0.659)	0.268 (0.146, 0.390)	0.220 (0.098, 0.341)
Other Testing	GPT4V	0.583 (0.333, 0.833)	0.250 (0.000, 0.500)	0.167 (0.000, 0.417)
	LLaVA-Med	0.917 (0.750, 1.000)	0.000 (0.000, 0.000)	0.083 (0.000, 0.250)
	LLaVA 1.5	0.583 (0.331, 0.833)	0.333 (0.083, 0.583)	0.083 (0.000, 0.250)

Supplementary Data Table 58: Head-to-head performance of PathChat against other MLLMs on PathQABench open-ended questions as evaluated by pathologist 1, stratified by sub-category and restricted to successful GPT4V queries. For each open-ended question, 7 pathologists independently ranked the outputs of PathChat and competing models (GPT4V, LLaVA-Med, LLaVA 1.5), blinded to which model produced which response (see **MLLM evaluation** for more details). For each pathologist's evaluation, we compute the win/tie/lose rate of PathChat against said model for the 260 open-ended questions. We report the win/tie/lose rates according to the assessment of pathologist 1. Lose: said model is ranked higher than PathChat; Tie: PathChat is tied with the model in ranking; Win: PathChat is ranked higher than the model. 95% confidence intervals from bootstrapping are included in parentheses. $*$Note that due to guardrails implemented by GPT4V, 38 / 260 queries to the API yielded unsuccessful answers for PathQABench-Public (a maximum number of 3 attempts were made for each question). We report performance of each model after removing obviously unsuccessful queries for GPT4V here. For more details see **Evaluation of GPT4V** in **Methods**.

Category	PathChat *vs.* model	Win	Tie	Lose
Microscopic	GPT4V	0.698 (0.566, 0.811)	0.151 (0.075, 0.245)	0.151 (0.057, 0.264)
	LLaVA-Med	0.774 (0.642, 0.887)	0.113 (0.038, 0.208)	0.113 (0.038, 0.208)
	LLaVA 1.5	0.811 (0.698, 0.906)	0.151 (0.057, 0.264)	0.038 (0.000, 0.094)
Differentiation	GPT4V	0.500 (0.318, 0.683)	0.364 (0.182, 0.545)	0.136 (0.000, 0.273)
	LLaVA-Med	0.591 (0.408, 0.773)	0.273 (0.091, 0.500)	0.136 (0.000, 0.273)
	LLaVA 1.5	0.636 (0.409, 0.818)	0.273 (0.091, 0.455)	0.091 (0.000, 0.227)
Grading	GPT4V	0.522 (0.304, 0.739)	0.261 (0.087, 0.435)	0.217 (0.043, 0.391)
	LLaVA-Med	0.522 (0.304, 0.739)	0.304 (0.130, 0.522)	0.174 (0.043, 0.348)
	LLaVA 1.5	0.565 (0.348, 0.739)	0.261 (0.087, 0.435)	0.174 (0.043, 0.348)
Diagnosis	GPT4V	0.662 (0.554, 0.757)	0.216 (0.135, 0.311)	0.122 (0.041, 0.203)
	LLaVA-Med	0.743 (0.635, 0.838)	0.216 (0.135, 0.311)	0.041 (0.000, 0.095)
	LLaVA 1.5	0.784 (0.676, 0.865)	0.162 (0.081, 0.243)	0.054 (0.014, 0.108)
Risk factors	GPT4V	0.125 (0.000, 0.375)	0.500 (0.125, 0.875)	0.375 (0.122, 0.750)
	LLaVA-Med	0.625 (0.250, 1.000)	0.250 (0.000, 0.503)	0.125 (0.000, 0.375)
	LLaVA 1.5	0.375 (0.000, 0.750)	0.500 (0.125, 0.875)	0.125 (0.000, 0.375)
Prognosis	GPT4V	0.208 (0.104, 0.333)	0.479 (0.354, 0.625)	0.312 (0.188, 0.438)
	LLaVA-Med	0.750 (0.624, 0.855)	0.146 (0.062, 0.250)	0.104 (0.021, 0.208)
	LLaVA 1.5	0.521 (0.396, 0.667)	0.396 (0.270, 0.521)	0.083 (0.021, 0.167)
Treatment	GPT4V	0.163 (0.061, 0.266)	0.490 (0.347, 0.633)	0.347 (0.204, 0.490)
	LLaVA-Med	0.796 (0.673, 0.898)	0.102 (0.041, 0.184)	0.102 (0.020, 0.204)
	LLaVA 1.5	0.510 (0.367, 0.653)	0.408 (0.265, 0.551)	0.082 (0.020, 0.163)
IHC	GPT4V	0.273 (0.159, 0.409)	0.386 (0.250, 0.545)	0.341 (0.205, 0.477)
	LLaVA-Med	0.795 (0.659, 0.909)	0.136 (0.045, 0.250)	0.068 (0.000, 0.159)
	LLaVA 1.5	0.727 (0.591, 0.841)	0.182 (0.091, 0.295)	0.091 (0.000, 0.182)
Molecular	GPT4V	0.122 (0.024, 0.220)	0.488 (0.341, 0.634)	0.390 (0.244, 0.537)
	LLaVA-Med	0.805 (0.683, 0.927)	0.122 (0.024, 0.244)	0.073 (0.000, 0.171)
	LLaVA 1.5	0.512 (0.366, 0.659)	0.390 (0.244, 0.537)	0.098 (0.024, 0.195)
Other Testing	GPT4V	0.667 (0.417, 0.917)	0.083 (0.000, 0.250)	0.250 (0.000, 0.500)
	LLaVA-Med	0.833 (0.583, 1.000)	0.167 (0.000, 0.417)	0.000 (0.000, 0.000)
	LLaVA 1.5	0.750 (0.500, 1.000)	0.000 (0.000, 0.000)	0.250 (0.000, 0.500)

Supplementary Data Table 59: Head-to-head performance of PathChat against other MLLMs on PathQABench open-ended questions as evaluated by pathologist 2, stratified by sub-category and restricted to successful GPT4V queries. For each open-ended question, 7 pathologists independently ranked the outputs of PathChat and competing models (GPT4V, LLaVA-Med, LLaVA 1.5), blinded to which model produced which response (see **MLLM evaluation** for more details). For each pathologist's evaluation, we compute the win/tie/lose rate of PathChat against said model for the 260 open-ended questions. We report the win/tie/lose rates according to the assessment of pathologist 2. Lose: said model is ranked higher than PathChat; Tie: PathChat is tied with the model in ranking; Win: PathChat is ranked higher than the model. 95% confidence intervals from bootstrapping are included in parentheses. ∗Note that due to guardrails implemented by GPT4V, 38 / 260 queries to the API yielded unsuccessful answers for PathQABench-Public (a maximum number of 3 attempts were made for each question). We report performance of each model after removing obviously unsuccessful queries for GPT4V here. For more details see **Evaluation of GPT4V** in **Methods**.

Category	PathChat *vs.* model	Win	Tie	Lose
Microscopic	GPT4V	0.717 (0.604, 0.830)	0.075 (0.019, 0.151)	0.208 (0.113, 0.321)
	LLaVA-Med	0.811 (0.698, 0.906)	0.075 (0.019, 0.151)	0.113 (0.038, 0.208)
	LLaVA 1.5	0.811 (0.698, 0.906)	0.132 (0.056, 0.226)	0.057 (0.000, 0.132)
Differentiation	GPT4V	0.682 (0.500, 0.864)	0.136 (0.000, 0.273)	0.182 (0.045, 0.318)
	LLaVA-Med	0.636 (0.455, 0.818)	0.136 (0.000, 0.273)	0.227 (0.045, 0.409)
	LLaVA 1.5	0.636 (0.455, 0.818)	0.273 (0.091, 0.455)	0.091 (0.000, 0.227)
Grading	GPT4V	0.565 (0.391, 0.783)	0.130 (0.000, 0.261)	0.304 (0.130, 0.478)
	LLaVA-Med	0.609 (0.434, 0.783)	0.087 (0.000, 0.217)	0.304 (0.130, 0.478)
	LLaVA 1.5	0.565 (0.348, 0.739)	0.261 (0.087, 0.435)	0.174 (0.043, 0.348)
Diagnosis	GPT4V	0.703 (0.595, 0.797)	0.162 (0.081, 0.257)	0.135 (0.068, 0.216)
	LLaVA-Med	0.757 (0.649, 0.851)	0.149 (0.068, 0.243)	0.095 (0.027, 0.176)
	LLaVA 1.5	0.770 (0.662, 0.851)	0.149 (0.068, 0.230)	0.081 (0.027, 0.149)
Risk factors	GPT4V	0.375 (0.000, 0.750)	0.000 (0.000, 0.000)	0.625 (0.250, 1.000)
	LLaVA-Med	0.750 (0.500, 1.000)	0.250 (0.000, 0.500)	0.000 (0.000, 0.000)
	LLaVA 1.5	0.625 (0.250, 0.875)	0.125 (0.000, 0.375)	0.250 (0.000, 0.625)
Prognosis	GPT4V	0.458 (0.312, 0.604)	0.062 (0.000, 0.125)	0.479 (0.354, 0.604)
	LLaVA-Med	0.708 (0.583, 0.833)	0.146 (0.042, 0.250)	0.146 (0.062, 0.250)
	LLaVA 1.5	0.521 (0.395, 0.667)	0.062 (0.000, 0.146)	0.417 (0.291, 0.562)
Treatment	GPT4V	0.449 (0.306, 0.592)	0.041 (0.000, 0.102)	0.510 (0.367, 0.653)
	LLaVA-Med	0.776 (0.653, 0.878)	0.143 (0.061, 0.245)	0.082 (0.020, 0.163)
	LLaVA 1.5	0.551 (0.429, 0.694)	0.082 (0.020, 0.163)	0.367 (0.224, 0.510)
IHC	GPT4V	0.432 (0.295, 0.591)	0.182 (0.068, 0.296)	0.386 (0.249, 0.545)
	LLaVA-Med	0.750 (0.614, 0.886)	0.114 (0.023, 0.227)	0.136 (0.045, 0.250)
	LLaVA 1.5	0.727 (0.591, 0.841)	0.205 (0.091, 0.318)	0.068 (0.000, 0.159)
Molecular	GPT4V	0.390 (0.244, 0.537)	0.098 (0.024, 0.195)	0.512 (0.341, 0.659)
	LLaVA-Med	0.780 (0.659, 0.902)	0.146 (0.049, 0.244)	0.073 (0.000, 0.171)
	LLaVA 1.5	0.512 (0.366, 0.659)	0.146 (0.049, 0.268)	0.341 (0.195, 0.488)
Other Testing	GPT4V	0.583 (0.333, 0.833)	0.083 (0.000, 0.250)	0.333 (0.083, 0.583)
	LLaVA-Med	0.917 (0.750, 1.000)	0.000 (0.000, 0.000)	0.083 (0.000, 0.250)
	LLaVA 1.5	0.833 (0.583, 1.000)	0.000 (0.000, 0.000)	0.167 (0.000, 0.417)

Supplementary Data Table 60: Head-to-head performance of PathChat against other MLLMs on PathQABench open-ended questions as evaluated by pathologist 3, stratified by sub-category and restricted to successful GPT4V queries. For each open-ended question, 7 pathologists independently ranked the outputs of PathChat and competing models (GPT4V, LLaVA-Med, LLaVA 1.5), blinded to which model produced which response (see **MLLM evaluation** for more details). For each pathologist's evaluation, we compute the win/tie/lose rate of PathChat against said model for the 260 open-ended questions. We report the win/tie/lose rates according to the assessment of pathologist 3. Lose: said model is ranked higher than PathChat; Tie: PathChat is tied with the model in ranking; Win: PathChat is ranked higher than the model. 95% confidence intervals from bootstrapping are included in parentheses. ∗Note that due to guardrails implemented by GPT4V, 38 / 260 queries to the API yielded unsuccessful answers for PathQABench-Public (a maximum number of 3 attempts were made for each question). We report performance of each model after removing obviously unsuccessful queries for GPT4V here. For more details see **Evaluation of GPT4V** in **Methods**.

Category	PathChat *vs.* model	Win	Tie	Lose
Microscopic	GPT4V	0.717 (0.585, 0.830)	0.151 (0.057, 0.264)	0.132 (0.057, 0.226)
	LLaVA-Med	0.736 (0.622, 0.849)	0.189 (0.094, 0.302)	0.075 (0.019, 0.151)
	LLaVA 1.5	0.736 (0.623, 0.849)	0.226 (0.132, 0.340)	0.038 (0.000, 0.094)
Differentiation	GPT4V	0.591 (0.409, 0.773)	0.364 (0.182, 0.545)	0.045 (0.000, 0.136)
	LLaVA-Med	0.545 (0.364, 0.773)	0.364 (0.182, 0.545)	0.091 (0.000, 0.227)
	LLaVA 1.5	0.636 (0.455, 0.818)	0.273 (0.091, 0.455)	0.091 (0.000, 0.227)
Grading	GPT4V	0.609 (0.391, 0.826)	0.261 (0.087, 0.435)	0.130 (0.000, 0.304)
	LLaVA-Med	0.565 (0.348, 0.783)	0.261 (0.087, 0.435)	0.174 (0.043, 0.348)
	LLaVA 1.5	0.565 (0.391, 0.739)	0.391 (0.217, 0.565)	0.043 (0.000, 0.130)
Diagnosis	GPT4V	0.622 (0.514, 0.717)	0.149 (0.068, 0.230)	0.230 (0.135, 0.324)
	LLaVA-Med	0.662 (0.554, 0.770)	0.257 (0.162, 0.351)	0.081 (0.027, 0.149)
	LLaVA 1.5	0.689 (0.581, 0.784)	0.230 (0.135, 0.324)	0.081 (0.027, 0.149)
Risk factors	GPT4V	0.375 (0.125, 0.750)	0.125 (0.000, 0.375)	0.500 (0.125, 0.875)
	LLaVA-Med	0.750 (0.500, 1.000)	0.000 (0.000, 0.000)	0.250 (0.000, 0.500)
	LLaVA 1.5	0.625 (0.250, 0.875)	0.125 (0.000, 0.375)	0.250 (0.000, 0.625)
Prognosis	GPT4V	0.375 (0.250, 0.521)	0.229 (0.125, 0.354)	0.396 (0.250, 0.521)
	LLaVA-Med	0.667 (0.542, 0.792)	0.208 (0.104, 0.333)	0.125 (0.042, 0.229)
	LLaVA 1.5	0.562 (0.438, 0.708)	0.208 (0.104, 0.312)	0.229 (0.125, 0.333)
Treatment	GPT4V	0.408 (0.285, 0.531)	0.184 (0.082, 0.306)	0.408 (0.286, 0.551)
	LLaVA-Med	0.694 (0.571, 0.816)	0.143 (0.041, 0.265)	0.163 (0.061, 0.266)
	LLaVA 1.5	0.612 (0.490, 0.755)	0.184 (0.082, 0.286)	0.204 (0.102, 0.327)
IHC	GPT4V	0.341 (0.205, 0.500)	0.318 (0.182, 0.455)	0.341 (0.205, 0.477)
	LLaVA-Med	0.591 (0.432, 0.728)	0.273 (0.159, 0.409)	0.136 (0.045, 0.250)
	LLaVA 1.5	0.568 (0.432, 0.705)	0.250 (0.136, 0.386)	0.182 (0.068, 0.295)
Molecular	GPT4V	0.390 (0.244, 0.537)	0.220 (0.098, 0.341)	0.390 (0.244, 0.537)
	LLaVA-Med	0.707 (0.585, 0.829)	0.122 (0.024, 0.244)	0.171 (0.049, 0.293)
	LLaVA 1.5	0.610 (0.463, 0.756)	0.195 (0.073, 0.317)	0.195 (0.098, 0.293)
Other Testing	GPT4V	0.750 (0.500, 0.917)	0.167 (0.000, 0.417)	0.083 (0.000, 0.250)
	LLaVA-Med	0.917 (0.750, 1.000)	0.000 (0.000, 0.000)	0.083 (0.000, 0.250)
	LLaVA 1.5	0.833 (0.583, 1.000)	0.000 (0.000, 0.000)	0.167 (0.000, 0.417)

Supplementary Data Table 61: Head-to-head performance of PathChat against other MLLMs on PathQABench open-ended questions as evaluated by pathologist 4, stratified by sub-category and restricted to successful GPT4V queries. For each open-ended question, 7 pathologists independently ranked the outputs of PathChat and competing models (GPT4V, LLaVA-Med, LLaVA 1.5), blinded to which model produced which response (see **MLLM evaluation** for more details). For each pathologist's evaluation, we compute the win/tie/lose rate of PathChat against said model for the 260 open-ended questions. We report the win/tie/lose rates according to the assessment of pathologist 4. Lose: said model is ranked higher than PathChat; Tie: PathChat is tied with the model in ranking; Win: PathChat is ranked higher than the model. 95% confidence intervals from bootstrapping are included in parentheses. ∗Note that due to guardrails implemented by GPT4V, 38 / 260 queries to the API yielded unsuccessful answers for PathQABench-Public (a maximum number of 3 attempts were made for each question). We report performance of each model after removing obviously unsuccessful queries for GPT4V here. For more details see **Evaluation of GPT4V** in **Methods**.

Category	PathChat *vs.* model	Win	Tie	Lose
Microscopic	GPT4V	0.698 (0.585, 0.811)	0.189 (0.094, 0.302)	0.113 (0.038, 0.208)
	LLaVA-Med	0.755 (0.642, 0.868)	0.132 (0.056, 0.226)	0.113 (0.038, 0.208)
	LLaVA 1.5	0.792 (0.679, 0.906)	0.094 (0.019, 0.170)	0.113 (0.038, 0.208)
Differentiation	GPT4V	0.727 (0.545, 0.909)	0.182 (0.045, 0.364)	0.091 (0.000, 0.227)
	LLaVA-Med	0.682 (0.500, 0.864)	0.182 (0.045, 0.364)	0.136 (0.000, 0.273)
	LLaVA 1.5	0.682 (0.500, 0.864)	0.227 (0.045, 0.409)	0.091 (0.000, 0.227)
Grading	GPT4V	0.565 (0.348, 0.739)	0.261 (0.087, 0.435)	0.174 (0.043, 0.348)
	LLaVA-Med	0.609 (0.391, 0.783)	0.217 (0.043, 0.391)	0.174 (0.043, 0.348)
	LLaVA 1.5	0.565 (0.391, 0.739)	0.348 (0.174, 0.522)	0.087 (0.000, 0.217)
Diagnosis	GPT4V	0.757 (0.662, 0.838)	0.176 (0.095, 0.270)	0.068 (0.014, 0.135)
	LLaVA-Med	0.730 (0.622, 0.824)	0.189 (0.108, 0.284)	0.081 (0.027, 0.149)
	LLaVA 1.5	0.730 (0.635, 0.824)	0.176 (0.095, 0.270)	0.095 (0.027, 0.162)
Risk factors	GPT4V	0.125 (0.000, 0.375)	0.875 (0.625, 1.000)	0.000 (0.000, 0.000)
	LLaVA-Med	0.750 (0.500, 1.000)	0.250 (0.000, 0.500)	0.000 (0.000, 0.000)
	LLaVA 1.5	0.250 (0.000, 0.500)	0.750 (0.500, 1.000)	0.000 (0.000, 0.000)
Prognosis	GPT4V	0.167 (0.062, 0.292)	0.750 (0.625, 0.875)	0.083 (0.021, 0.167)
	LLaVA-Med	0.583 (0.438, 0.729)	0.375 (0.229, 0.521)	0.042 (0.000, 0.104)
	LLaVA 1.5	0.438 (0.292, 0.583)	0.458 (0.312, 0.604)	0.104 (0.021, 0.188)
Treatment	GPT4V	0.122 (0.041, 0.224)	0.816 (0.714, 0.918)	0.061 (0.000, 0.122)
	LLaVA-Med	0.694 (0.571, 0.816)	0.286 (0.163, 0.408)	0.020 (0.000, 0.061)
	LLaVA 1.5	0.388 (0.265, 0.531)	0.531 (0.388, 0.673)	0.082 (0.020, 0.163)
IHC	GPT4V	0.273 (0.136, 0.409)	0.705 (0.568, 0.841)	0.023 (0.000, 0.068)
	LLaVA-Med	0.795 (0.659, 0.909)	0.159 (0.068, 0.273)	0.045 (0.000, 0.114)
	LLaVA 1.5	0.477 (0.341, 0.614)	0.523 (0.386, 0.659)	0.000 (0.000, 0.000)
Molecular	GPT4V	0.122 (0.024, 0.220)	0.780 (0.634, 0.902)	0.098 (0.024, 0.195)
	LLaVA-Med	0.707 (0.561, 0.830)	0.293 (0.170, 0.439)	0.000 (0.000, 0.000)
	LLaVA 1.5	0.341 (0.195, 0.488)	0.561 (0.415, 0.707)	0.098 (0.024, 0.195)
Other Testing	GPT4V	0.333 (0.083, 0.583)	0.583 (0.250, 0.833)	0.083 (0.000, 0.250)
	LLaVA-Med	0.917 (0.750, 1.000)	0.000 (0.000, 0.000)	0.083 (0.000, 0.250)
	LLaVA 1.5	0.667 (0.417, 0.917)	0.250 (0.000, 0.500)	0.083 (0.000, 0.250)

Supplementary Data Table 62: Head-to-head performance of PathChat against other MLLMs on PathQABench open-ended questions as evaluated by pathologist 5, stratified by sub-category and restricted to successful GPT4V queries. For each open-ended question, 7 pathologists independently ranked the outputs of PathChat and competing models (GPT4V, LLaVA-Med, LLaVA 1.5), blinded to which model produced which response (see **MLLM evaluation** for more details). For each pathologist's evaluation, we compute the win/tie/lose rate of PathChat against said model for the 260 open-ended questions. We report the win/tie/lose rates according to the assessment of pathologist 5. Lose: said model is ranked higher than PathChat; Tie: PathChat is tied with the model in ranking; Win: PathChat is ranked higher than the model. 95% confidence intervals from bootstrapping are included in parentheses. ∗Note that due to guardrails implemented by GPT4V, 38 / 260 queries to the API yielded unsuccessful answers for PathQABench-Public (a maximum number of 3 attempts were made for each question). We report performance of each model after removing obviously unsuccessful queries for GPT4V here. For more details see **Evaluation of GPT4V** in **Methods**.

Category	PathChat *vs.* model	Win	Tie	Lose
Microscopic	GPT4V	0.717 (0.585, 0.830)	0.019 (0.000, 0.057)	0.264 (0.151, 0.377)
	LLaVA-Med	0.698 (0.566, 0.811)	0.057 (0.000, 0.132)	0.245 (0.132, 0.377)
	LLaVA 1.5	0.755 (0.642, 0.868)	0.075 (0.019, 0.151)	0.170 (0.075, 0.283)
Differentiation	GPT4V	0.773 (0.591, 0.955)	0.045 (0.000, 0.136)	0.182 (0.045, 0.364)
	LLaVA-Med	0.545 (0.318, 0.727)	0.045 (0.000, 0.136)	0.409 (0.227, 0.636)
	LLaVA 1.5	0.591 (0.409, 0.818)	0.045 (0.000, 0.136)	0.364 (0.182, 0.545)
Grading	GPT4V	0.696 (0.478, 0.870)	0.087 (0.000, 0.217)	0.217 (0.043, 0.391)
	LLaVA-Med	0.652 (0.478, 0.826)	0.130 (0.000, 0.304)	0.217 (0.087, 0.391)
	LLaVA 1.5	0.522 (0.304, 0.739)	0.130 (0.000, 0.261)	0.348 (0.130, 0.565)
Diagnosis	GPT4V	0.743 (0.649, 0.838)	0.027 (0.000, 0.068)	0.230 (0.149, 0.324)
	LLaVA-Med	0.743 (0.649, 0.838)	0.081 (0.027, 0.149)	0.176 (0.095, 0.257)
	LLaVA 1.5	0.703 (0.608, 0.797)	0.108 (0.041, 0.189)	0.189 (0.108, 0.284)
Risk factors	GPT4V	0.375 (0.000, 0.750)	0.000 (0.000, 0.000)	0.625 (0.250, 1.000)
	LLaVA-Med	0.625 (0.250, 0.875)	0.250 (0.000, 0.500)	0.125 (0.000, 0.375)
	LLaVA 1.5	0.500 (0.125, 0.750)	0.250 (0.000, 0.625)	0.250 (0.000, 0.625)
Prognosis	GPT4V	0.479 (0.333, 0.625)	0.125 (0.042, 0.229)	0.396 (0.250, 0.542)
	LLaVA-Med	0.667 (0.521, 0.792)	0.083 (0.021, 0.167)	0.250 (0.125, 0.375)
	LLaVA 1.5	0.562 (0.417, 0.708)	0.062 (0.000, 0.146)	0.375 (0.250, 0.521)
Treatment	GPT4V	0.510 (0.367, 0.633)	0.122 (0.041, 0.224)	0.367 (0.245, 0.510)
	LLaVA-Med	0.714 (0.571, 0.837)	0.041 (0.000, 0.102)	0.245 (0.122, 0.367)
	LLaVA 1.5	0.551 (0.408, 0.694)	0.061 (0.000, 0.122)	0.388 (0.265, 0.511)
IHC	GPT4V	0.591 (0.455, 0.727)	0.114 (0.023, 0.205)	0.295 (0.159, 0.432)
	LLaVA-Med	0.977 (0.932, 1.000)	0.023 (0.000, 0.068)	0.000 (0.000, 0.000)
	LLaVA 1.5	0.727 (0.591, 0.841)	0.068 (0.000, 0.159)	0.205 (0.091, 0.318)
Molecular	GPT4V	0.463 (0.317, 0.610)	0.146 (0.049, 0.268)	0.390 (0.244, 0.537)
	LLaVA-Med	0.756 (0.610, 0.878)	0.049 (0.000, 0.122)	0.195 (0.073, 0.317)
	LLaVA 1.5	0.585 (0.438, 0.732)	0.024 (0.000, 0.073)	0.390 (0.244, 0.537)
Other Testing	GPT4V	0.667 (0.417, 0.917)	0.333 (0.083, 0.583)	0.000 (0.000, 0.000)
	LLaVA-Med	1.000 (1.000, 1.000)	0.000 (0.000, 0.000)	0.000 (0.000, 0.000)
	LLaVA 1.5	0.750 (0.500, 0.919)	0.083 (0.000, 0.250)	0.167 (0.000, 0.417)

Supplementary Data Table 63: Head-to-head performance of PathChat against other MLLMs on PathQABench open-ended questions as evaluated by pathologist 6, stratified by sub-category and restricted to successful GPT4V queries. For each open-ended question, 7 pathologists independently ranked the outputs of PathChat and competing models (GPT4V, LLaVA-Med, LLaVA 1.5), blinded to which model produced which response (see **MLLM evaluation** for more details). For each pathologist's evaluation, we compute the win/tie/lose rate of PathChat against said model for the 260 open-ended questions. We report the win/tie/lose rates according to the assessment of pathologist 6. Lose: said model is ranked higher than PathChat; Tie: PathChat is tied with the model in ranking; Win: PathChat is ranked higher than the model. 95% confidence intervals from bootstrapping are included in parentheses. ∗Note that due to guardrails implemented by GPT4V, 38 / 260 queries to the API yielded unsuccessful answers for PathQABench-Public (a maximum number of 3 attempts were made for each question). We report performance of each model after removing obviously unsuccessful queries for GPT4V here. For more details see **Evaluation of GPT4V** in **Methods**.

Category	PathChat *vs.* model	Win	Tie	Lose
Microscopic	GPT4V	0.453 (0.321, 0.604)	0.019 (0.000, 0.057)	0.528 (0.396, 0.660)
	LLaVA-Med	0.868 (0.774, 0.962)	0.057 (0.000, 0.132)	0.075 (0.019, 0.151)
	LLaVA 1.5	0.868 (0.774, 0.962)	0.057 (0.000, 0.132)	0.075 (0.019, 0.151)
Differentiation	GPT4V	0.409 (0.227, 0.636)	0.000 (0.000, 0.000)	0.591 (0.364, 0.773)
	LLaVA-Med	0.818 (0.636, 0.955)	0.091 (0.000, 0.227)	0.091 (0.000, 0.227)
	LLaVA 1.5	0.818 (0.682, 0.955)	0.091 (0.000, 0.227)	0.091 (0.000, 0.227)
Grading	GPT4V	0.522 (0.304, 0.739)	0.000 (0.000, 0.000)	0.478 (0.261, 0.696)
	LLaVA-Med	0.870 (0.696, 1.000)	0.087 (0.000, 0.217)	0.043 (0.000, 0.130)
	LLaVA 1.5	0.870 (0.738, 1.000)	0.000 (0.000, 0.000)	0.130 (0.000, 0.262)
Diagnosis	GPT4V	0.486 (0.378, 0.608)	0.054 (0.014, 0.108)	0.459 (0.338, 0.581)
	LLaVA-Med	0.838 (0.757, 0.905)	0.081 (0.027, 0.149)	0.081 (0.027, 0.149)
	LLaVA 1.5	0.851 (0.770, 0.919)	0.054 (0.014, 0.108)	0.095 (0.041, 0.162)
Risk factors	GPT4V	0.125 (0.000, 0.375)	0.125 (0.000, 0.375)	0.750 (0.500, 1.000)
	LLaVA-Med	0.875 (0.625, 1.000)	0.125 (0.000, 0.375)	0.000 (0.000, 0.000)
	LLaVA 1.5	0.500 (0.125, 0.875)	0.000 (0.000, 0.000)	0.500 (0.125, 0.875)
Prognosis	GPT4V	0.396 (0.250, 0.521)	0.083 (0.021, 0.167)	0.521 (0.375, 0.667)
	LLaVA-Med	0.958 (0.896, 1.000)	0.000 (0.000, 0.000)	0.042 (0.000, 0.104)
	LLaVA 1.5	0.604 (0.479, 0.750)	0.104 (0.021, 0.208)	0.292 (0.187, 0.417)
Treatment	GPT4V	0.469 (0.327, 0.612)	0.061 (0.000, 0.143)	0.469 (0.347, 0.612)
	LLaVA-Med	0.959 (0.898, 1.000)	0.000 (0.000, 0.000)	0.041 (0.000, 0.102)
	LLaVA 1.5	0.633 (0.510, 0.755)	0.102 (0.020, 0.184)	0.265 (0.143, 0.388)
IHC	GPT4V	0.477 (0.341, 0.614)	0.114 (0.023, 0.205)	0.409 (0.273, 0.545)
	LLaVA-Med	0.955 (0.886, 1.000)	0.023 (0.000, 0.068)	0.023 (0.000, 0.068)
	LLaVA 1.5	0.750 (0.614, 0.886)	0.114 (0.045, 0.205)	0.136 (0.045, 0.250)
Molecular	GPT4V	0.390 (0.244, 0.537)	0.073 (0.000, 0.146)	0.537 (0.366, 0.683)
	LLaVA-Med	0.927 (0.853, 1.000)	0.024 (0.000, 0.073)	0.049 (0.000, 0.122)
	LLaVA 1.5	0.610 (0.463, 0.756)	0.146 (0.049, 0.268)	0.244 (0.122, 0.390)
Other Testing	GPT4V	0.667 (0.417, 0.917)	0.167 (0.000, 0.417)	0.167 (0.000, 0.417)
	LLaVA-Med	0.917 (0.750, 1.000)	0.000 (0.000, 0.000)	0.083 (0.000, 0.250)
	LLaVA 1.5	0.750 (0.500, 1.000)	0.000 (0.000, 0.000)	0.250 (0.000, 0.500)

Supplementary Data Table 64: Head-to-head performance of PathChat against other MLLMs on PathQABench open-ended questions as evaluated by pathologist 7, stratified by sub-category and restricted to successful GPT4V queries. For each open-ended question, 7 pathologists independently ranked the outputs of PathChat and competing models (GPT4V, LLaVA-Med, LLaVA 1.5), blinded to which model produced which response (see **MLLM evaluation** for more details). For each pathologist's evaluation, we compute the win/tie/lose rate of PathChat against said model for the 260 open-ended questions. We report the win/tie/lose rates according to the assessment of pathologist 7. Lose: said model is ranked higher than PathChat; Tie: PathChat is tied with the model in ranking; Win: PathChat is ranked higher than the model. 95% confidence intervals from bootstrapping are included in parentheses. ∗Note that due to guardrails implemented by GPT4V, 38 / 260 queries to the API yielded unsuccessful answers for PathQABench-Public (a maximum number of 3 attempts were made for each question). We report performance of each model after removing obviously unsuccessful queries for GPT4V here. For more details see **Evaluation of GPT4V** in **Methods**.
