# Supplementary material for: A multimodal generative AI copilot for human pathology
Source: Nature. 2024 Jun 12;634(8033):466–73. doi: 10.1038/s41586-024-07618-3 (PMC11464372; doi:10.1038/s41586-024-07618-3)
Supplement: Supplementary file 2 — Reporting Summary [file 41586_2024_7618_MOESM2_ESM.pdf]

nature portfolio

Corresponding author(s): Faisal Mahmood

Last updated by author(s): May 3, 2024

Reporting Summary

Nature Portfolio wishes to improve the reproducibility of the work that we publish. This form provides structure for consistency and transparency in reporting. For further information on Nature Portfolio policies, see our Editorial Policies and the Editorial Policy Checklist.

Statistics

For all statistical analyses, confirm that the following items are present in the figure legend, table legend, main text, or Methods section.

n/a	Confirmed	
☐	☒	The exact sample size (*n*) for each experimental group/condition, given as a discrete number and unit of measurement
☐	☒	A statement on whether measurements were taken from distinct samples or whether the same sample was measured repeatedly
☐	☒	The statistical test(s) used AND whether they are one- or two-sided<br>*Only common tests should be described solely by name; describe more complex techniques in the Methods section.*
☐	☒	A description of all covariates tested
☒	☐	A description of any assumptions or corrections, such as tests of normality and adjustment for multiple comparisons
☐	☒	A full description of the statistical parameters including central tendency (e.g. means) or other basic estimates (e.g. regression coefficient) AND variation (e.g. standard deviation) or associated estimates of uncertainty (e.g. confidence intervals)
☐	☒	For null hypothesis testing, the test statistic (e.g. *F*, *t*, *r*) with confidence intervals, effect sizes, degrees of freedom and *P* value noted<br>*Give P values as exact values whenever suitable.*
☒	☐	For Bayesian analysis, information on the choice of priors and Markov chain Monte Carlo settings
☒	☐	For hierarchical and complex designs, identification of the appropriate level for tests and full reporting of outcomes
☒	☐	Estimates of effect sizes (e.g. Cohen's *d*, Pearson's *r*), indicating how they were calculated

Our web collection on statistics for biologists contains articles on many of the points above.

Software and code

Policy information about availability of computer code

Data collection	The image ROIs in PathQABench are hand-selected by a board-certified pathologist using the open-source QuPath (0.4.2) digital viewer software. Question prompts and multiple choice options were compiled using Python (3.10.13) and numpy (1.26.1).
Data analysis	Evaluation of GPT4V (gpt-4-vision-preview) on PathQABench-Public was performed through the official OpenAI API python bindings, openai (1.3.3). All API calls were made in February 2024. Evaluation of LLaVA 1.5 was performed based on the evaluation script provided by the LLaVA (1.1.3) repository: https://github.com/haotian-liu/LLaVA. Evaluation of LLaVA-Med was performed based on the evaluation script provided by the LLaVA-Med (0.1.0) repository: https://github.com/microsoft/LLaVA-Med. The model checkpoints evaluated can be accessed respectively via https://huggingface.co/liuhaotian/llava-v1.5-13b and https://hanoverprod.z21.web.core.windows.net/med_llava/models/llava_med_in_text_60k_ckpt2_delta.zip.

For manuscripts utilizing custom algorithms or software that are central to the research but not yet described in published literature, software must be made available to editors and reviewers. We strongly encourage code deposition in a community repository (e.g. GitHub). See the Nature Portfolio guidelines for submitting code & software for further information.

Data

Policy information about availability of data

All manuscripts must include a data availability statement. This statement should provide the following information, where applicable:

- Accession codes, unique identifiers, or web links for publicly available datasets
- A description of any restrictions on data availability
- For clinical datasets or third party data, please ensure that the statement adheres to our policy

The PubMed Central-OA dataset can be accessed from the NIH PubMed Central website (https://www.ncbi.nlm.nih.gov/pmc/tools/openftlist/). The TCGA WSIs and associated clinical metadata are available from the NIH genomic data commons (https://portal.gdc.cancer.gov). The curated PathQABench-Public benchmark is released for research use and can be accessed through: https://github.com/fedshyvana/pathology_mllm_training. Patient data used in this project was curated with institutional permission through IRB approval for the current study and thus cannot be made publicly available in compliance with patient privacy obligations. All requests for processed data curated internally will be evaluated based on institutional and departmental policies to determine whether the data requested is subject to intellectual property or patient privacy obligations. Data that can be transferred will require a material or data transfer agreement between the institutions and will limit the utility of the data to non-commercial academic research purposes. The exact timeline will depend on the execution of such agreements. Please email all requests to the corresponding author (and also include M.Y.L., mlu16@bwh.harvard.edu).

Research involving human participants, their data, or biological material

Policy information about studies with human participants or human data. See also policy information about sex, gender (identity/presentation), and sexual orientation and race, ethnicity and racism.

Reporting on sex and gender	No covariates relating to sex or gender were collected, used or analyzed in the study.
Reporting on race, ethnicity, or other socially relevant groupings	No covariates regarding race, ethnicity, and other social groupings were collected, used or analyzed in the study.
Population characteristics	No covariates relating to population characteristics were collected, used or analyzed in the study.
Recruitment	The study relied on analysis of pathology images retrospectively and no patients were recruited specifically for this study.
Ethics oversight	Brigham and Women's Hospital IRB committee approved the retrospective analysis of pathology images and corresponding reports.

Note that full information on the approval of the study protocol must also be provided in the manuscript.

Field-specific reporting

Please select the one below that is the best fit for your research. If you are not sure, read the appropriate sections before making your selection.

☒ Life sciences ☐ Behavioural & social sciences ☐ Ecological, evolutionary & environmental sciences

For a reference copy of the document with all sections, see nature.com/documents/nr-reporting-summary-flat.pdf

Life sciences study design

All studies must disclose on these points even when the disclosure is negative.

Sample size	No sample size calculation was performed as with all deep learning studies asymptotic model performance was used to gadge the size of the training dataset.
Data exclusions	For pretraining data, data filtering was performed for each data source individually to ensure quality and relevance for training a pathology-specific vision language assistant. Examples of frequently used heuristics for filtering include the removal of image captions that are overly short (< 12 words) or uninformative and overly generic (e.g. "An H&E image of tumor."). We also removed captions or passages related to animal pathology (keywords include: ``rat", ``pig", etc.) and experimental studies (keywords include: "experimental", "positive control", etc.) using regex pattern matching. We did not apply any special exclusion criteria to the evaluation dataset.
Replication	On PathQABench, the reported performance metrics are deterministically computed using the model predictions and either groundtruth diagnostic labels or human expert feedback. Replication of the test results reported was successful across 5 different attempts of running the evaluation script.
Randomization	In multiple choice questions, for each question, we randomize the relative order of possible answer choices to mitigate potential position bias in models. In human expert evaluation, the relative order of responses by different models are also similarly randomized for each question.
Blinding	In human expert evaluation, the expert pathologists are blinded to which model produced which response.

Reporting for specific materials, systems and methods

We require information from authors about some types of materials, experimental systems and methods used in many studies. Here, indicate whether each material, system or method listed is relevant to your study. If you are not sure if a list item applies to your research, read the appropriate section before selecting a response.

Materials & experimental systems

n/a	Involved in the study
☒	☐ Antibodies
☒	☐ Eukaryotic cell lines
☒	☐ Palaeontology and archaeology
☒	☐ Animals and other organisms
☒	☐ Clinical data
☒	☐ Dual use research of concern
☒	☐ Plants

Methods

n/a	Involved in the study
☒	☐ ChIP-seq
☒	☐ Flow cytometry
☒	☐ MRI-based neuroimaging

Plants

Seed stocks

Report on the source of all seed stocks or other plant material used. If applicable, state the seed stock centre and catalogue number. If plant specimens were collected from the field, describe the collection location, date and sampling procedures.

Novel plant genotypes

Describe the methods by which all novel plant genotypes were produced. This includes those generated by transgenic approaches, gene editing, chemical/radiation-based mutagenesis and hybridization. For transgenic lines, describe the transformation method, the number of independent lines analyzed and the generation upon which experiments were performed. For gene-edited lines, describe the editor used, the endogenous sequence targeted for editing, the targeting guide RNA sequence (if applicable) and how the editor was applied.

Authentication

Describe any authentication procedures for each seed stock used or novel genotype generated. Describe any experiments used to assess the effect of a mutation and, where applicable, how potential secondary effects (e.g. second site T-DNA insertions, mosiacism, off-target gene editing) were examined.
